# Transcriptome innovations in primates revealed by single-molecule long-read sequencing

**DOI:** 10.1101/gr.276395.121

**Published:** 2022-08

**Authors:** Luis Ferrández-Peral, Xiaoyu Zhan, Marina Alvarez-Estape, Cristina Chiva, Paula Esteller-Cucala, Raquel García-Pérez, Eva Julià, Esther Lizano, Òscar Fornas, Eduard Sabidó, Qiye Li, Tomàs Marquès-Bonet, David Juan, Guojie Zhang

**Affiliations:** 1 Institute of Evolutionary Biology (UPF-CSIC), PRBB, 08003 Barcelona, Spain;; 2 BGI-Shenzhen, Shenzhen 518083, China;; 3 Center for Genomic Regulation (CRG), Barcelona Institute of Science and Technology (BIST), 08003 Barcelona, Spain;; 4 Universitat Pompeu Fabra (UPF), 08003 Barcelona, Spain;; 5 Institut Català de Paleontologia Miquel Crusafont, Universitat Autònoma de Barcelona, Cerdanyola del Vallès, 08193 Barcelona, Spain;; 6 College of Life Sciences, University of Chinese Academy of Sciences, Beijing 100049, China;; 7 Institució Catalana de Recerca i Estudis Avançats (ICREA), 08010 Barcelona, Spain;; 8 CNAG-CRG, Center for Genomic Regulation (CRG), Barcelona Institute of Science and Technology (BIST), 08028 Barcelona, Spain;; 9 State Key Laboratory of Genetic Resources and Evolution, Kunming Institute of Zoology, Chinese Academy of Sciences, Kunming 650223, China;; 10 Section for Ecology and Evolution, Department of Biology, University of Copenhagen, DK-2100 Copenhagen 2200, Denmark;; 11 Evolutionary and Organismal Biology Research Center, School of Medicine, Zhejiang University, Hangzhou 310058, China

## Abstract

Transcriptomic diversity greatly contributes to the fundamentals of disease, lineage-specific biology, and environmental adaptation. However, much of the actual isoform repertoire contributing to shaping primate evolution remains unknown. Here, we combined deep long- and short-read sequencing complemented with mass spectrometry proteomics in a panel of lymphoblastoid cell lines (LCLs) from human, three other great apes, and rhesus macaque, producing the largest full-length isoform catalog in primates to date. Around half of the captured isoforms are not annotated in their reference genomes, significantly expanding the gene models in primates. Furthermore, our comparative analyses unveil hundreds of transcriptomic innovations and isoform usage changes related to immune function and immunological disorders. The confluence of these evolutionary innovations with signals of positive selection and their limited impact in the proteome points to changes in alternative splicing in genes involved in immune response as an important target of recent regulatory divergence in primates.

The vast complexity of eukaryotic transcriptomes arises from the co-occurrence of multiple RNA processing events, leading to the generation of different isoforms encoded by the same gene. Particularly, the fine-tuning of alternative splicing (AS) is a critical mechanism underlying disease and phenotypic evolution (Scotti and Swanson 2016; Bush et al. 2017). Hence, previous research on the conservation of RNA processing events in humans and nonhuman primates (NHPs) has revealed the functional importance of transcriptomic diversity (Blekhman et al. 2010; Mazin et al. 2013, 2018; Zhang et al. 2017; Dougherty et al. 2018; Kronenberg et al. 2018; Xiong et al. 2018; Mittleman et al. 2021). On the other hand, the potential of increasingly complex transcriptomes to produce alternative protein isoforms is under intense debate as most of the predicted proteins remain undetected (Sheynkman et al. 2013; Abascal et al. 2015; Liu et al. 2017; Tress et al. 2017; Carlyle et al. 2018; Agosto et al. 2019; Lau et al. 2019), and alternative transcripts can carry out other regulatory functions (Mignone et al. 2002).

Most previous comparative studies are based on short-read RNA-seq, which cannot capture the actual isoform landscapes in different species. The recent emergence of full-length isoform sequencing overcomes this limitation and has contributed to the refinement of isoform repertoires in model and nonmodel organisms (Byrne et al. 2019). Thus, single-molecule sequencing in combination with short-read RNA-seq and proteomic data appears as a powerful tool to disentangle the recent primate evolution of transcriptomes with isoform resolution, especially with the most recent high-quality genome assemblies for NHPs (Kronenberg et al. 2018; He et al. 2019; Warren et al. 2020; Mao et al. 2021), allowing the accurate definition of orthologous regions in primates.

In this study, we inspect the recent evolutionary dynamics of splicing programs using high-quality full-length transcriptomes obtained by integrating multispecies deep Iso-Seq and RNA-seq data for a panel of lymphoblastoid cell lines (LCLs) from humans and NHPs (Fig. 1). These full-length transcriptomes substantially expand the current isoform repertoires in primates, and additional proteomic experiments (MS/MS) provide evidence of protein translation from novel transcripts (Fig. 1). We leveraged our transcriptomes to reconstruct transcript expression gains and losses in the primate lineage, linking these expression differences to genetic changes in splice sites and novel exonization events. Innate immune system–related genes and cell proliferation genes are preferential targets of evolutionary innovations. Moreover, the convergent accumulation of these innovations and their occurrence in genes under positive selection in primates highlight the strength of the evolutionary pressures suffered by the immune response and other cell type–specific functions.

**Figure 1.**
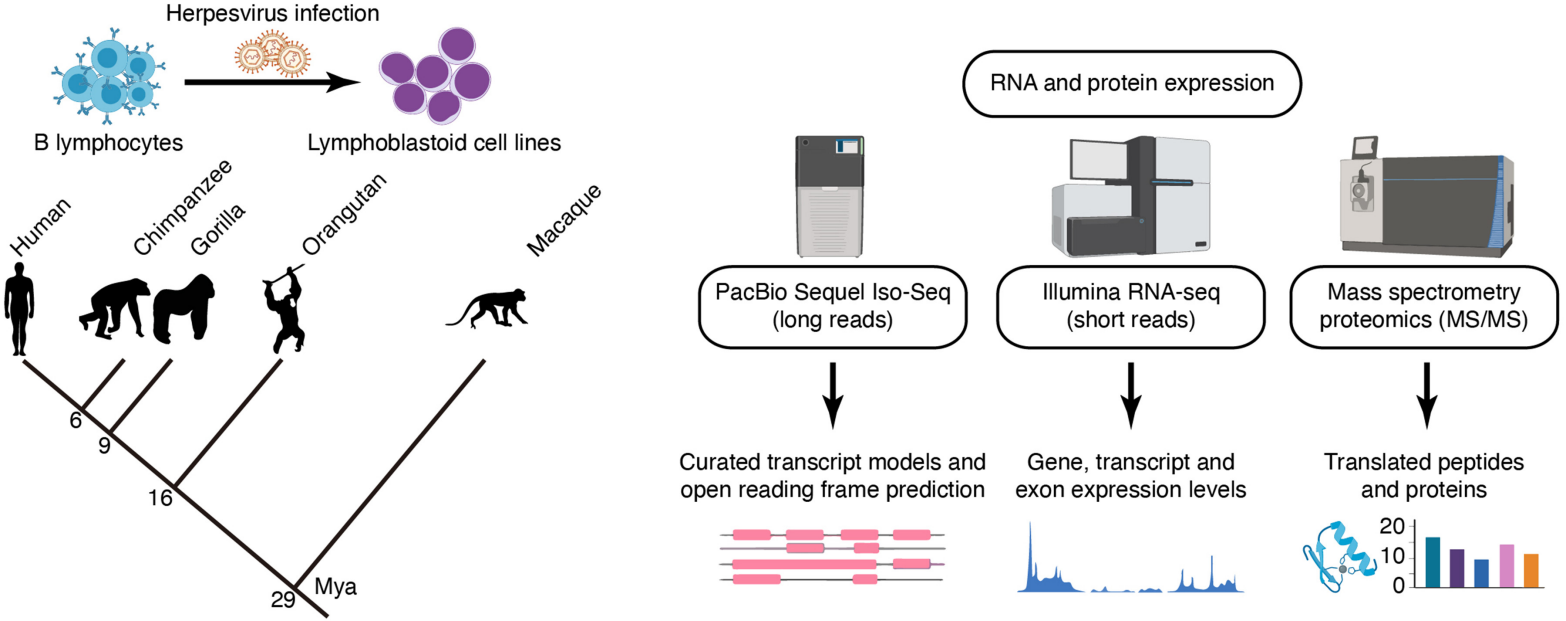
Characterization of transcriptomes and proteomes of human (*Homo sapiens*), chimpanzee (*Pan troglodytes*), gorilla (*Gorilla gorilla*), orangutan (*Pongo pygmaeus*), and rhesus macaque (*Macaca mulatta*) lymphoblastoid cell lines (LCLs). We performed multispecies full-length isoform sequencing (PacBio Iso-Seq), RNA-seq (Illumina), and tandem mass spectrometry experiments (MS/MS). (Mya) Million years ago.

## Results

### Isoform sequencing largely improves human and other primate annotations

To define high-quality transcriptomes, we combined high-depth long reads with short reads in our panel of LCLs derived from humans and NHPs (Supplemental Table S1), resulting in the largest full-length isoform catalog in primates to date (Supplemental Table S2). Briefly, Iso-Seq subreads for two human, two chimpanzee, two gorilla, one (two isogenic cultures) orangutan, and two rhesus macaque LCLs were processed to produce an average of over 2.3 million circular consensus sequences (CCS) per species (range from 1.9 to 2.9 million CCS). A strict quality control, including base correction and splice junction adjustment with RNA-seq, was conducted to refine the transcript models (Methods) (Supplemental Methods). As a result, we defined a total of 148,610 isoforms (on average, 29,722 per species) in 42,814 genes (on average, 8563 genes per species), which were further assessed by SQANTI (for the data production in each processing step, isoform length distributions, and saturation analyses, see Supplemental Tables S3, S4; Supplemental Figs. S1, S2; Tardaguila et al. 2018).

**Figure 2.**
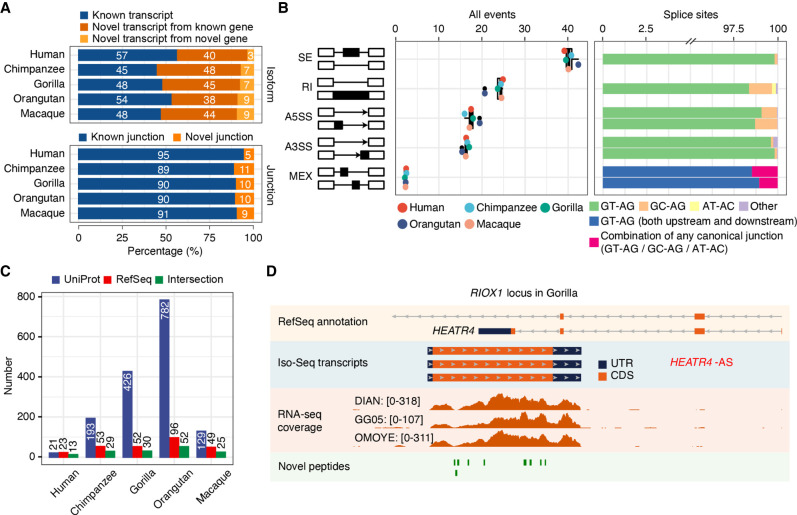
Isoform sequencing and mass spectrometry proteomics largely improve reference annotations. (*A*) Percentage of known versus novel transcripts (*top*) and junctions (*bottom*) captured by Iso-Seq (Ensembl V91). (*B*) Percentage of alternative splicing (AS) events detected by Iso-Seq (*left*) and percentage of splice site combinations associated with each AS class (*right*). (SE) Skipping exons, (RI) retained introns, (A5SS) alternative 5′ splice sites, (A3SS) alternative 3′ splice sites, (MEX) mutually exclusive exons. (*C*) Number of annotated genes with novel detected peptides in each species for UniProt reference proteomes, NCBI RefSeq CDS annotations, or both. UniProt annotations are based on hg38, panTro5, gorGor4, ponAbe2, and rheMac8. NCBI RefSeq CDS annotations are based on hg38, panTro6, gorGor6, ponAbe3, and rheMac10. Peptides mapping to multiple isoforms are included. (*D*) Example of an unannotated gene (*RIOX1 locus* shown in gorGor6 assembly) supported by Iso-Seq, RNA-seq, and mass spectrometry in gorilla samples. [Minimum–maximum] intervals for the number of mapped RNA-seq reads are represented for each gorilla LCL.

Our refined transcriptomes reveal a considerable fraction of novel isoforms (unannotated in each reference transcriptome; 49%, 73,161 out of 148,610) derived from unreported combinations of annotated splice sites, novel splice sites, fusion events, and intergenic transcription (Methods) (Fig. 2A; Supplemental Fig. S3; Supplemental Table S5; Supplemental Data S1; Supplemental Methods). We also captured novel human isoforms (∼19% of our human Iso-Seq transcripts, 9762 out of 50,769) compared with the Universal Human Reference RNA (UHRR) Iso-Seq data set, which comprises full-length isoforms from 10 human cell lines derived from multiple tissues (Supplemental Fig. S4). The AS events newly disclosed by deep long-read sequencing (73% of all detected events, 31,544 out of 43,179) unveil a high level of isoform diversity in primates that is missed by current annotations, especially for NHPs (Supplemental Fig. S5). These events are mostly associated with canonical splice sites (GT-AG, GC-AG, and AT-AC), and thus, they agree with the previous knowledge on RNA splicing mechanisms. Moreover, the contribution of different AS modes is similar across all primate species, and in line with previous observations, exon skipping (SE) and retained introns (RI) are the most prevalent mechanisms (Fig. 2B; Supplemental Table S6; Zhang et al. 2017).

### Mass spectrometry detects missing proteins in NHP reference proteomes

Because most Iso-Seq transcripts are predicted to be protein coding, we performed high-throughput mass spectrometry experiments to search for evidence of peptide translation. To do so, we predicted the ORF of all Iso-Seq transcripts in all species and built a comprehensive protein database that constitutes our search space for peptides across human and NHP LCLs, allowing cross-species peptide identifications for genes detected by Iso-Seq (Supplemental Methods). Within this search space, we detect a total of 49,428 distinct tryptic peptides (FDR < 5% and Mascot ion score > 20, excluding contaminants), from which over 24,900 are confidently detected, on average, in each species (reporter ion intensity signal [abundance] per sample greater than 50 in any sample of a given species, and species-wise median ratio of sample abundance to pool abundance greater than 0.6) (see Supplemental Table S7; Supplemental Data S2; Supplemental Methods). These peptides confidently detected in our mass spectrometry experiments are more frequently annotated in reference proteomes from any species (>99% are annotated, 34,214 out of 34,266 detected peptides) compared with the total set of tryptic peptides predicted from Iso-Seq isoforms (∼88% are annotated, 885,709 out of 1,009,703 predicted peptides).

A number of Iso-Seq-derived peptides detected in each species are novel (i.e., not annotated) in the corresponding UniProt reference proteomes (Supplemental Methods), including 22 for human (hg38), 320 for chimpanzee (panTro5), 882 for gorilla (gorGor4), 1590 for orangutan (ponAbe2), and 207 for rhesus macaque (rheMac8) (for the number of annotated genes with novel detected peptides, see Fig. 2C). To explore these results in the context of the most recent primate assemblies, we then searched the detected peptides in the new CDS annotations (NCBI RefSeq CDS), which provide not only curated proteins (NP) but also in silico predictions (XP). On average, nearly 78% of our detected tryptic peptides in NHPs are only present in predicted entries (XP) from NHP RefSeq proteomes, highlighting the value of our multispecies mass spectrometry experiments in primates to confirm such protein predictions for the first time. When considering both curated (NP) and predicted (XP) proteins, we detected 25 novel peptides in hg38, 98 in panTro6, 94 in gorGor6, 225 in ponAbe3, and 75 in rheMac10 RefSeq CDS sets corresponding to unannotated and annotated genes (Fig. 2C; Supplemental Data S3, S4). Based on these novel peptides, we only observed unannotated protein-coding genes in NHPs, with all their human orthologs being actually annotated in the hg38 gene models. The structural classification of Iso-Seq isoforms from which these novel peptides were predicted and the number of species in which the peptides were simultaneously detected are found in Supplemental Figures S6 and S7, respectively. Our results clearly illustrate the distinct levels of incompleteness in primate reference proteomes, reflecting the differences in the previous efforts made by the community to complete the gene repertoire and study the proteome of each species.

These improvements in proteome annotations provide a vantage point to study the expression of proteins in primates. An example of an unannotated gene with multiple supportive peptides detected in NHP is *RIOX1* (Fig. 2D), which is classified as a pseudogene in Ensembl (gorGor4), and it is absent in RefSeq (gorGor6) despite its importance in cancer research (Bundred et al. 2018). In other cases, we expand the exon and CDS annotations, as in the gene *SON* in orangutan (ponAbe3), a key splicing factor in cell cycle progression involved in the response to the hepatitis B virus (Supplemental Fig. S8; Sun et al. 2001; Ahn et al. 2011; Kim et al. 2016; Tokita et al. 2016). Our results show that integrating cross-species Iso-Seq with mass spectrometry significantly improves proteomic annotations even in the most recent NHP assemblies.

### Emergence of species-specific genomic regions under active transcription

To study the recent transcriptome evolution in primates, we first focused on the genomic presence/absence of the expressed transcripts in the different species. To do so, we inspected orthologous transcripts for the set of isoforms detected in at least one species’ Iso-Seq, as long as they are transcribed from genes with one-to-one orthology in the five primates (Supplemental Fig. S9; Supplemental Methods). The genomic coordinates of 49,797 transcript models (out of 55,888, 89.10%) from 7858 genes were effectively mapped to the five primates’ assemblies to define orthologous transcripts (Supplemental Fig. S10; Supplemental Methods), highlighting the high genomic conservation of transcript structures (Supplemental Data S5, S6). In addition to this high conservation, we found 61 species-specific exons without an orthologous counterpart in the most recent assemblies of the other primates (Methods) (Supplemental Data S7; Supplemental Methods). A significant fraction of them (72%, 44 out of 61) have not been annotated before, which is possibly owing to their lower expression levels (Supplemental Fig. S11).

Compared with conserved exons, these species-specific exons are more frequently located in repetitive regions (79% of them overlap repeat elements, 48 out of 61), particularly SINEs (mainly *Alu* elements) and LINEs (Supplemental Fig. S12), transposable elements that have been previously associated with the birth of new exons (Makałowski et al. 1994; Sorek et al. 2002; Cordaux and Batzer 2009; Schwartz et al. 2009; Avgan et al. 2019). We then wondered if these exonizations might impact the protein function by inspecting the combinations of protein domains encoded in the transcript (Supplemental Methods). In line with previous observations (Corvelo and Eyras 2008), we found that the incorporation of these exons in the transcript usually results in annotated protein domain combinations with likely conserved functions, with only ∼7% of exonization events (four out of 61) deriving in novel protein domain combinations. In most cases (85% of exonizations, 52 out of 61), the new exon is partially (N = 35) or completely (N = 17) included in untranslated regions (UTRs), being significantly depleted in coding sequences compared with conserved exons (Fisher's exact test *P* = 0.02391, OR = 0.51). Thus, our findings are consistent with the previously observed relationship between exon age and coding potential (Zhang and Chasin 2006; Merkin et al. 2015). As an example, in our LCLs, the exonization of three repeat elements in human *MRNIP* results in a longer 3′ UTR with predicted small interfering RNA (siRNA) binding sites (Supplemental Fig. S13).

### Isoform innovations revealed by single-molecule sequencing

**Figure 3.**
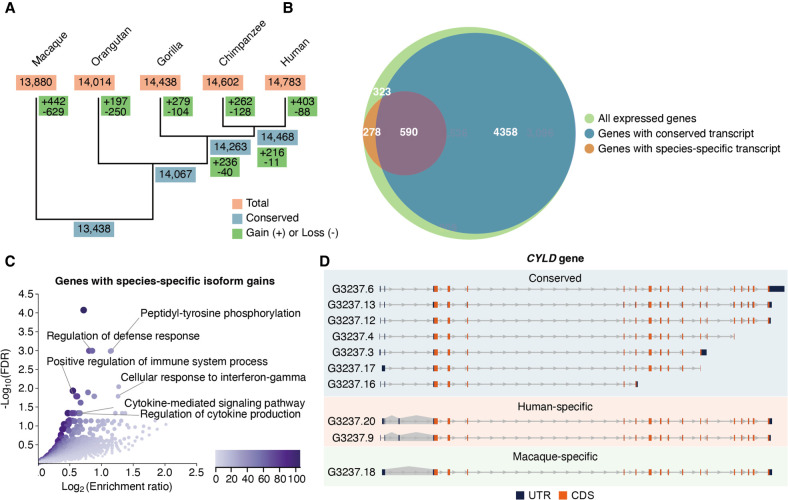
Transcript expression gains and losses in the primate lineage. (*A*) Dynamics of transcript expression gains and losses in five primate species (inferred by Wagner parsimony). The phylogenetic tree is built based on the known phylogeny. Transcript expression gains and losses in macaque versus great apes are shown in macaque's branch, although the direction (gain or loss in macaque or great apes) cannot be determined. (*B*) Overlap of genes expressing highly conserved (blue) and species-specific transcripts (orange). (*C*) Functional enrichment for genes displaying species-specific isoform innovations (N = 868 genes). Overrepresentation analysis (ORA) was conducted using the Gene Ontology Biological Process database (affinity propagation clustering). False-discovery rates (FDRs) were adjusted using the Benjamini–Hochberg method (Benjamini et al. 2001). The color legend indicates the number of overlapping genes between the gene set under evaluation and the pathway gene set. (*D*) Example of a gene, *CYLD*, expressing transcripts in all five species (conserved; blue shadow), exclusive expression in human (human-specific; orange shadow), and exclusive expression in rhesus macaque (macaque-specific; green shadow). Gray shadows indicate species-specific splice junctions. Untranslated regions (UTRs) and coding sequences (CDSs) are colored in blue and orange, respectively.

The high conservation of the gene structures led us to analyze the evolution of transcript expression in primates. We focused on the set of orthologous transcript models in the five genomes, and quantified transcript expression levels using high-depth RNA-seq data from 15 LCLs (three LCLs per species, including the cell lines used to produce long-read transcriptomes) (see Supplemental Table S1) to obtain accurate expression estimates (Methods) (Supplemental Methods; Supplemental Data S8). Consistent with widespread transcriptome conservation, a high number of transcripts are expressed in all five species (N = 18,935 transcripts), whereas 54% (22,198 out of 41,133) of all projected transcripts are only expressed in some species. Thus, to further understand the patterns of transcript expression gains and losses in the primate lineage, we reconstructed the evolutionary dynamics under Wagner parsimony (Csűös 2010). We reasoned that at least part of the transcripts not detected in all species can result from inter-individual transcript expression variability. To account for this variability, we established stringent criteria to keep the transcripts that are consistently expressed or unexpressed based on the RNA-seq from all biological replicates across species (Methods) (Fig. 3A,B; Supplemental Fig. S14; Supplemental Data S9; Supplemental Methods). The number of high-confidence expressed transcripts (H: 14,783; C: 14,602; G: 14,438; O: 14,014; M: 13,880) and the number of species in which they are simultaneously expressed (for an evaluation of transcriptome complexity across the five species, see Supplemental Fig. S15) are balanced across primates, with a high percentage of novel transcript models in each reference transcriptome being also unannotated in the remaining species (63% on average) (Supplemental Fig. S16; Supplemental Methods).

Based on these criteria, we defined 1215 high-confidence species-specific transcripts (only expressed in a single species; H: 332; C: 201; G: 167; O: 128; M: 387 transcripts) produced by genes with one-to-one orthology in the five primates. The vast majority of them (83%, 1009 out of 1215) arise from species-specific splice junctions, with SE being the main mechanism involved in species-specific isoform expression gains (Supplemental Fig. S17). To inspect the usage of our set of species-specific splice junctions (H: 266; C: 156; G: 140; O: 116; M: 337 junctions) across human tissues, we searched RNA-seq junction reads in GTEx samples and found that human-specific junctions are widely used, being supported in a median of 29 human tissues. In contrast, species-specific junctions from NHPs are largely unsupported in human tissues (median = 0 supportive GTEx tissues), suggesting that our results are extensible to other cell types (Supplemental Figs. S18–S20; Supplemental Methods). Furthermore, we confirmed that our detections of species-specific gains are not driven by increased gene expression levels in the corresponding species, because 783 out of 868 genes expressing species-specific transcripts (90.21%) are not significantly up-regulated in the corresponding species (DESeq2 Wald test, Benjamini–Hochberg adjusted *P*-value < 0.1 and log_2_FoldChange > 0; see Supplemental Methods) (for the distribution of total gene expression levels and number of projected transcript models in genes only producing transcripts expressed in all five primates and genes that express species-specific transcripts, see Supplemental Figs. S21, S22). This set of transcripts is enriched in unannotated isoforms (OR = 3.86, Fisher's exact test *P* = 1.4 × 10^−117^), demonstrating the suitability of our strategy to uncover isoform gains otherwise missed by reference transcriptomes (Supplemental Figs. S23, S24).

Importantly, the genes expressing species-specific transcripts show an overrepresentation in innate immune system functions compared with the background of LCLs-expressed genes under evaluation (Methods) (Fig. 3C; Supplemental Table S8) and are more often involved in immune system–related diseases (e.g., *OAS2*, *OAS3*, *RNASEL*, *IRF7*, and *JAK1*; Fisher's exact test *P* = 0.016, OR = 1.50). Given the involvement of these genes in the innate immune response, we then asked if they were enriched in any functional subgroup according to a curated classification of innate immunity genes (Deschamps et al. 2016). In particular, immune sensors (Fisher's exact test *P* = 0.019, OR = 1.96), effectors (*P* = 0.007, OR = 2.5), and secondary receptors (*P* = 0.027, OR = 3.31) are more likely to express species-specific isoforms in any primate species. Furthermore, genes expressing species-specific transcripts in any primate species tend to be highly expressed in the human spleen, which actively participates in the immune response (GTEx expression data across all human tissues, hypergeometric test −log_10_ corrected *P* = 3.14, FC = 1.95; Methods). We also found that these genes producing species-specific isoforms are enriched in genes involved in cell proliferation, consistent with an enrichment in LCL-specific functions (Fisher's exact test *P* = 0.002, OR = 1.61). In contrast, we observed that this set of genes is depleted in housekeeping genes (Fisher's exact test *P* = 5.46 × 10^−6^, OR = 0.64), showing that the essential role of housekeeping genes is also reflected in the conservation of their global splicing structures.

An illustrative example of transcriptomic innovation is *CYLD*, a tumor-suppressor deubiquitinase involved in NF-kB activation (Sun 2010; Mathis et al. 2015), whose splicing patterns impact the regulation of immune cells activation (Hövelmeyer et al. 2007; Srokowski et al. 2009). In LCLs, we observe species-specific expression in *CYLD* isoforms (Fig. 3D; Supplemental Fig. S25). In this case, the inclusion of a noncoding exon generates two human-specific transcripts, whereas an alternative acceptor site produces a macaque-specific transcript. Another example is interferon induced protein 44 like (*IFI44L*), which is involved in the immune response to infection and autoimmune disorders (Herberg et al. 2016; Zhao et al. 2016; de Oyarzabal et al. 2019; Bian et al. 2020; Busse et al. 2020; Hadjadj et al. 2020; Mick et al. 2020; Shaath et al. 2020; Lempainen et al. 2021; Li et al. 2021; Unterman et al. 2022) and whose isoform expression has also been found to be clinically relevant (Feenstra et al. 2014; Haralambieva et al. 2017). *IFI44L* expresses three human-specific transcripts encoding a different ORF than the isoforms expressed in all species (Supplemental Fig. S26). Our results indicate that immune system– and cell proliferation–related genes are preferentially targeted by recent transcriptomic species-specific innovations within the 7858 orthologous genes expressed in LCLs.

### The impact of *cis*-regulatory genetic changes in isoform innovation

The efficient inclusion of exons into mature transcripts is mainly determined by splicing signals located in the exon boundaries. Thus, interspecies genetic changes affecting the orthologous splice sites might contribute to events of transcript diversification, including the emergence of species-specific isoforms. In the context of our transcriptomes, we examined the sequence conservation of orthologous splice sites (terminal intron dinucleotides). Overall, we observed widespread sequence conservation of splice sites across primates, reflecting strong selective constraints in these regions (Supplemental Fig. S27). To explore high-impact interspecies changes in splice sites, we then classified all orthologous junctions into canonical and noncanonical, associated with high and low splicing efficiencies, respectively. After filtering out lowly expressed isoforms and genes (Supplemental Methods), we observed 82,527 splice junctions present in our transcript models, from which 1318 show a change in canonicity in any species compared with their orthologous positions. This scenario affects 16% of genes (N = 1008 genes), which are enriched in T cell (FDR = 0.019) and B cell (FDR = 0.019) activation as well as JAK/STAT signaling (FDR = 0.022) pathways compared with the background of genes under evaluation (Methods) (Supplemental Table S9), showing that *cis*-regulatory changes in orthologous splice sites are primarily associated with genes involved in immune system functions.

We then investigated the contribution of these genetic changes to the expression of species-specific isoforms. We observed that 69% of the high-confidence species-specific isoforms (833 out of 1215 isoforms) harbor conserved canonicity of their splice sites in all species. In contrast, the expression of 184 species-specific transcripts is driven by genetic differences producing canonical splice sites only in the species in which they are expressed. The enrichment in immune system functions holds for species-specific transcripts regardless of if they occur with highly conserved orthologous splice sites or not, showing the global relevance of recent transcriptome changes in the evolution of the immune system (Supplemental Fig. S28).

An example of interspecies genetic differences in the splice sites is *RRP15*, a gene involved in cell proliferation and apoptosis (Wu et al. 2016). In *RRP15*, a high-frequency variant only present in modern humans (Kuhlwilm and Boeckx 2019) disrupts an acceptor site of a coding exon (from AG to TG), and a downstream splice site is used instead. This mutation results in a human-specific exon shortening representing a deletion of three amino acids (disordered residues involved in protein binding), whereas the reading frame is preserved compared with other primates (Supplemental Fig. S29; Zhang and Kurgan 2019; Zhao et al. 2021). In other scenarios, the genetic change in splice sites promotes the transcription of a new exon, as we observe in gorilla LCLs for *NAP1L4* gene (Supplemental Fig. S30).

### Evolutionary dynamics of isoform usage patterns in the primate lineage

**Figure 4.**
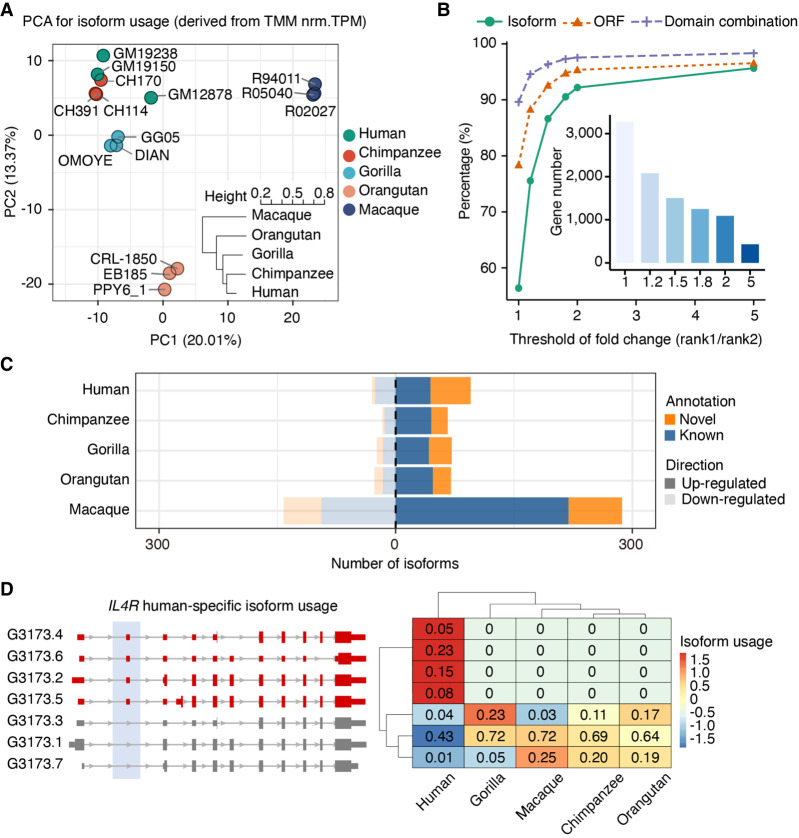
Isoform usage (IU) dynamics across primates. (*A*) Principal component analysis (PCA) for isoform fraction values of 43,484 transcripts from one-to-one orthologous genes. A hierarchical clustering for the Euclidean distances of IU Spearman's correlations across samples is also shown. IU values were calculated from transcripts per million (TPM) after batch effect correction (comBat) and trimmed mean of *M*-values (TMM) normalization (Supplemental Methods). (*B*) Percentage of rank 1 isoform (green), ORF (orange), and protein domain combination (purple) shared by all primates by different thresholds of expression fold change between rank 1 and rank 2 isoforms. The number of genes considered for each threshold is indicated in the inner bar plot. (*C*) Number of isoforms showing species-specific usage changes. Up-regulated (dark shadow) and down-regulated (light shadow) isoforms are classified into novel (orange) and known (blue) isoforms based on the reference transcriptome in the corresponding species (NHP novel assemblies). (*D*) *IL4R* human-specific IU. Human-specific up-regulated isoforms are colored in red; nondifferentially used isoforms, in gray (*left*). IU values across species are shown in the heatmap (*right*), where colors represent row-scaled IU values (legend).

After studying the evolution of switching on/off isoform transcription, we aimed to investigate the evolution of isoform transcription levels. To disentangle isoform-specific transcription regulation from changes in total gene expression, we assessed the relative transcript usage across the five species. To do so, we computed sample-wise fractions of isoform usage (IU) in our quantified orthologous transcripts (Supplemental Methods) (for the distribution of IU values across LCLs and species, see Supplemental Fig. S15). Principal component analysis and hierarchical clustering indicate that IU values cluster by species and that differences across species increase with evolutionary distance, reflecting the known phylogeny (Fig. 4A; Supplemental Methods). As expected, IU values are higher in annotated transcripts than in novel isoforms (Wilcoxon rank-sum test *P* < 2.2 × 10^−308^), indicating that isoforms displaying important contributions to the expression of their genes are better represented in reference annotations (also see Supplemental Figs. S31, S32).

To investigate the usage conservation of transcripts with a major contribution to the total gene abundances, we then focused on the isoforms with the highest IU across primates (rank 1 and rank 2 transcripts) after filtering out genes with very similar transcripts (internal exon extensions leading to quantification ambiguities) (see Supplemental Methods). Because different isoforms can produce identical proteins, we also evaluated the conservation of the ORF and of the combination of protein domains encoded by the dominant transcripts (rank 1) in different species. We found that all species share the same dominant transcript for 55.44% of the genes (1814 out of 3272), a trend that is more evident as bigger fold changes of expression between rank 1 and rank 2 transcripts are considered (Fig. 4B), suggesting that changes in the dominant transcript are less common. This conservation is higher for the protein-coding features, with 78.33% and 90.01% of genes sharing identical ORF or combinations of protein domains in their dominant transcripts in all species, respectively, consistent with changes in the dominant transcript being almost always functionally conservative. Our results highlight the greater constraints associated with protein structure and function conservation, whereas distinct preferences in isoform UTR or even subtle ORF changes are more frequent in the dominant transcripts across the primate phylogeny.

We investigated other changes of relative transcript usage across primates. To do so, we conducted pairwise comparisons to find interspecies differential isoform usage (DIU) (Supplemental Methods) after excluding lowly expressed genes and isoforms (Soneson et al. 2016), as well as genes expressing highly similar transcripts (variability in internal exon boundaries) (Supplemental Methods). In a scenario of broadly conserved IU across species, we detected over 800 transcripts showing species-specific usage changes (Supplemental Data S10; for a comparison with a less conservative strategy to retrieve species-specific IU changes, see Supplemental Fig. S33), a significant fraction of which are missed by reference annotations (Fig. 4C; Supplemental Fig. S34). LCL-specific genes display species-specific DIU changes more frequently (Fisher's exact test *P* = 0.015, OR = 1.69), although we did not observe any pathway enrichment for the genes displaying species-specific DIU when considering all evaluated genes as the background (Methods). An example of human-specific DIU is the interleukin 4 receptor (*IL4R*), a gene under positive selection in primates (van der Lee et al. 2017), which plays a major role in the immune response (Nelms et al. 1999). We detected four up-regulated *IL4R* transcripts in human LCLs, two of them encoding shorter *IL4R* proteins (Fig. 4D).

### Local differential splicing dynamics in transcribed regions

**Figure 5.**
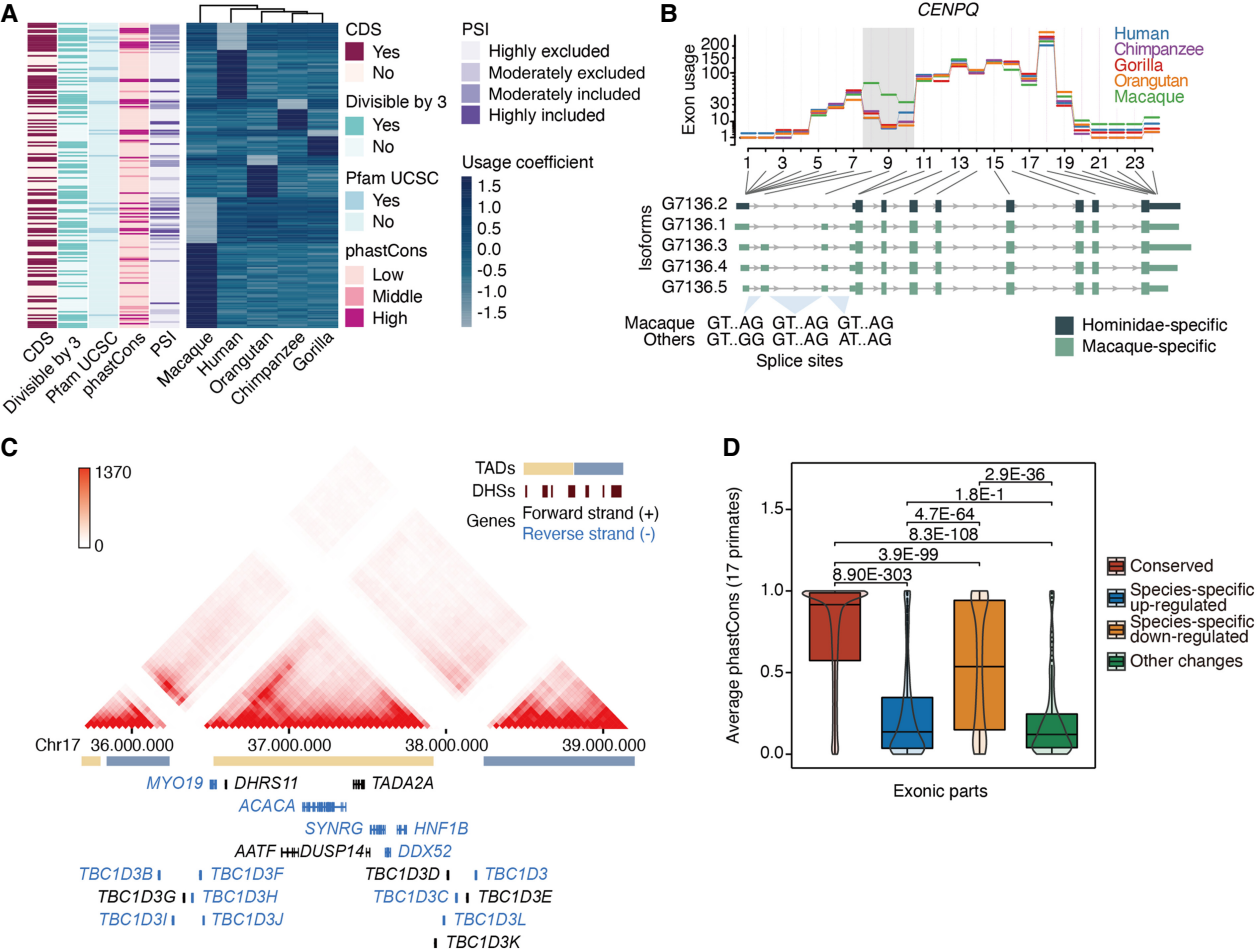
Exon usage dynamics across primates. (*A*) Usage coefficients for skipping exons displaying species-specific changes (row-wise scaled). Overlap with coding sequence (CDS) and Pfam protein domains (UCSC), divisibility by three, average percentage-splice-in (PSI), and average phastCons scores are shown for each exon (see Supplemental Methods). (*B*) Example of gene (*CENPQ*) with different splice sites in macaque and great apes. Exon usage coefficients are illustrated at the *top*. Hominidae- and macaque-specific Iso-Seq transcripts are color-coded (*bottom*). (*C*) Chr17q12 region with high density of human-specific DEU. Hi-C interaction intensity is indicated in the color legend. Gene orientation, DNase hypersensitive sites (DHSs), and topologically associating domains (TADs) are also displayed. (*D*) Average conservation score in primates for exonic parts with conserved usage (N = 95,453), species-specific up-regulation (N = 941) or down-regulation (N = 1340), and exonic parts showing other usage changes (N = 277). “Other changes” correspond to usage differences between groups of species (e.g., any two species show differential usage vs. the remaining three species). Exonic parts longer than five bases (all included as passed in 1000 Genomes hg38 strict mask) are shown. Statistical significance of the difference across groups was assessed by the Wilcoxon test, and shown *P*-values are adjusted by the Holm method.

Although full-length isoform expression levels provide an accurate view of major usage changes in clearly different transcripts, other local splicing changes are better detected by total exon expression levels that accumulate the signal of all transcripts including the exon. We assessed the differential exon usage (DEU) across the primate phylogeny to obtain a comprehensive view of isoform transcription evolution (Supplemental Methods). We first leveraged our projected Iso-Seq models to define 201,191 orthologous exonic parts (i.e., transcribed regions) from 7766 one-to-one orthologous genes. Then, RNA-seq read counts in these exonic parts were obtained across all samples to detect interspecies differences in relative exon usage (Supplemental Methods). We observed nearly 4000 exonic parts displaying species-specific local usage changes (Supplemental Data S11), with skipping exons (SE) being a prevalent AS event among them (for a description of features in SE showing species-specific usage changes, see Fig. 5A). As in the species-specific exonic structures (see section “Emergence of species-specific genomic regions under active transcription”), we observed significant enrichment of *Alu* elements in human-specific up-regulated exonic parts compared with down-regulated or regions of conserved usage (Supplemental Fig. S35). This suggests that *Alu* insertions contribute to increasing orthologous exon inclusion frequencies, especially for the case of SE. This is consistent with previous reports on the *Alu*-mediated modulation of exon usage rates (Payer et al. 2019).

We found that genes with species-specific DEU are enriched in functions related to the regulation of immunity, such as interferon signaling and innate immune response (*IRF2*, *IRF3*, *IRF5*, *IRF7*, *IRF9*, *IFI35*, *IFIT1*, *ISG20*, *IFNGR2*, *HLA-DQB1*, *CIITA*, etc.), as well as proliferative (DNA repair, centrosome localization, etc.), housekeeping (ncRNA processing, mitochondrial gene expression, etc.), and viral life-cycle control functions compared with a background set consisting of all genes evaluated in DEU analyses (FDR ≤ 0.05; Methods) (Supplemental Table S10). An example of this is *CENPQ*, a centromere protein involved in autoimmune disease and mitosis progression (Song et al. 2013; Bancroft et al. 2015), whose species-specific DEU is driven by a genetic change in the primate splice sites. In macaque, two junctions harbor GT–AG splice sites, leading to the recognition of two 5′ UTR exons and the preferred usage of the isoforms that include them. On the contrary, great apes, in which these splice sites are not canonical, only express the transcript that skips these exons (Fig. 5B). We also noticed that 13.89% of genes displaying species-specific DEU (221 out of 1591) co-occur with gene expression up- or down-regulation in the same species. Six genes down-regulated in human LCLs (*SYNRG*, *DUSP14*, *TADA2A*, *ACACA*, *MYO19,* and *DDX52*) are comprised in the same topologically associating domain in Chr17q12 (Rao et al. 2015) and display human-specific exon usage changes (Fig. 5C). Genomic rearrangements in this locus have been extensively studied given their evolutionary relevance and implications in many diseases, including neurodevelopmental disorders (Mefford et al. 2007; Cardone et al. 2008; Moreno-De-Luca et al. 2010; Nagamani et al. 2010). To date, this is the first report of human-specific splicing changes occurring in multiple genes encoded in this region.

We found that exonic parts experiencing species-specific usage up-regulation display lower sequence conservation in primates than exonic parts down-regulated in a given species (Fig. 5D). This higher variation in species-specific up-regulated regions is also reflected in the nucleotide diversity within human populations (Supplemental Methods; Supplemental Fig. S36). Furthermore, when focusing on transcribed regions with human-specific usage changes, we noticed up-regulated exonic parts are depleted in CDSs (Supplemental Fig. S37) and are less present in principal isoforms (defined by APPRIS) (Rodriguez et al. 2018) compared with human-specific down-regulated or regions of conserved usage (Supplemental Fig. S38). Taken together, these findings suggest that species-specific gains in exon usage are associated with fast-evolving regions whose changes in usage lead to subtle or regulatory changes in the main function of these genes.

We reasoned that genes with splicing changes detected across primates might show characteristic selective pressures in their coding sequences. To investigate this possibility, we assessed the ratios of nonsynonymous to synonymous substitutions (*d*_N_/*d*_S_) in human populations for the genes under evaluation (van der Lee et al. 2017). To do so, we combined our results from isoform gains and losses and DEU and DIU analyses to define gene classes according to their splicing patterns: those showing any species-specific up-regulation signal in humans (N = 278 genes) or NHPs (N = 1239 genes), those displaying multiple events of species-specific up-regulation in several species (N = 287 genes), those experiencing other splicing changes consistent with differences between groups of species (N = 1174 genes), and genes with fully conserved splicing patterns (N = 4532 genes) (Supplemental Methods). We observed significantly higher *d*_N_/*d*_S_ in human populations for the gene sets showing splicing changes involving NHPs than for genes with fully conserved splicing, whereas the genes with only human-specific up-regulation show intermediate *d*_N_/*d*_S_ (Supplemental Fig. S39). These results suggest a link between dynamic splicing evolution in primates and accelerated or less constrained coding changes in human populations, with intermediate selection constraints in genes with only human-specific up-regulation.

To further investigate the occurrence of interspecies splicing changes in genes under adaptive selection, we then asked if the above-defined gene classes showing splicing changes were enriched in signals of positive selection in primates (van der Lee et al. 2017) in comparison to genes with conserved splicing patterns. We found that only genes showing independent events of species-specific up-regulation in multiple primates are significantly enriched in signals of positive selection (Fisher's exact test, Bonferroni-adjusted *P* = 0.014, OR = 2.90), indicating that they might be important targets of adaptive evolution. This set of genes accumulating recurrent species-specific changes in the splicing programs of different primates is enriched in genes associated with the regulation of the immune response (Gene Ontology Biological Process overrepresentation analysis [ORA], FDR = 0.03, enrichment ratio = 2.17), reinforcing the link between evolutionary changes in splicing and the immune function.

## Discussion

To date, our knowledge of the evolution of primate transcriptomes is very limited. Here, we have generated the most extensive catalog of full-length isoforms across the primate lineage complemented with matching Illumina RNA-seq data. The combination of both allowed us to create highly curated transcriptomes consisting of a high proportion of novel transcripts from annotated genes, as well as a number of antisense and intergenic transcripts. Furthermore, new AS events have been identified, and we have observed a conserved composition of AS classes in great apes and rhesus macaque. This resource greatly expands the available gene annotations for model and nonmodel primates by providing a more accurate and complete view of genes and isoforms, which are crucial for further genomic and functional studies.

In this study, we have taken advantage of our curated isoform catalog to predict the corresponding ORF for coding transcripts, representing the majority of the sequenced transcriptomes. This ORF collection has been fundamental to build proteomic databases assisting coupled mass spectrometry analysis. In this way, we detected evidence of translation for Iso-Seq transcripts identifying peptides that are not present in reference proteomes. We detected fewer novelties at the proteome than at the transcriptome level, reflecting a combination of the differences in experimental sensitivities, in the completeness of proteome and transcriptome annotations, and in the proteome and transcriptome complexities (Ezkurdia et al. 2015). All considered, our analyses show that there is still room for improvement of proteome annotations by integrating isoform resolution sequencing data.

From an evolutionary point of view, many immunity-related genes have undergone strong selective pressures in response to constantly evolving host–pathogen interactions, leading to the accelerated evolution of these genes (Barreiro and Quintana-Murci 2010; Barreiro et al. 2010). However, very few studies have addressed how these rapid evolutionary rates are reflected in the immune system–related splicing programs in the context of recent human evolution (Sams et al. 2016; Rotival et al. 2019). LCLs display transcriptional profiles that are very similar to those of plasmablasts and early plasma cells (Mrozek-Gorska et al. 2019), allowing us to study the transcription of immune system–related genes. The full-length isoform characterization of our multispecies LCLs panel showed a significant number of isoform gains and losses in a context of widespread transcript expression conservation. We found that genes involved in the regulation of innate immunity are enriched in species-specific isoforms even when controlling for the composition of LCLs transcriptomes. For example, interferon-response genes, which show early expression divergence in activated leukocytes from different primates (Hawash et al. 2021), often produce species-specific isoforms in primate LCLs. This is also true for cytokine and chemokine genes, which display major transcriptional differences in stimulated fibroblasts and phagocytes from different mammals (Hagai et al. 2018). The concurrence of global gene expression and splicing differences across closely related species underlines the fast, multilayered transcriptome evolution of genes involved in the immune response.

We also detected high-impact genetic changes located in orthologous splice sites, which are clearly involved in shaping the splicing preferences of immune system–related genes in different primate species. Particularly, SE events are enriched in species-specific transcript gains, posing this type of AS as a prevalent mechanism in the recent evolution of primate transcriptomes. Taking advantage of the most recent NHP genome assemblies, we have also characterized the emergence of new exons, many of them absent in reference annotations, and confirmed the association between the exonization process and the insertion of transposable elements such as SINEs and LINEs (Makałowski et al. 1994; Sorek et al. 2002; Cordaux and Batzer 2009; Schwartz et al. 2009; Avgan et al. 2019).

The patterns of IU recapitulate the phylogenetic structure in primates, with the most abundant transcripts of each gene frequently encoding the same ORF or showing conservative changes across species. This highlights that recent isoform changes usually preserve the functional integrity of the protein encoded by the gene, representing a source of subtle regulatory fine-tuning for important genes. Here, we report a number of interesting species-specific changes in isoform preferences, involving a significant fraction of isoforms absent in reference transcriptomes. Further analyses will be required to understand the functional implications of the splicing changes affecting coding and noncoding regions. In this direction, it is remarkable that our set of differentially used exons is enriched in UTRs, which harbor binding motifs for RNA-binding proteins and small RNA molecules (e.g., miRNAs), playing fundamental roles in the regulation of isoform stability and localization (Mayr 2019).

The enrichment in signals of positive selection in the coding sequences of genes accumulating independent species-specific gains of isoform expression, IU, or exon usage in multiple primates suggests a link between recurrent splicing changes and adaptive evolution in primates. The immune response is highly dynamic, and global gene expression differences have been previously reported between leukocytes of apes and African and Asian monkeys (Hawash et al. 2021). These changes have also been seen when comparing human, chimpanzee, and rhesus macaque primary monocytes (Barreiro et al. 2010) across multiple time points after stimulation. Considering the functional specialization and dynamism of immune cells, characterizing interspecies isoform expression changes in different immune cell types and multiple time points after infection will be required to further understand this relationship. In the same vein, comparative analyses based on isoform sequencing in other tissues and cell types will help to dissect changes in splicing preferences in primate-accelerated genes that are not expressed in LCLs, such as those involved in nervous system function and neurodevelopment (Dorus et al. 2004). The evaluation of interspecies splicing differences across developmental stages in primates is particularly interesting, because fast-evolving regions in apes have been shown to locate nearby regulators of embryonic development (Kostka et al. 2018).

The Iso-Seq transcriptomes produced here are limited to a single cell type and two biological replicates per species. In this regard, we foresee that comprehensive studies based on long reads will further extend our knowledge on the transcriptome complexity by assessing multiple tissues from numerous species and individuals. The increasing availability of long-read transcriptomes will enable the identification of tissue-specific and cell type–specific isoforms, their inter-individual variability, and evolutionary conservation. In our study, we addressed the unbalanced number of high-quality isoforms captured per species by leveraging deep short-read RNA-seq data (three biological replicates per species) to perform differential expression and usage analyses based on our full-length transcript models, revealing a similar number of expressed transcripts in all five species. However, the lack of saturation in Iso-Seq isoform discovery rates implies that a number of species-specific transcripts might be missed from our transcriptomes. Future advances in sequencing platforms will allow direct comparisons of saturated long-read transcriptomes, which will be essential for the detection of interspecies differences affecting low-abundance transcripts that are hard to infer from short-read RNA-seq alone.

In summary, this study expands our current knowledge on transcript and protein diversity in humans and NHPs, providing a comprehensive evaluation of the dynamics of transcriptome evolution in the primate lineage as reflected in B cell–derived LCLs. The combination of high-depth long- and short-read transcriptomics and high-throughput mass spectrometry provides an excellent framework to characterize many evolutionary novelties missed by reference annotations. The discovery of these recent changes highlights the prevalence of splicing evolution in primates. Our results show that genes involved in the innate immune response, in cell proliferation, and with cell type–specific expression profiles have been targets of numerous isoform changes. The strong adaptive demands of the immune response and cell proliferation at the transcriptional level are satisfied mostly by incorporating fast regulatory and subtle innovations without drastically impacting the viability of the response. Moreover, this set of evolutionary changes constitutes the basis for future functional assays for studying their role in interspecies phenotypic divergences in the immune response and cell proliferation, areas of major interest in evolutionary and biomedical research.

## Methods

### LCLs origin and culture

LCLs were kindly provided by Dr. Antoine Blancher (chimpanzee LCLs CH507, CH170, and CH322), Dr. Aurora Ruiz (gorilla LCL GG05), Dr. Chris Tyler-Smith (orangutan LCL CRL-1850), and Dr. Gaby Dioxiadis (macaque LCL R94011). GM19238 (human) and EB185(JC) (orangutan) were purchased from Coriell Institute and Sigma-Aldrich, respectively. The remaining cell lines used in this article are described by García-Pérez et al. (2021). Detailed information for the panel of LCLs is provided in Supplemental Table S1.

Cells were cultured in RMPI-1640 with L-Glu (Gibco) supplemented with 15% FBS (Gibco) and 1% P/S (10000 U/mL; Gibco). Multiple cell pellets totaling 12 × 10^6^ cells per cell line were stored at −80°C until further extraction after two washes with DPBS.

### RNA extraction

A pellet consisting of 2 × 10^6^ cells was washed once and resuspended in DPBS to use as starting material to extract DNA using the DNeasy blood and tissue kit (Qiagen) following the manufacturer's instructions. RNA was extracted from a 3 × 10^6^ cell pellet, previously stored in QIAzol lysis reagent, using the miRNeasy mini kit (Qiagen) and following the manufacturer's instructions.

### RNA-seq data production and processing

Illumina TruSeq stranded RNA-seq experiments were conducted at Macrogen for the following LCLs: GG05, CRL-1850, GM19238, CH507, CH170, CH322, EB185, and R94011, whereas the RNA-seq for the remaining LCLs (except for GM12878) was generated by García-Pérez et al. (2021). RNA-seq data from GM12878 were retrieved from the NCBI Sequence Read Archive (SRA; https://www.ncbi.nlm.nih.gov/sra) under accession numbers SRR998197 and SRR998198. RNA-seq reads were filtered with SOAPnuke (version 1.5.6, parameters: -Q 2 -l 20 -q 0.5 -3 -E 60 -5 1) (Chen et al. 2018) after adapter removal. Specifically, 3′ adapter sequence of fastq1 file (-f in SOAPnuke) was AGATCGGAAGAGCACACGTCTGAACTCCAGTCAC, and 5′ adapter sequence of fastq2 file (-r) was AGATCGGAAGAGCGTCGTGTAGGGAAAGAGTGTAGATCTCGGTGGTCGCCGTATCATT. Filtered reads were then aligned to each primate genome with STAR (version 2.7.0a, parameters: ‐‐readStrand Reverse ‐‐quantMode TranscriptomeSAM ‐‐outSAMtype BAM SortedByCoordinate) (Dobin et al. 2013).

### Isoform sequencing data production

Two LCLs per species were used for isoform sequencing experiments (PacBio Iso-Seq; human: GM12878, GM19150; chimpanzee: CH114, CH391; gorilla: DIAN, OMOYE; orangutan: two isogenic cultures of PPY6; macaque: R05040, R02027). In the first run, three libraries of 1–2 kb, 2–3 kb, and 3–6 kb with poly(A) enrichment (Oligo(dT)) were generated using SMRTbell template prep kits and sequenced on a Pacific Biosciences (PacBio) sequel system at Novogene. A total of 26 Gb (subread data) was generated per species, with an average length and N50 of 1980 bp and 2399 bp, respectively. In a second run, poly(A)-enriched libraries (Oligo(dT)) were produced without size selection using SMRTbell template prep kits and sequenced on a PacBio sequel system. In this run, a total of 56 Gb (subread data) was generated per species, with an average length and N50 of 2162 bp and 2647 bp, respectively.

### Iso-Seq data processing

SMRT Link (version 6.0) was used to generate full-length polished transcript sequences from PacBio data by combining subreads from the two LCLs per species. These transcript sequences were corrected with Illumina RNA-seq data using LoRDEC (Salmela and Rivals 2014). GMAP (Wu and Watanabe 2005) was used to map the sequences to the corresponding reference genomes after poly(A) trimming. TranscriptClean (Wyman and Mortazavi 2019) was used to refine transcript splice junctions and reduce errors derived from Iso-Seq. Cupcake ToFU scripts were used to remove redundancy from high-quality, uniquely mapped transcript sequences. Nonredundant transcripts were provided to SQANTI for quality control and artifact filtering. Detailed information on Iso-Seq data processing and artifact filtering is described in the Supplemental Methods. A comparison of our pipeline with an alternative strategy involving independent processing of Iso-Seq subreads from each LCL can be found in Supplemental Figures S40 through S42.

### Protein extraction and precipitation

Two million cell pellets were thawed on ice, washed within 1 mL of cold DPBS, and further centrifuged at 2500*g* 5 min at 4°C. After removing the supernatants, 450 µL of cold RIPA buffer (Thermo Fisher Scientific) supplemented with 10 µL/mL Halt protease and phosphatase inhibitor cocktail (100; Thermo Fisher Scientific) was added to disrupt cell pellets. After incubating 5 min with the tubes swirling on ice, the suspension was sonicated for 30 sec with 50% pulse a total of three times with 10 sec of rest using the Branson 250 sonicator. Later, tubes were further incubated for 30 min on ice swirling. Finally, tubes were centrifuged at 14,000*g* for 15 min at 4°C to pellet the cell debris, and supernatants were transferred into new protein LoBind tubes (Eppendorf). Extracted proteins were quantified using a Pierce BCA protein assay kit (Thermo Fisher Scientific) and stored at −80°C until further analysis.

To precipitate the proteins, six volumes of overnight chilled acetone (−20°C) were added, and then tubes were inverted three times and incubated overnight at −20°C. The next day, tubes were centrifuged at 16,000*g* for 10 min at 4°C, and acetone was removed carefully without disturbing the protein pellet.

### Mass spectrometry sample preparation

Three biological replicates for each species were processed. Samples (12–120 µg) were solubilized in 100 mM triethylammonium bicarbonate (TEAB) at a concentration of 1 µg/µL, reduced with tris(2-carboxyethyl)phosphine (0.12–1.20 µmol, 60 min, 37°C), and alkylated in the dark with iodoacetamide (0.23–2.25 µmol, 30 min, 25°C). The resulting protein extract was first diluted to 2 M urea with 200 mM ammonium bicarbonate for digestion with endoproteinase LysC (1:10 w:w, o/n, 37°C; Wako 129-02541) and then diluted twofold with 200 mM ammonium bicarbonate for trypsin digestion (1:10 w:w, 8 h, 37°C; Promega V5113).

After digestion, the peptide mixes were acidified with trifluoroacetic acid (TFA) to have a final concentration of 1% TFA, desalted with a MicroSpin C18 column (The Nest Group), and dried by vacuum centrifugation. Peptide mixes were also solubilized with 100 mM TEAB at a concentration of 1 µg/µL.

Samples were labeled to generate three different TMT mixes; in each TMT mix, one sample of each species was included with a common pool consisting of a mix of all the samples. Labels were used in a way that, in each TMT mix, each species uses a different TMT reagent (see Supplemental Table S11). TMT-6 label reagents were resuspended adding 41 µL of anhydrous acetonitrile (ACN) to each tube. Two microliters of the resuspended TMT label reagent was added to 10 µg of each sample. Additionally, 10 µL of anhydrous ACN and 8 µL of TEAB 100 mM were added to achieve approximately a final concentration of 30% of acetonitrile. Tubes were incubated for 1 h at room temperature, and then 2 µL of 5% hydroxylamine was added and incubated to quench the reaction (15 min, 25°C). Samples were combined in three mixes as shown in Supplemental Table S11, desalted with a MicroSpin C18 column (The Nest Group), and dried by vacuum centrifugation.

TMT mixes were fractionated using basic pH reversed-phase fractionation (Isasa et al. 2015) in an Agilent 1200 system. Peptides were separated in a 90-min linear gradient from 5% to 90% acetonitrile in 10 mM ammonium bicarbonate (pH 8) at a 0.8 mL/min flow rate over an Agilent Zorbax Extend-C18 (4.6 mm ID, 220 mm in length, 5-m particles, 80 Å pore size). Fractions were collected every minute into a total of 96 fractions, which were consolidated into 24, of which only 12 nonadjacent samples were analyzed. Fraction volume was reduced to 300 µL approximately and acidified by adding formic acid up to 10% of the final volume. Fractions were desalted with a MicroSpin C18 column (The Nest Group) and dried by vacuum centrifugation.

### Species-specific exon gains (genomic structure)

LiftOver and Liftoff (Shumate and Salzberg 2020) were used to identify conserved and species-specific exons at sequence level (Iso-Seq exons derived from one-to-one orthologous genes). An exon was defined as conserved when successfully mapped from NHP to human and then to all four NHPs by liftOver. An exon was defined as species-specific when NHP exons failed to be mapped to the human reference genome or human exons failed to be mapped to all four NHPs by liftOver and achieved <50% Liftoff alignment coverage in the remaining four assemblies. Additional information can be found in the Supplemental Methods.

### Transcript expression calculation and reconstruction of transcript gains and losses

RNA-seq of the corresponding species was used to quantify the projected transcript models in each genome by kallisto (‐‐rf-stranded argument, default settings) (Bray et al. 2016). After filtering orthologous transcripts whose splicing structure is not supported by RNA-seq and mono-exonic transcripts, Count (Csűös 2010) was used to reconstruct the tree of transcript expression gains and losses in primates considering the known phylogenetic structure and the resulting binary matrix of transcript expression across species. Two data sets were generated with different criteria: In the less conservative criteria, a transcript was defined as expressed in each species if TPM > 0 in at least two LCLs, whereas the strict criteria required TPM > 0 in all LCLs from the same species (Supplemental Methods). Results of isoform expression across LCLs and functional enrichment of genes expressing species-specific transcripts using TPM = 0.1 as an expression cutoff are shown in Supplemental Figures S43 and S44. Splice junction usage and isoform expression conservation estimated from Iso-Seq or RNA-seq are illustrated in Supplemental Figure S45. Isoform expression conservation was also analyzed in the context of independent Iso-Seq processing for each LCL (without combining subreads from each species) (Supplemental Fig. S46). Principal component analysis of transcript TPM values before and after batch effect correction (Supplemental Methods) is depicted in Supplemental Figure S47.

### *d*_N_/*d*_S_ in human populations and positive selection in primates

Precomputed ratios of nonsynonymous to synonymous substitutions (*d*_N_/*d*_S_) in human populations and genes undergoing positive selection in the primate lineage were retrieved from van der Lee et al. (2017). Briefly, *d*_N_/*d*_S_ ratios were calculated using human variation data from 60,706 exomes collected by the Exome Aggregation Consortium (ExAc, release 0.3.1) (Lek et al. 2016), and positively selected genes were obtained through multiple alignments of orthologous protein sequences in nine primate species (human, chimpanzee, gorilla, orangutan, gibbon, macaque, baboon, vervet, and marmoset). Comparisons across gene classes (see Supplemental Methods, “Classification of genes according to their splicing and usage patterns”) were restricted to genes with a precomputed *d*_N_/*d*_S_ and to background genes for positive selection analysis from van der Lee et al. (2017).

### Functional enrichment analysis

Functional enrichment was performed using ORA (minimum number of IDs in the category = 10, maximum number of IDs in the category = 500, BH FDR method, significance level FDR ≤ 0.05) in WebGestalt (Liao et al. 2019), based on Gene Ontology Biological Processes (The Gene Ontology Consortium et al. 2000; The Gene Ontology Consortium 2021) and PANTHER pathways (human data) (Mi et al. 2021). For each functional enrichment, we adjusted the background gene set to the orthologous genes expressed in LCLs (detected by Iso-Seq) that were under evaluation (background gene sets are different for species-specific expression gains, DIU, and DEU; see the different filtering criteria used in each analysis in Supplemental Methods). In addition, tissue-specific expression of genes producing species-specific transcripts was tested using tissueEnrich (Jain and Tuteja 2019) against GTEx RNA-seq expression data in 29 human tissues (The GTEx Consortium 2015) with default settings (significance-level BH-adjusted *P*-value < 0.01). Background gene set was restricted to the list of genes under evaluation in isoform expression gains/losses analysis.

We tested the enrichment and depletion in particular gene lists related to the innate immune response, immune disease, LCL-specific expression, and housekeeping genes. A manually curated classification of innate immunity genes (eight subgroups: accessory molecule, adaptor, effector, regulator, secondary receptor, sensor, signal transducer, and transcription genes) was retrieved from Deschamps et al. (2016). Immune disease genes correspond to those provided by the International Union of Immunological Societies (IUIS; December 2020) (Tangye et al. 2020). Genes with LCL-specific expression patterns were downloaded from Ryaboshapkina and Hammar (2019), and housekeeping genes were retrieved from the Housekeeping and Reference Transcript Atlas (Hounkpe et al. 2021).

### Statistical analyses

Gene set enrichment/depletion against customized gene lists (innate immunity genes, immune disease-associated genes, genes with LCL-specific expression, and housekeeping genes) was tested using the Fisher's exact test (two-sided). For enrichment analyses involving multiple comparisons (*Alu*, CDS, and APPRIS principal transcript overlap with exonic parts, and positively selected genes across splicing gene classes), the Fisher's exact test (two-sided) with Bonferroni *P*-value correction was applied. IU differences between annotated and novel isoforms were assessed using the Wilcoxon rank-sum test (two-sided). Pi diversity and phastCons score differences between different groups of exonic parts were assessed using the Wilcoxon test (two-sided) with *P*-value adjustment (Holm method). *d*_N_/*d*_S_ differences between gene classes were evaluated using the Dwass–Steel–Critchlow–Fligner all-pairs test (single-step *P*-value correction). Isoform/exon usage and global gene expression differences were tested using the statistical methods described in the DEXSeq and DESeq2 packages, respectively (Anders et al. 2012; Love et al. 2014).

## Data access

The transcriptomic data generated in this study have been submitted to the NCBI BioProject database (https://www.ncbi.nlm.nih.gov/bioproject/) under accession number PRJNA797478 and CNGB Sequence Archive (CNSA; https://db.cngb.org/cnsa/) of CNGBdb under accession number CNP0002604. Mass spectrometry proteomics data have been submitted to the ProteomeXchange Consortium via the PRIDE (Perez-Riverol et al. 2022; https://www.ebi.ac.uk/pride/archive/) partner repository with the data set identifier PXD031213. In-house scripts for transcriptome data processing are available at GitHub (https://github.com/lfp1/isoseq_primate_LCLs/tree/XiaoyuZhan) and as a Supplemental Code file.

## Supplementary Material

Supplemental Material
