# Supplementary material for: Transcriptome innovations in primates revealed by single-molecule long-read sequencing
Source: Genome Res. 2022 Aug;32(8):1448–62. doi: 10.1101/gr.276395.121 (PMC9435740; doi:10.1101/gr.276395.121)
Supplement: Supplemental Material [file supp_32_8_1448__DC1.html]

Transcriptome innovations in primates revealed by single-molecule long-read sequencing — Transcriptome innovations in primates revealed by single-molecule long-read sequencing — Supplemental Material 

# Transcriptome innovations in primates revealed by single-molecule long-read sequencing

## Supplemental Material

- Supp\_Data\_S1.zip
- Supp\_Data\_S2.zip
- Supp\_Data\_S3.zip
- Supp\_Data\_S4.zip
- Supp\_Data\_S5.zip
- Supp\_Data\_S6.zip
- Supp\_Data\_S7.zip
- Supp\_Data\_S8.zip
- Supp\_Data\_S9.zip
- Supp\_Data\_S10.zip
- Supp\_Data\_S11.zip
- Supp\_scripts.zip
- Supplemental\_Materials.pdf
