# Supplementary material for: Transcriptome innovations in primates revealed by single-molecule long-read sequencing
Source: Genome Res. 2022 Aug;32(8):1448–62. doi: 10.1101/gr.276395.121 (PMC9435740; doi:10.1101/gr.276395.121)
Supplement: Supplemental Material [file supp_gr.276395.121_Supplemental_Materials.pdf]

Supplemental Materials

Transcriptome innovations in primates revealed by single-molecule long-read sequencing

Luis Ferrández-Peral, Xiaoyu Zhan, Marina Álvarez-Estapé, Cristina Chiva, Paula Esteller-Cucala, Raquel García-Pérez, Eva Julià, Esther Lizano, Òscar Fornas, Eduard Sabidó, Qiye Li, **Tomàs Marquès-Bonet*, David Juan*, Guojie Zhang***

This file contains the description of:

- **Supplemental Methods**
- **Supplemental Figures S1-S47**
- **Supplemental Tables S1-S11**
- **Supplemental Data S1-S11**
- **Supplemental References**

Supplemental Methods

Iso-Seq data processing

All PacBio Sequel subreads generated per species, including size-selected and non-size-selected, were combined to produce circular consensus sequences (CCS), and only sequences with 5' and 3' primers and poly(A) tail were selected for further analyses (full-length non-chimeric CCS; FLNC) following the IsoSeq3 workflow (PacBio SMRTAnalysis software). Then, FLNC sequences were clustered by Iterative Clustering and Error Correction (ICE) and corrected by Arrow algorithm, generating a set of full-length polished transcript sequences per species.

Hybrid correction of full-length polished transcript sequences was carried out by LoRDEC (Salmela and Rivals 2014) using paired-end Illumina RNA-seq data for the same LCLs used in Iso-Seq (parameters: -t 5 -b 200 -e 0.4 -s 3 -k 18). Here, three technical replicates of RNA-seq for each LCL were used except for GM12878, for which publicly available RNA-seq datasets (two technical replicates) were used (NCBI accession SRR998197 and SRR998198).

After poly(A) trimming (trim_isoseq_polyA script from official PacBio Github repository with parameters -t 1 -G), Iso-Seq transcript sequences were mapped to the corresponding reference genomes -hg38 (human), panTro5 (chimpanzee), gorGor4 (gorilla), ponAbe2 (orangutan), and rheMac8 (rhesus macaque)- using GMAP (Wu and Watanabe 2005) with parameters -f samse -n 0. Reference assemblies and all Illumina RNA-seq data produced for each species were used to further attenuate the errors derived from Iso-Seq and refine transcript splice junctions with TranscriptClean (default parameters) (Wyman and Mortazavi 2019).

Uniquely mapped transcript sequences (*i.e.*, those mapping to a single genome region) were used as input for Cupcake ToFU scripts (https://github.com/Magdoll/cDNA_Cupcake, default parameters). Here, low-quality alignments were discarded based on identity (<=95%) and coverage (<=99%), and transcript sequences were collapsed into unique isoforms. This step removes the remaining redundancy accounting for 5' end variability since ICE performs a very conservative clustering.

Artifact filtering from Iso-Seq data

Isoform FASTA files were provided to SQANTI (Tardaguila et al. 2018) for quality control and artifact filtering. Here, the corresponding Ensembl annotation (V91) from each species was defined as the reference transcriptome. Furthermore, splice junction coverage by RNA-seq and isoform abundances (full-length reads from Iso-Seq and TPM calculated from RNA-seq) were used to train the SQANTI classifier.

Splice junction coverage in Iso-Seq isoforms across LCLs was obtained by mapping RNA-seq of the same LCLs used in Iso-Seq to the corresponding reference genome, as described in 'RNA-seq data production and processing'. Normalized quantification estimates (TPM) for Iso-Seq isoforms from each species were computed in the LCLs of that species using kallisto-quant (Bray et al. 2016) with --rf-stranded argument (default settings), leveraging RNA-seq in the same LCLs for which we generated each long-read transcriptome. Here, the non-redundant set of Iso-Seq isoforms (after ToFU collapsing) obtained in each species was used as kallisto target transcriptome for RNA-seq pseudo-alignment (see 'Transcript expression calculation and reconstruction of transcript gains and losses' for the description of the quantification strategy in the comparative analyses).

SQANTI machine learning method (sqanti_filter.py, default parameters) was applied to the isoforms in each species to keep a set of highly reliable isoforms, discarding any potential sequencing artifacts. All subsequent analyses were performed using the set of isoforms passing SQANTI filtering in each species, and isoforms with a splice junction structure that is not annotated (SQANTI structural category was not FSM or ISM) in the corresponding reference transcriptome were defined as novel. Data manipulation was performed using SAMtools (Li et al. 2009), BEDTools (Quinlan and Hall 2010), and in-house scripts.

Classification of alternative splicing patterns

A classification of alternative splicing (AS) events was generated by SUPPA (version 2.3, parameters: -f ioe -e {SE,SS,MX,RI,FL}) (Alamancos et al. 2015) from the isoform GTF files resulting from SQANTI. Five types of AS were identified: skipping exon (SE), alternative 5' or 3' splice sites (A5SS/A3SS), retained introns (RI), and mutually exclusive exons (MX). Splicing events were considered as known if

both splice forms (inclusion and exclusion forms of the AS event) are found in Ensembl V91 transcriptomic annotations.

Chromatographic and mass spectrometric analysis

Samples were analyzed using an LTQ-Orbitrap Fusion Lumos mass spectrometer (Thermo Fisher Scientific, San Jose, CA, USA) coupled to an EASY-nLC 1000 (Thermo Fisher Scientific (Proxeon), Odense, Denmark). Peptides were loaded directly onto the analytical column and were separated by reversed-phase chromatography using a 50-cm column with an inner diameter of 75 μm, packed with 2 μm C18 particles spectrometer (Thermo Fisher Scientific, San Jose, CA, USA).

Chromatographic gradients started at 95% buffer A and 5% buffer B with a flow rate of 300 nl/min for 5 minutes and gradually increased to 22% buffer B and 78% A in 79 min and then to 35% buffer B and 65% A in 11 min. After each analysis, the column was washed for 10 min with 10% buffer A and 90% buffer B. Buffer A: 0.1% formic acid in water. Buffer B: 0.1% formic acid in acetonitrile.

The mass spectrometer was operated in positive ionization mode with nano spray voltage set at 2.4 kV and source temperature at 275°C. Ultramark 1621 was used for external calibration of the FT mass analyzer prior to the analyses, and an internal calibration was performed using the background polysiloxane ion signal at m/z 445.12003. The dynamics exclusion duration was set at 60s, with a range in mass tolerance of ±10 ppm. Each analysis used the multinotch MS3-based TMT method (McAlister et al. 2014). The scan sequence began with an MS1 spectrum (Orbitrap analysis; resolution 120 000; mass range 375–1500 m/z; automatic gain control (AGC) target 4×10^5, maximum injection time 50 ms). In each cycle of data-dependent acquisition analysis, the most intense ions above a threshold ion count of 5000 were selected for fragmentation after each survey scan. The number of selected precursor ions for fragmentation was determined by the “Top Speed” acquisition algorithm with a cycle time set at 3s. Fragment ion spectra were produced via collision-induced dissociation (CID) at a normalized collision energy of 35% and they were acquired in the ion trap mass analyzer in “Turbo” mode. AGC was set to 10^4, and an isolation window of 0.7 m/z and a maximum injection time of 50 ms were used. Following the acquisition of each MS2 spectrum, MS3 spectra were collected, in which multiple MS2 fragment ions are captured in the MS3 precursor population using isolation waveforms with multiple frequency notches. MS3 precursors were fragmented by high energy collision-induced

dissociation (HCD) at a normalized collision energy of 65% and acquired in the Orbitrap analyzer at 50000 resolution. AGC was set to 10^5, and an isolation window of 2 m/z and a maximum injection time of 105 ms were used. All data were acquired with Xcalibur software v4.1.31.9.

Digested bovine serum albumin (New England Biolabs cat # P8108S) was analyzed between each sample to avoid sample carryover and to assure stability of the instrument, and QCloud (Chiva et al. 2018) has been used to control instrument longitudinal performance during the project.

Identification of novel peptides by mass spectrometry proteomics

Acquired spectra were analyzed using the Proteome Discoverer software suite (v2.3, Thermo Fisher Scientific) and the Mascot search engine (v2.6, Matrix Science (Perkins et al. 1999)). A UniProt-based database comprising all reference proteomes from all five primates was used to identify detectable genes and narrow down the search space. After removing transcripts classified as potential artifacts by SQANTI, Iso-Seq protein predictions for these genes in all species were incorporated into a customized multi-species database, together with the predicted proteins from intergenic and fusion loci. For this, NHP Iso-Seq isoforms were first lifted to the most recent assembly of the corresponding species (panTro6, gorGor6, ponAbe3, rheMac10) using UCSC liftOver with default parameters (Hinrichs et al. 2006), and protein predictions from Iso-Seq isoforms were performed by SQANTI using GeneMarkS-T algorithm (GMST) (Tang et al. 2014). All target databases included a list of common contaminants (Beer et al. 2017) and all the corresponding decoy entries. This strategy leverages the high-quality Iso-Seq isoforms detected in all species to build a comprehensive target database, which was used to identify peptides present across all samples, allowing peptide identifications from protein predictions derived from transcripts captured in a different species.

For peptide identification, a precursor ion mass tolerance of 7 ppm was used for MS1 level, trypsin was chosen as the enzyme, and up to three missed cleavages were allowed. The fragment ion mass tolerance was set to 0.5 Da for MS2 spectra. Oxidation of methionine and N-terminal protein acetylation were used as variable modifications, whereas carbamidomethylation on cysteines, TMT6plex in Lysines and TMT6plex in peptide N-terminal were set as a fixed modification. The false discovery rate (FDR) in peptide identification was set to a maximum of 5%, and peptides were quantified using the reporter ion intensities in MS3. According to manufacturer's specifications, reporter ion intensities were adjusted to

correct for the isotopic impurities of the different TMT reagents. Reporter ion intensities from the samples were referenced to the common pool present in each of the three TMT mixes and they were used to estimate the peptide and protein fold-changes. The obtained fold-changes were then log-transformed and normalized between the channels by adjusting the mean log-fold-change to zero. We established further stringent criteria to filter for high-quality peptide identifications and ensure a good quantification signal: FDR<5%, Mascot IonScore > 20, reporter ion intensity signal (abundance) per sample > 50 in any sample of a given species, and species-wise median ratio of sample abundance to pool abundance > 0.6, after discarding contaminant matches and peptides arising from more than 1 tryptic miscleavage. As a quality control, we projected Iso-Seq transcripts from all species to each species' genome using UCSC liftOver (default parameters) (Hinrichs et al. 2006), and predicted the proteins for each species. We only kept the high-quality identified peptides in a given species that were tryptic in the projected proteome (see 'Detected peptides and plausible in genome' in **Supplemental Table S7**).

Then, the resulting sets of peptides identified in each species were compared to the corresponding reference proteome from UniProt (based on hg38, panTro5, gorGor4, ponAbe2 and rheMac8) and RefSeq (CDS set from hg38, panTro6, gorGor6, ponAbe3 and rheMac10). To do so, we performed *in-silico* tryptic digestion of UniProt and RefSeq proteomes (allowing a maximum of 1 miscleavage). High-quality identified peptides in a given species that are absent in the digestions of UniProt or RefSeq proteomes for the corresponding species were defined as novel.

Data processing was performed using EMBOSS (Rice et al. 2000), SeqKit (Shen et al. 2016), and in-house scripts. Tryptic peptides were mapped to their genomic coordinates using PGx (Askenazi et al. 2016).

Definition of orthology relationships

To define one-to-one orthologous genes in the five primate species, peptide sequences, gene annotations and genome assemblies were retrieved from Ensembl, using the same genomes as in Iso-Seq mapping (hg38, panTro5, gorGor4, ponAbe2, and rheMac8). Considering that one gene may contain multiple protein isoforms, the longest isoform with a complete ORF was selected as representative. Then, BLASTP (v2.2.26) (Altschul et al. 1990) was used for peptide sequence

comparison between NHP and humans with an e-value limit of 1E-5 and SOLAR (v0.9.6) was used to combine local alignments (Almasy and Blangero 1998). Only reciprocal best hits (RBH) following synteny conservation were defined as one-to-one orthologous genes. One RBH gene pair (A1A2; 1 and 2 denote two different species) and its nearest RBH gene pair (B1B2) were considered syntenic if they met the following requirements: a) genes A1 and B1 are on same chromosome/scaffold; b) genes A2 and B2 are on same chromosome/scaffold; c) the number of genes located between A1 and B1 < 5; d) the number of genes between A2 and B2 < 5. We also retained RBH gene pairs if the corresponding scaffold has only one gene annotated. Then, we intersected these results with Ensembl Compara database (V91) (Yates et al. 2020), and kept the genes where one-to-one orthologues in the five species were consistent in both methods.

For isoforms produced by one-to-one orthologous genes, UCSC liftOver (Hinrichs et al. 2006) was used to identify the genome coordinates of orthologous transcripts using available whole-genome alignments for hg38, panTro6, gorGor6, ponAbe3 and rheMac10. Genomic coordinates of NHP isoforms (BED12) were mapped to the most recent assemblies (default parameters in UCSC liftOver), and then to hg38 (tolerating nucleotide differences with -minMatch=0.5 in UCSC liftOver). To reduce the mapping and quantification noise associated with very small differences in transcript sequences, the variability of internal exon ends and transcript extremes from each transcript model (derived from any species) was collapsed using TAMA merge in hg38 reference genome (parameters: -a 100 -m 100 -z 100 -e longest_ends) (Kuo et al. 2020). Then, these non-redundant transcript models were mapped to NHP (-minMatch=0.5 in UCSC liftOver).

For the comparative analyses (species-specific gains and losses, and DIU), we kept the transcript models effectively mapped to all five species. This strategy addresses the unbalanced Iso-Seq isoform repertoire across species (**Supplemental Table S3**) resulting from the lack of experimental saturation. As explained below, the expression of orthologous transcripts/exons are calculated using RNA-seq data from each species (3 LCLs per species) mapped to the corresponding transcript/exons in that species. Furthermore, for each species, we used SQANTI to characterize the orthologous transcript models in the context of the most recent genome assemblies and their reference annotations (Ensembl for hg38, RefSeq for panTro6, gorGor6, ponAbe3 and rheMac10).

We followed an analogous strategy without any transcript collapsing in hg38 to account for smaller splicing changes (*e.g.,* alternative donor and acceptor sites) that were quantified by DEU analyses (TAMA merge parameters: -a 0 -m 0 -z 0).

Species-specific exon gains (genomic structure)

We restricted these analyses to the set of transcripts derived from one-to-one orthologous genes. NHP exons which failed to be mapped to the human reference genome (hg38) and human exons which failed to be mapped to all four NHP by UCSC liftOver (default settings) were chosen as species-specific exon candidates. Then, Liftoff (Shumate and Salzberg 2020) with default settings was used to perform a local alignment of exon candidates to the remaining four assemblies. Only exons with an alignment coverage (length of aligned exonic region / total exon length) lower than 50% against all the remaining four assemblies were selected as species-specific exonic structures. The species-specific exons and conserved exons were intersected with repetitive regions (UCSC RepeatMasker track files for hg38, panTro6, gorGor6, ponAbe3, and rheMac10) and with CDS/UTR transcript annotations provided by SQANTI.

To assess how these exons might impact protein function, we evaluated changes in the combination of protein domains in the encoded protein. To do so, the encoded proteins in transcripts with species-specific exons (SQANTI predictions) and all protein sequences from Ensembl (hg38, panTro5, gorGor4, ponAbe2, and rheMac8) were searched against Pfam database using InterPro (Blum et al. 2021) (default settings) to retrieve their protein domains. Protein domain structures (including number and specific order of domains) resulting from exonization events were compared to the set of protein domains found in the complete Ensembl proteomes.

Transcript expression calculation and reconstruction of transcript gains and losses

Considering that isoform discovery rates (saturation curves) did not reach the *plateau* for Iso-Seq data, we incorporated deep RNA-seq (3 LCLs per species; Human: GM12878, GM19150, GM19238; Chimpanzee: CH114, CH391, CH170; Gorilla: DIAN, OMOYE, GG05; Orangutan: PPY6 (also named PPY6_1), EB185, CRL-1850; Macaque: R02027, R05040 and R94011) to quantify the projected transcript models in each genome using kallisto (Bray et al. 2016). Kallisto-quant function with --rf-

stranded argument (default settings) was used for pseudo-alignment of RNA-seq reads (3 LCLs per species) to the projected transcript models in the corresponding species (target transcriptome). This allowed the estimation of relative transcript abundances (transcripts-per-million, TPM).

Since some orthologous transcripts projected to a given species might not be supported for RNA-seq in their splicing structure (*e.g.*, if there has been a change in splice sites between species), we evaluated the RNA-seq coverage in splice junctions (sample-wise) prior to kallisto quantification. For each LCL, transcripts that were unsupported by RNA-seq in their splice junctions were excluded from kallisto calculation and assigned 0 TPM (for subsequent analysis, we excluded mono-exonic transcripts). This strategy implies a very strict definition of orthologous transcripts, where small changes in splice sites across species are detected since they will affect the calculation of isoform abundances. Batch effects in transcript expression resulting from three different RNA-seq experimental batches were corrected using ComBat (default settings) (Zhang et al. 2020) as shown in **Supplemental Fig. S47.**

Count (Csűös 2010) was used to reconstruct the tree of transcript expression gains and losses in primates considering the known phylogenetic structure and the resulting binary matrix of transcript expression across species. A transcript was defined as expressed if TPM>0 in at least two LCLs from a given species (less conservative criteria, ‘All data’ in **Supplemental Fig. S14**). The used species tree (Newick format) was the following:

(Macaca_mulatta:29.44154682,((Gorilla_gorilla:9.06309552,(Pan_troglodytes:6.65090500,Homo_sapiens:6.65090500)'14':2.41219052)'13':6.69907002,Pongo_abelii:15.76216554)'11':13.67938128);

Wagner parsimony was used with a relative penalty of gain-to-loss equal to 1. We also retrieved the number of transcript gains and losses that can be explained by a unique gain or loss event in our phylogeny and compared them to the corresponding gains and losses inferred by Wagner parsimony reconstruction for the same branches by calculating the ratio between them (**Supplemental Fig. S14**).

To exclude intra-species variation and identify species differences in the detection of transcript gains and losses, we further asked for strict intra-species consistency in the presence/absence of RNA-seq-based transcript expression (short-read-based replicability). Hence, for each transcript we require that the 3 biological replicates (LCLs) from each species are coherent in the pattern of expression/absence of expression. Thus, species-specific transcripts (isoform innovations) are expressed in all LCLs from

a given species (TPM>0) and absent (TPM=0) in all LCLs from the remaining species. These criteria increase the percentage of transcript gains and losses that are coherent with the phylogeny (*i.e.*, being gained or lost just once in the phylogeny) in comparison to the total set of transcripts (**Supplemental Fig. S14**).

To inspect the contribution of species-specific splicing events to species-specific transcripts, we retrieved the splice junctions conservation level (number of species in which they are supported) from the set of transcripts showing intra-species consistency in their expression (see above). Similar to transcript conservation analyses, we asked for intra-species consistency in the patterns of junction support by RNA-seq. In this way, species-specific junctions are supported in all LCLs from a given species (junction reads > 0), and unsupported (junction reads = 0) in all LCLs from the rest of species. For these splice junctions (non-redundant set), RNA-seq support across GTEx v6 samples was retrieved using the snapcount package (Wilks et al. 2021) (hg38 exact coordinates query within tissue_specificity function). To be considered supported in a given human tissue, we require that the splice junction is used in at least 20% of the GTEx samples for the corresponding tissue.

The proportion of AS classes was compared between species-specific junctions and junctions used in all five primates (SUPPA definition, same parameters as in 'Classification of alternative splicing patterns'). Both inclusion and exclusion forms must exist in the transcriptome to define an AS event. Splice junctions were associated with a given AS class if they matched either the inclusion or the exclusion form. We excluded RI from this comparison since our quantification strategy relies on RNA-seq support in transcript splice junctions, without taking into account RNA-seq coverage in the boundary between an exon and a retained intron.

Differential gene expression analyses

To assess the effect of gene expression in the detection of species-specific transcripts, differential gene expression analyses were performed using DESeq2 (pairwise comparatives) (Love et al. 2014), including the 3 RNA-seq biological replicates per species. Genes with 10 or more RNA-seq reads accumulated across samples were retained. Species-specific up-regulated genes were defined as those showing significant overexpression (permissive padj<0.1 and log_2FoldChange>0 were used to detect even subtle influences of gene expression changes in isoform detection) in a given species

compared to all the rest, regardless of the possible gene expression differences among the remaining species. Then, genes up-regulated in each species were intersected with those expressing species-specific transcripts in the corresponding species.

We followed an analogous strategy to define species-specific down-regulated genes (padj<0.1, log_2FoldChange<0). Species-specific up- and down-regulated genes were intersected with those displaying species-specific up- or down-regulated exon usage in the same species.

Differential isoform usage analyses

Kallisto transcript quantifications were used to evaluate interspecies isoform usage (IU) changes (Soneson et al. 2015). ComBat batch effect correction (Zhang et al. 2020) and TMM (Trimmed Mean of M-values) normalization were performed prior to this calculation. IU values for orthologous transcripts were calculated across all samples using IsoformSwitchAnalyzeR package (Vitting-Seerup and Sandelin 2019). Then, principal component analysis (PCA) and hierarchical clustering based on the euclidean distances of Spearman correlations for IU across samples were computed.

To measure the most confident isoform usage changes, we first excluded genes in which the transcript models resulted from the collapsing of internal exon boundaries by TAMA merge, since this variability in internal exon ends can lead to artificial isoform usage differences considering our quantification strategy (see 'Transcript expression calculation and reconstruction of transcript gains and losses'). In addition, low expression genes/isoforms and genes with a single isoform were removed using IsoformSwitchAnalyzeR preFilter function (default settings: geneExpressionCutoff = 1 TPM, isoformExpressionCutoff = 0 TPM, IFcutoff = 0.01, removeSingleIsoformGenes = TRUE). Differential isoform usage (DIU) analyses were performed by establishing pairwise comparatives across the five species using DEXSeq method in IsoformSwitchAnalyzeR (Vitting-Seerup and Sandelin 2017; Ritchie et al. 2015; Anders et al. 2012). Species-specific DIU cases were defined as significant isoform usage changes (|dIU|>0.1 and isoform switch q-value<0.05) in a given species compared to the rest of primates (up- or down-regulated), while the remaining species showed non-significant differences among them. The same rationale was applied to classify isoform usage changes shared by groups of 2 or 3 species in comparison to the rest of them. IsoformSwitchAnalyzeR preFilter function (default

settings) was also used for the evaluation of genes where orthologous splice junctions showed high-impact genetic changes across primates.

Differential exon usage analyses

The projected transcript models in each species (without any collapsing, see 'Definition of orthology relationships') were flattened to define orthologous exonic counting bins from all transcribed segments (*i.e.*, exonic parts). RNA-seq read counts overlapping each exonic part in the 3 LCLs from each species were obtained using HTSeq-count (parameters: -p yes -r pos -s reverse -f sam) (Anders et al. 2015) after discarding multi-mapping reads. Differential exon usage (DEU) analyses were performed by establishing pairwise comparatives across the five species using DEXSeq (Anders et al. 2012; Reyes et al. 2013) while controlling for batch effects. Significant exon usage changes (exonBaseMean>10, $|\log_2 FC|>1.2$ and padj<0.05) were retrieved from each pairwise comparative and used to classify the change pattern across species. Exonic parts showing significant difference in usage in a given species compared to the rest of primates (up- or down-regulated) were defined as species-specific as long as the remaining species did not display significant differences among them. The same rationale was used to classify changes shared by groups of 2 or 3 species compared to the remaining species. Exonic parts not classified as DEU in any pairwise comparative and displaying exonBaseMean>10 (average across all samples) were defined as conserved.

To quantify the frequency of exon inclusion, we calculated percent-spliced-in (PSI) estimates according to (Schafer et al. 2015) and computed the average across the 15 RNA-seq experiments (3 LCLs per species). Average PSI estimates were used to classify exonic parts into highly excluded [0-0.2), middle excluded [0.2-0.4), middle included [0.4-0.8) and highly included [0.8-1] (**Fig. 5A**). We classified the exonic parts into AS modes using SUPPA in the projected transcript models (see 'Classification of alternative splicing patterns'). The phastCons conservation scores across primates were retrieved from UCSC whole-genome alignments (17-way phastCons scores). Average phastCons scores per exonic part were computed using UCSC bigWigAverageOverBed tool (default settings), and then classified into low [0-0.3], middle (0.3-0.8) and high [0.8-1] (**Fig. 5A**). Nucleotide diversity (pi) in human populations was calculated from the data generated by the 1000 Genomes project (The 1000 Genomes Project Consortium 2015) mapped against hg38 using VCFtools (version 0.1.12, VCFtools --site-pi,

default settings) (Danecek et al. 2011). The 1000 Genomes strict mask was used to select exonic parts with all their nucleotides classified as passed bases according to the strict mask BED file (hg38). Average pi estimates were also computed for each exonic part.

Classification of genes according to their splicing and usage patterns

One-to-one orthologous genes expressed in LCLs were classified according to the splicing and usage changes detected in the analyses of transcript expression, DIU, and DEU.

To do so, isoforms and exonic parts were first classified as conserved (if the expression or usage is conserved in all species), species-specific gains (up-regulation in a single species in comparison to the rest), and other changes (*i.e.*, the remaining cases resulting from expression or usage differences between groups of species, or species-specific losses). Isoforms and exonic parts showing usage patterns that are not consistent with differences between groups of species (*e.g.,* differential usage in only one pairwise comparative*)* were not considered as they are more likely reflecting intra-species variability.

The above classification of isoforms and exonic parts resulting from the analyses of transcript expression, DIU, and DEU were combined to establish five gene classes. Each gene was classified according to the following rules:

1) 'all_conserved': genes where only conserved isoforms/exonic parts were detected. Thus, these genes produce isoforms expressed in all five species, and no isoform/exon usage differences were found across species.
2) 'only_human_sp': genes where human-specific gains were detected (and not species-specific gains from other species), allowing the presence of conserved or other isoforms/exonic parts. Thus, these genes produce human-specific transcripts, or show human-specific isoform/exon usage gains.
3) 'only_NHP_sp': genes where only chimpanzee, gorilla, orangutan or macaque-specific gains were detected (for a single species, excluding human), allowing the presence of conserved or other isoforms/exonic parts. Thus, these genes produce species-specific transcripts in NHP, or show species-specific isoform/exon usage gains in NHP.
4) 'convergence_spsp': genes where independent events of species-specific gains were detected in multiple species, allowing the presence of conserved or other isoforms/exonic parts. Thus,

these genes produce species-specific transcripts in at least 2 primate species, or show species-specific isoform/exon usage gains in at least 2 primate species.

5) ‘other’: genes where other patterns were detected (different than species-specific gains), allowing the presence of conserved isoforms/exonic parts. Thus, these genes do not produce species-specific transcripts or show species-specific isoform/exon usage gains, but they show combinations of other evolutionary changes in isoform expression, DIU or DEU (for example, isoforms only expressed in human, chimpanzee and gorilla, or up-regulated exon usage in human and chimpanzee compared to the rest of species).

Supplemental Figures

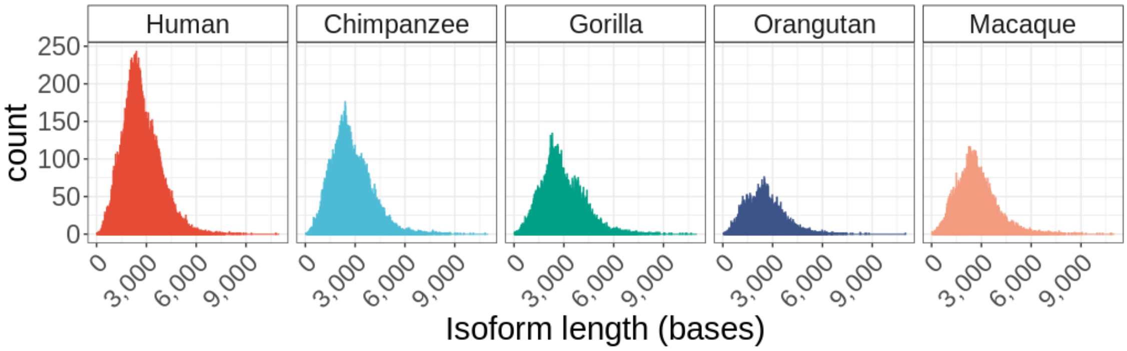

Supplemental Figure S1. Isoform length distribution of Iso-Seq transcriptomes from each species after SQANTI filtering.

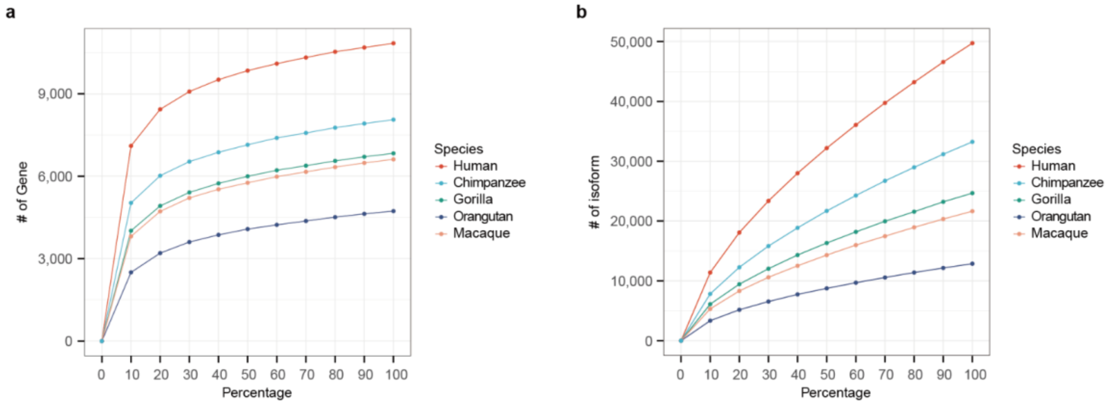

Supplemental Figure S2. Saturation analysis of Iso-Seq data for gene **(a)** and isoform **(b)** discovery rates across the 5 primate species. Saturation curves are based on random subsampling of incremental fractions of long-read sequencing data.

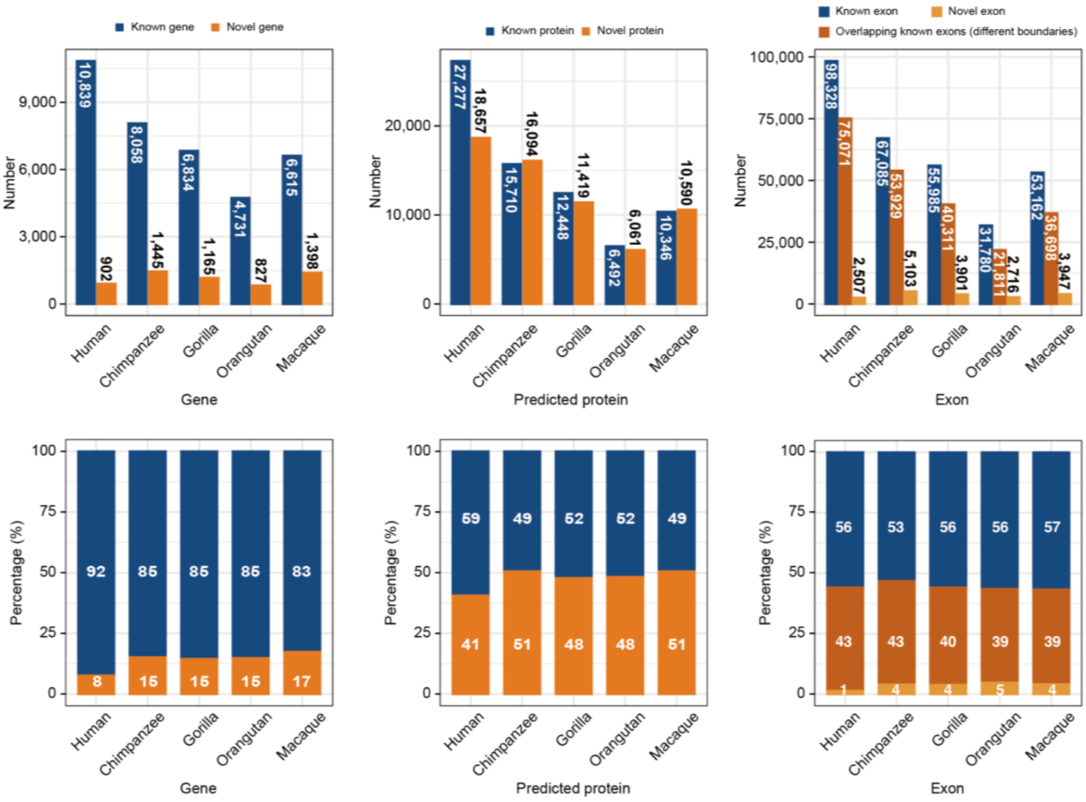

Supplemental Figure S3. Number (top) and percentage (bottom) of known vs novel genes (left), predicted protein sequences (middle) and exons (right) captured by Iso-Seq in each species.

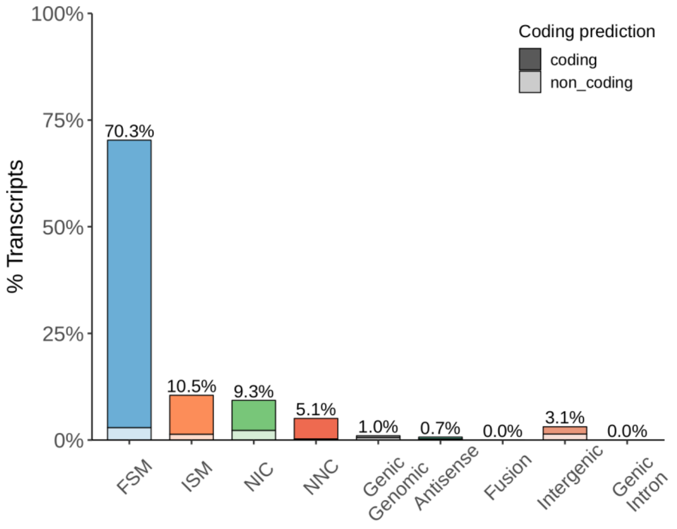

Supplemental Figure S4. Percentage of human Iso-Seq transcripts belonging to each SQANTI structural category based on the Universal Human Reference RNA (UHRR) Iso-Seq dataset. Legend indicates if the transcript has been predicted to encode a protein ('coding') or not ('non_coding'). FSM: full splice match (complete match of junctions combination reported in UHRR Iso-Seq); ISM: incomplete splice match (partial match of junctions combination

reported in UHRR Iso-Seq); NIC: novel in catalog (novel combination of reported splice sites in UHRR Iso-Seq); NNC: novel not in catalog (combination of splice sites not reported in UHRR Iso-Seq); Genic Genomic: isoform overlaps exons and introns in UHRR Iso-Seq; Antisense: isoform is anti-sense to a reported gene in UHRR Iso-Seq; Fusion: isoform simultaneously maps to two reported genes in UHRR Iso-Seq; Intergenic: isoform maps to intergenic region according to UHRR Iso-Seq; Genic Intron: isoform is fully contained in intronic region from UHRR Iso-Seq. A detailed description of SQANTI structural categories can be found in Tardaguila et al. (2018).

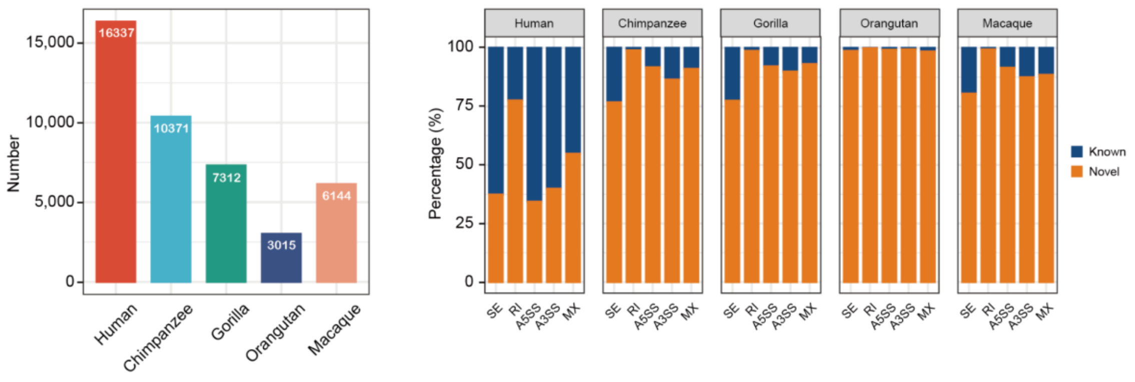

Supplemental Figure S5. Number of alternative splicing events (left) and percentage of known vs novel alternative splicing events captured by Iso-Seq in each species (right). SE: skipping exons; RI: retained introns; A5SS: alternative 5' splice sites; A3SS: alternative 3' splice sites; MX: mutually exclusive exons.

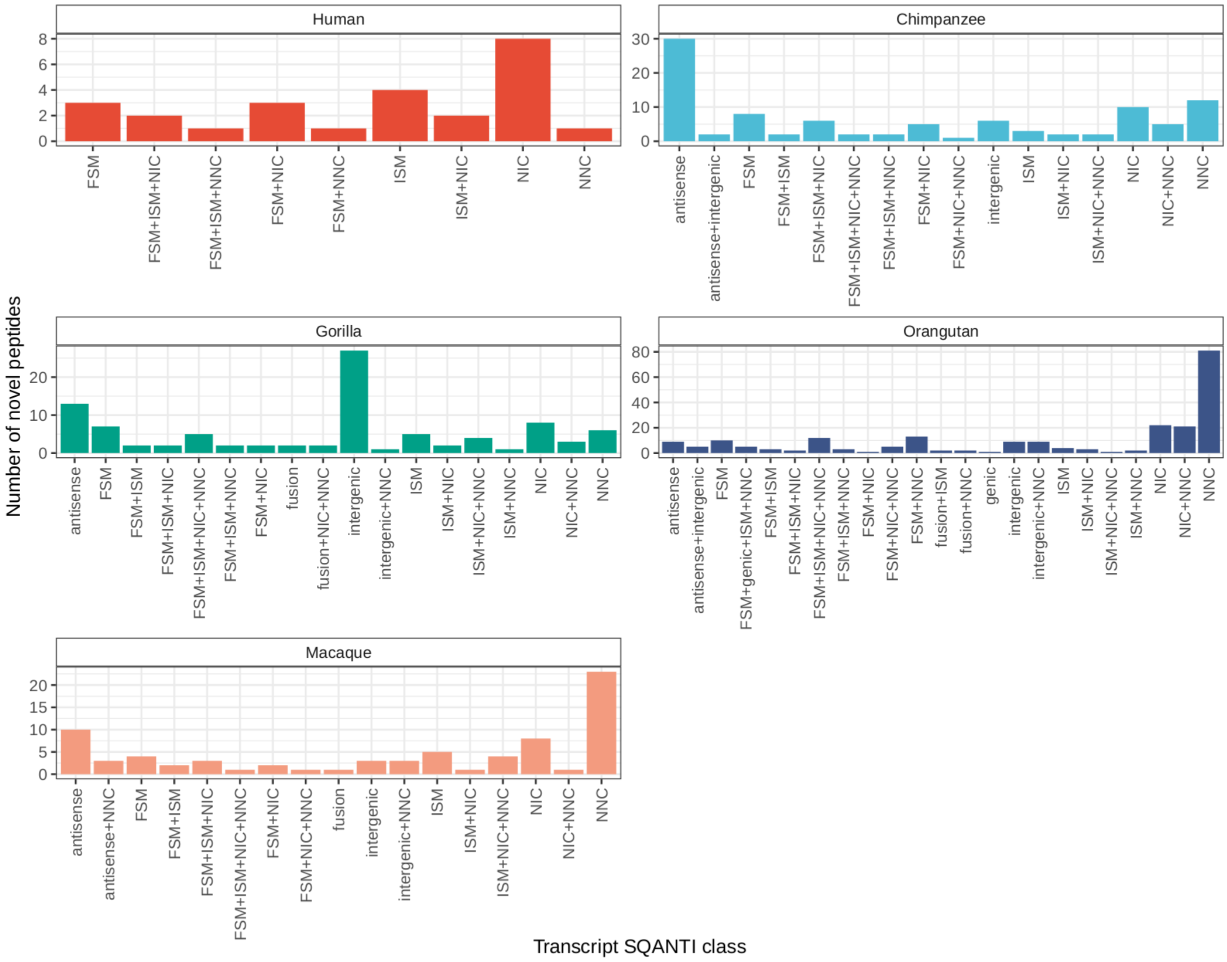

Supplemental Figure S6. Number of novel peptides detected in each species (compared to their RefSeq proteomes) by the SQANTI class of the transcript from which they were predicted. SQANTI classes are defined according to the reference transcriptome of the corresponding species (Ensembl for hg38, and RefSeq for panTro6, gorGor6, ponAbe3 and rheMac10). A detailed description of SQANTI structural categories can be found in Tardaguila et al. (2018).

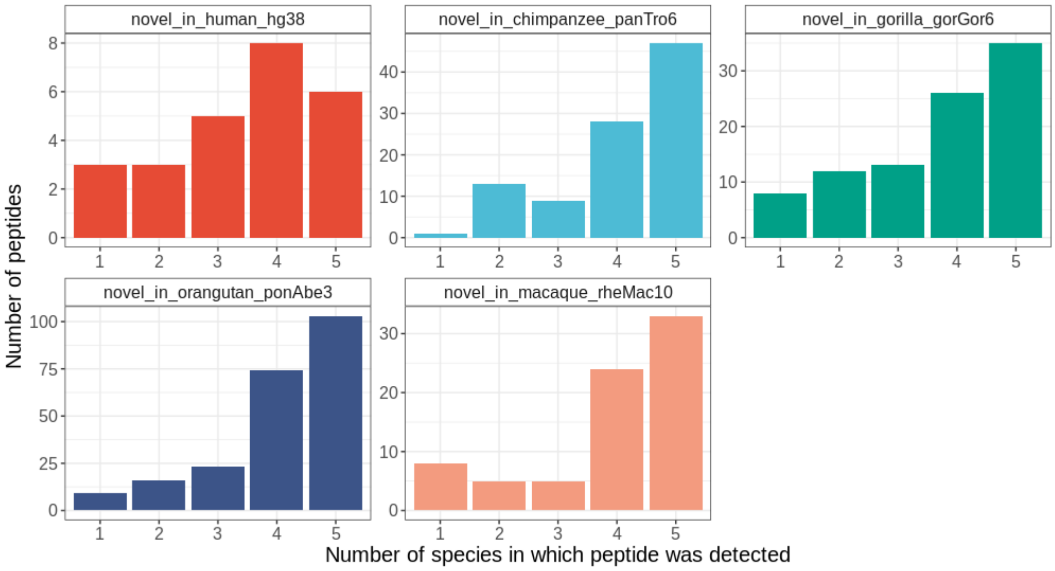

Supplemental Figure S7. Number of species in which novel peptides to a given species were simultaneously detected by mass spectrometry experiments.

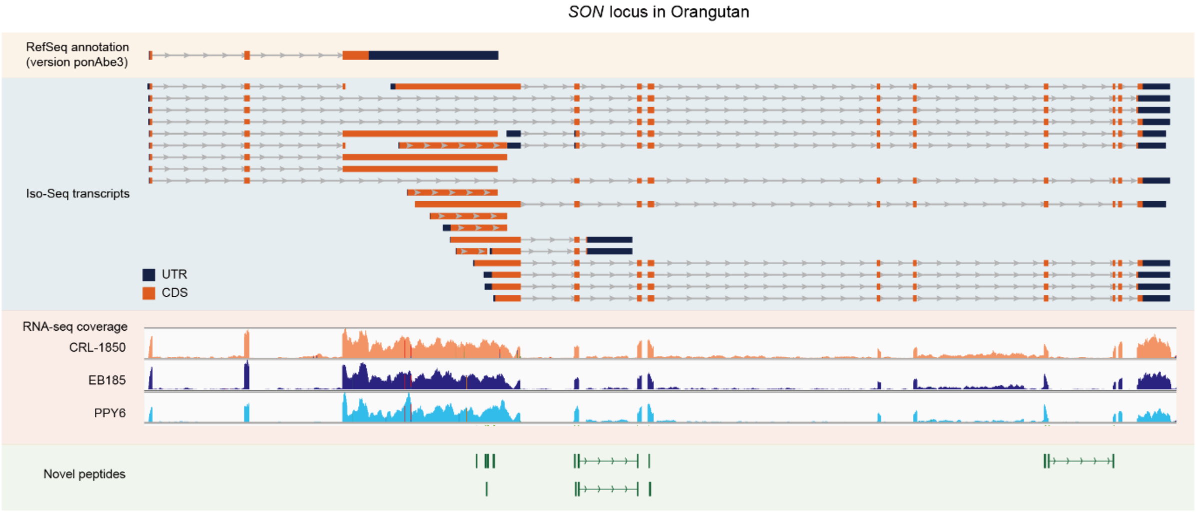

Supplemental Figure S8. Example of poorly annotated gene, *SON* (shown in ponAbe3 assembly), refined by Iso-Seq, RNA-seq and mass spectrometry in orangutan samples.

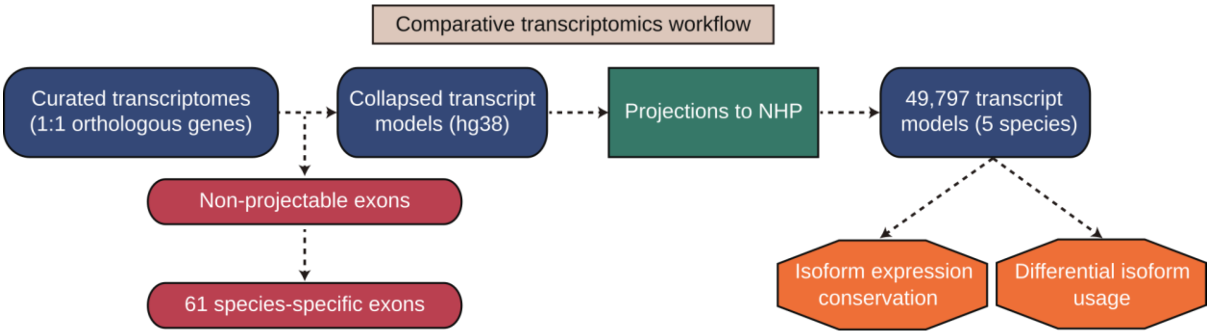

Supplemental Figure S9. Schematic workflow for comparative transcriptomics analyses based on Iso-Seq collapsed isoform models.

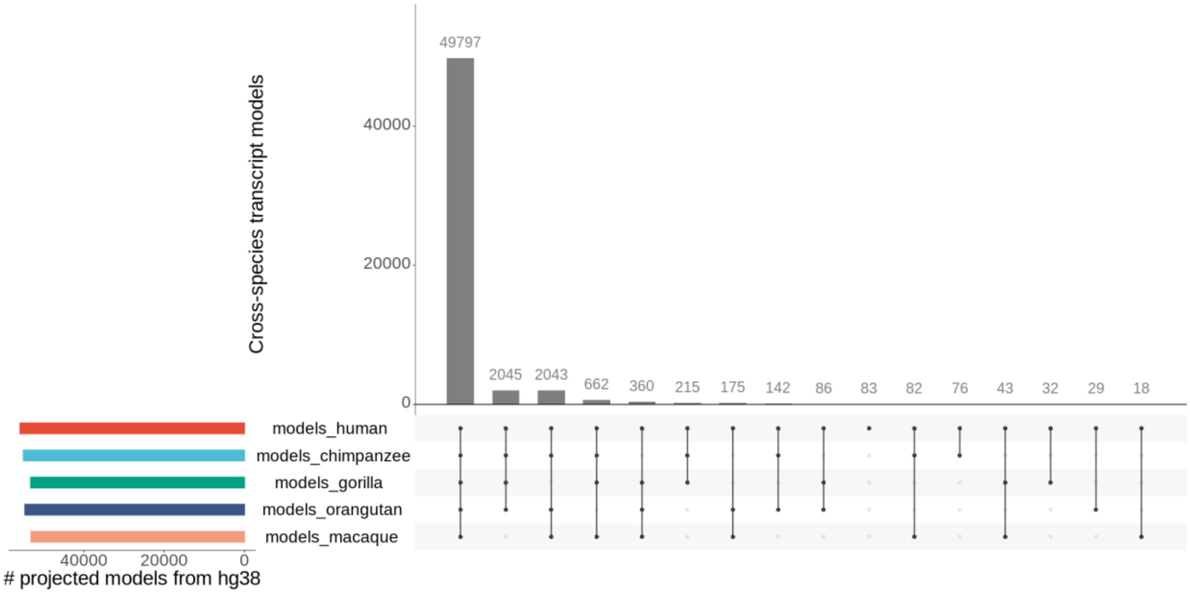

Supplemental Figure S10. Number of transcript models whose coordinates were projected to different species from all collapsed models in hg38 genome (N=55,888 models).

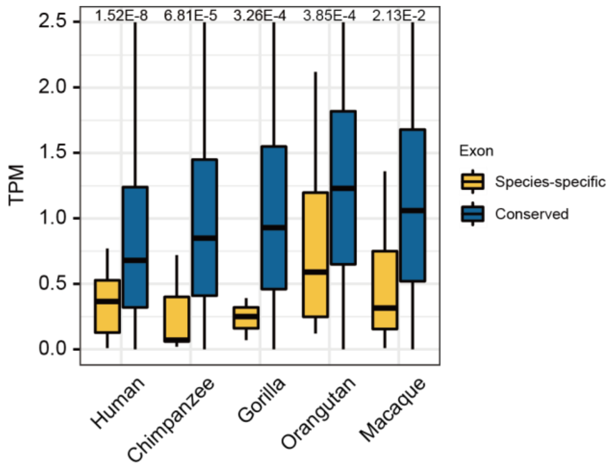

Supplemental Figure S11. Expression values for the exons present in a single species (Species-specific; yellow) and present across all species (Conserved; blue). Exon expression was normalized to Transcripts Per Million (TPM). Statistical significance of the difference across groups was assessed by Student t-test. P-values are shown for the comparison in each species.

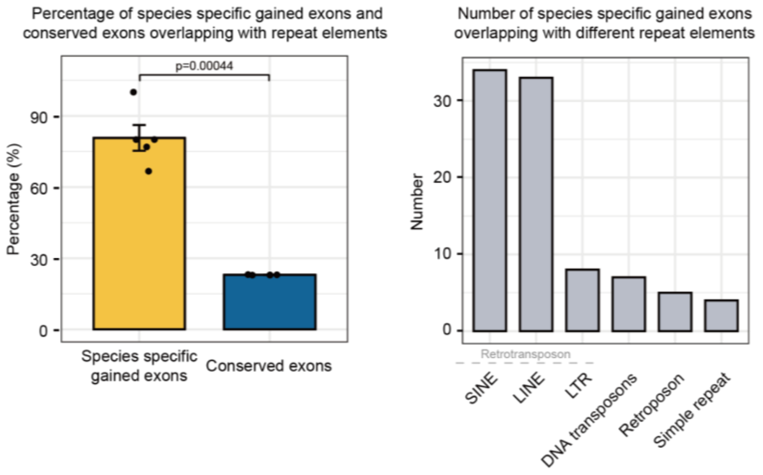

Supplemental Figure S12. Percentage of species-specific gained (yellow) and conserved (blue) exons overlapping repeat elements (left) and number of species-specific gained exons overlapping different classes of repeat elements (right). Statistical significance of the difference across groups was assessed by Student t-test.

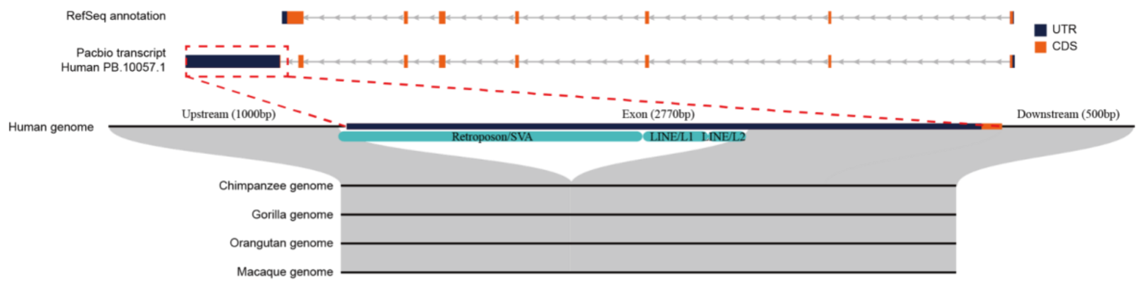

Supplemental Figure S13. Example of a gene, *MRNIP*, expressing a human-specific exon in 3’ UTR. RefSeq *MRNIP* models in hg38 assembly (top track) are illustrated together with human PB.10057.1 Iso-Seq transcript (middle track). Human-specific insertions of transposable elements (SVA and LINEs) in 3’ UTR are also depicted (bottom track). UTR and CDS are colored in blue and orange, respectively. CDS: coding sequence; UTR: untranslated region.

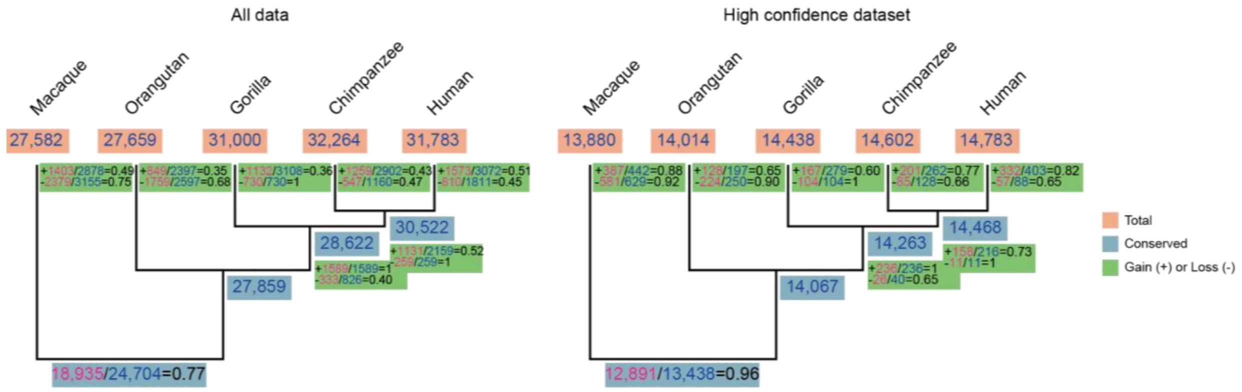

Supplemental Figure S14. Transcript gains and losses in the primate lineage in all projected transcripts (‘All data’, left) and high confidence transcripts (‘High confidence dataset’, corresponding to transcripts whose expression is consistent in all samples from each species, right). Numbers in blue indicate the gains and losses inferred from Wagner parsimony, whereas numbers in pink correspond to the number of transcript gains and losses that can be explained by a unique gain or loss event in our phylogeny.

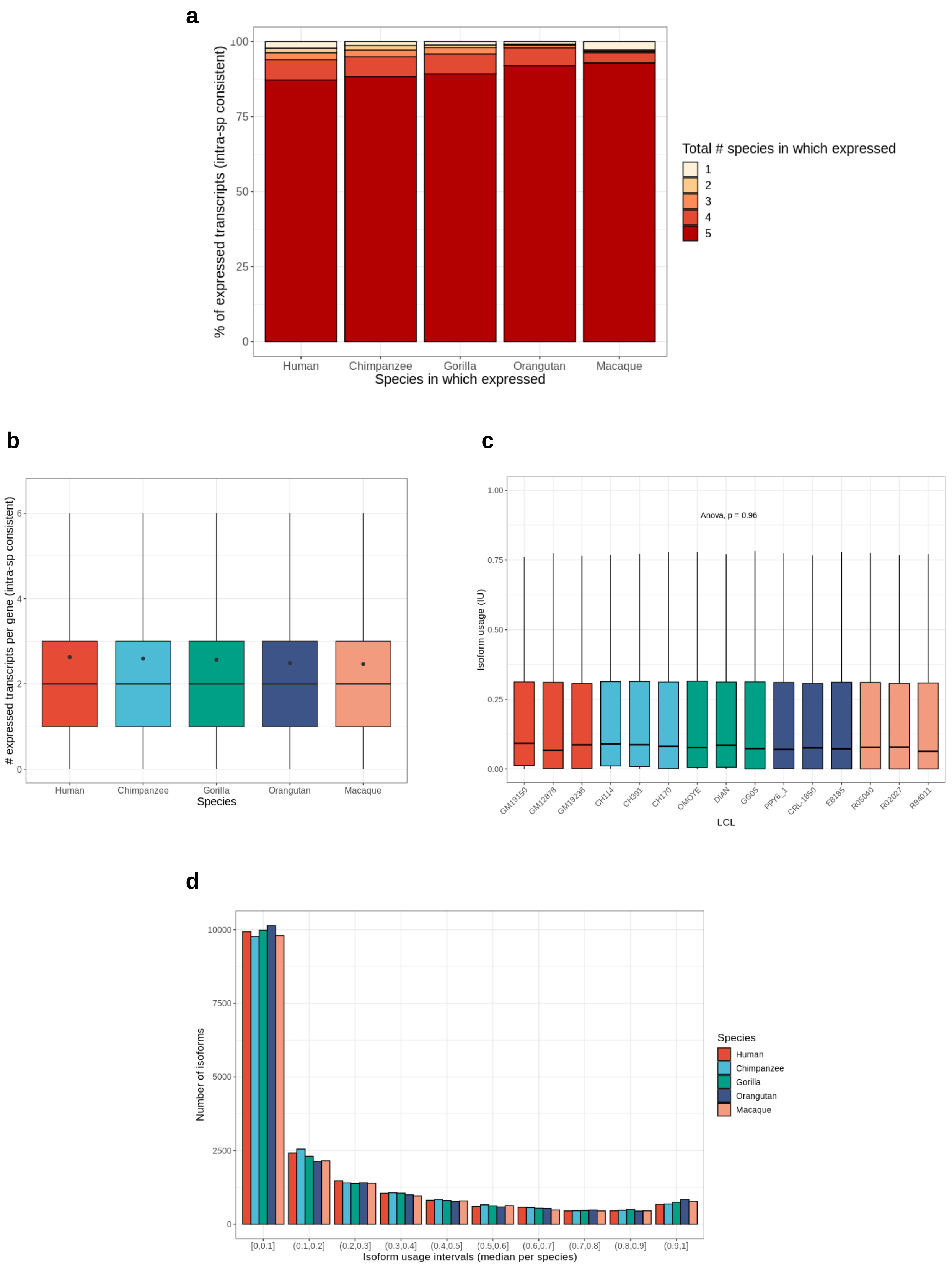

Supplemental Figure S15. Transcriptome complexity across 5 primate species. **a)** Patterns of isoform expression conservation. **b)** Number of expressed transcripts per gene. Distribution of isoform usage values across LCLs **(c)** and species **(d)**.

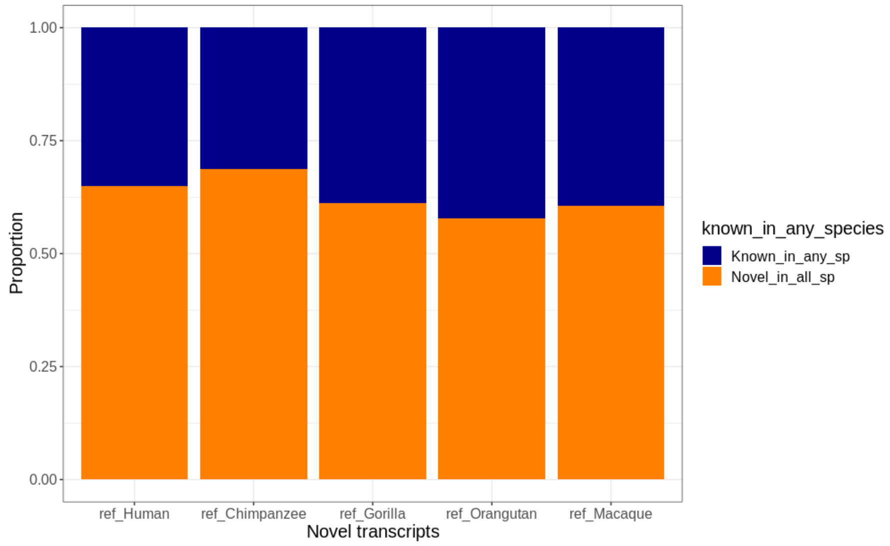

Supplemental Figure S16. Novel transcripts in each species' reference annotation are classified in two groups, depending on their annotation level in other species ('Known_in_any_sp': the isoform is annotated in at least one species; 'Novel_in_all_sp': the isoform is novel in all species' reference annotations). Only novel transcripts showing intra-species consistency in their expression patterns are shown.

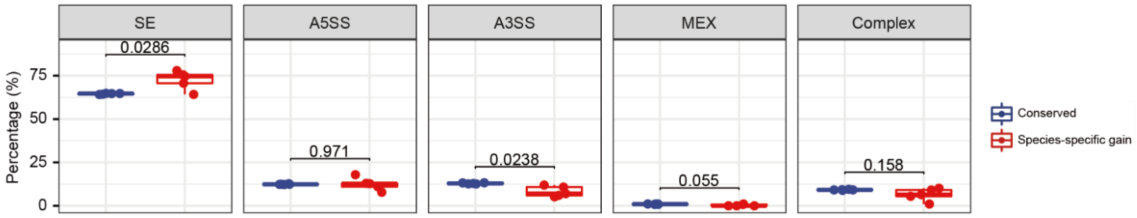

Supplemental Figure S17. Percentage of alternative splicing modes in junctions used in all five species ('Conserved', blue boxes) and junctions with species-specific usage ('Species-specific gain', red boxes). SE, skipping exons; A5SS, alternative 5' splice site; A3SS, alternative 3' splice site; MEX, mutually exclusive exons; Complex, any combination of the previous AS modes. Retained introns (RI) were excluded from this analysis (see Supplemental Methods). Statistical significance of the difference across groups was assessed by Student t-test. Shown p-values were corrected using the Holm method.

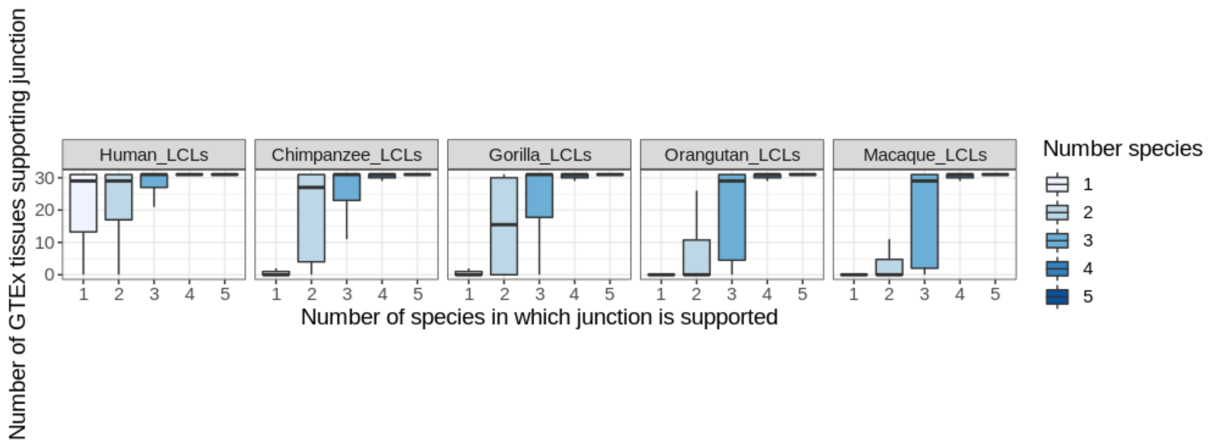

Supplemental Figure S18. Number of GTEx tissues supporting splice junction usage. The junctions used by each species' LCLs are segregated by conservation level (number of species in which they are simultaneously expressed). Only junctions showing intra-species consistent usage patterns in intra-species consistently expressed isoforms are shown. For a given splice junction to be supported in a given GTEx tissue, we require that at least 20% of all samples from that tissue display RNA-seq reads spanning the junction.

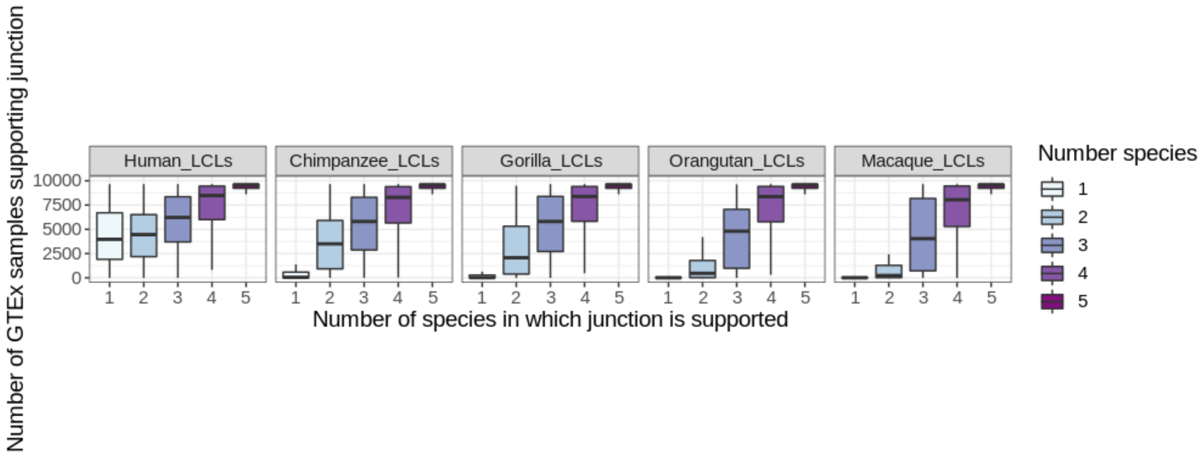

Supplemental Figure S19. Number of GTEx samples supporting splice junction usage. The junctions used by each species' LCLs are segregated by conservation level (number of species in which they are simultaneously expressed). Only junctions showing intra-species consistent usage patterns in intra-species consistently expressed isoforms are shown.

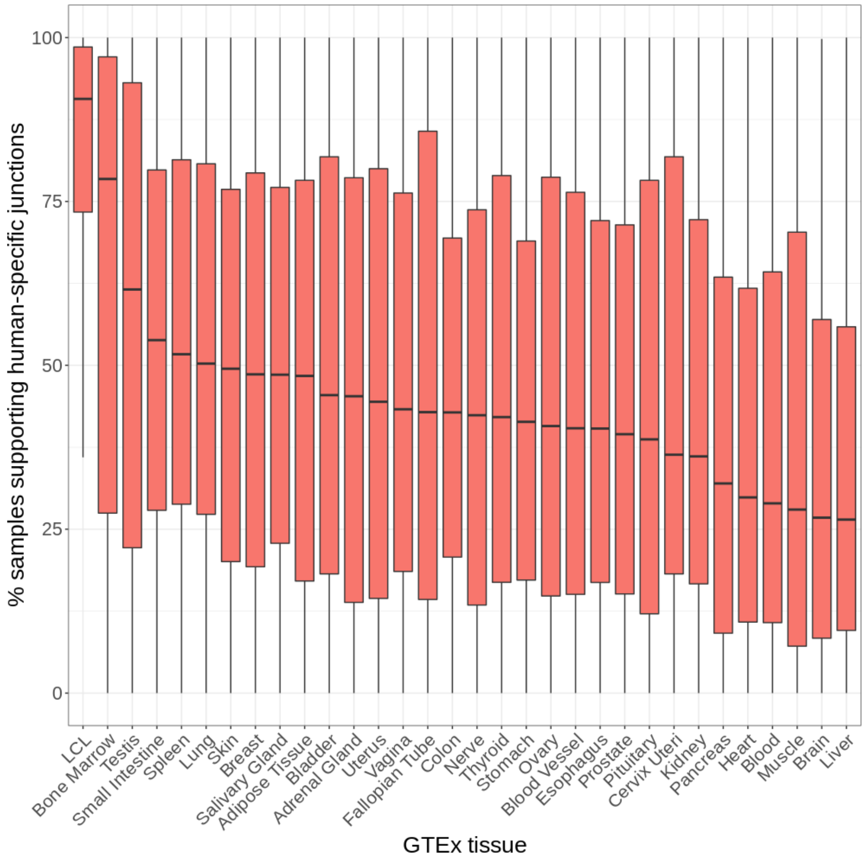

Supplemental Figure S20. Percentage of GTEx samples in each human tissue that show supporting reads for human-specific splice junctions. Human lymphoblastoid cell lines (LCLs) from GTEx are included as an independent group.

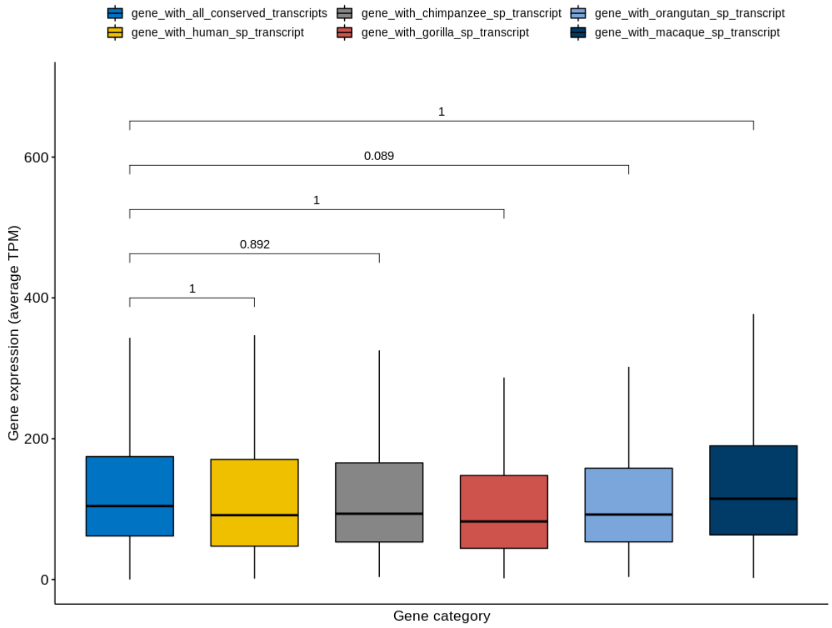

Supplemental Figure S21. Expression level (average TPM over 15 LCLs) for genes only expressing transcripts present in all 5 species, and for genes expressing species-specific transcripts. Statistical difference between groups was assessed using Student t-test (Two-sided) and p-values were adjusted using Holm method.

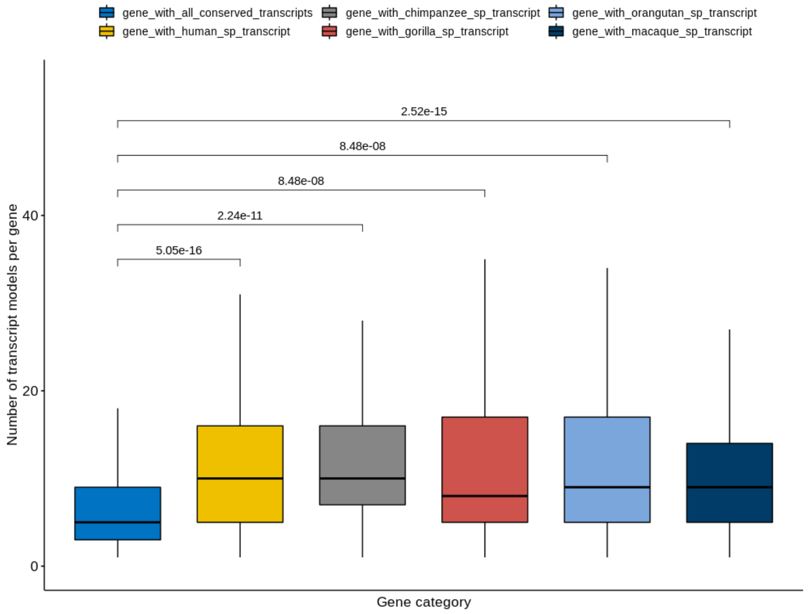

Supplemental Figure S22. Number of projected Iso-Seq transcript models for genes only expressing transcripts present in all 5 species and for genes expressing species-specific transcripts. Statistical difference between groups was assessed using Student t-test (Two-sided) and p-values were adjusted using Holm method.

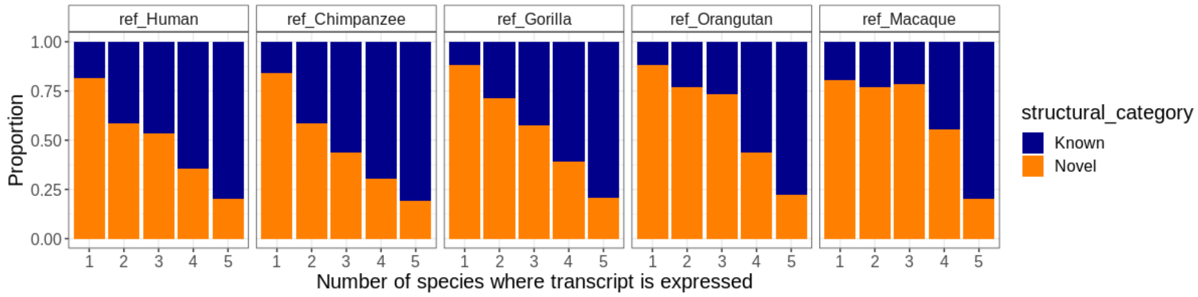

Supplemental Figure S23. Proportion of transcript models that are annotated ('Known') or unannotated ('Novel') in each species' reference annotation by expression conservation levels (number of species in which the transcript is expressed). Only transcripts with intra-species consistent patterns of expression (presence/absence) are shown.

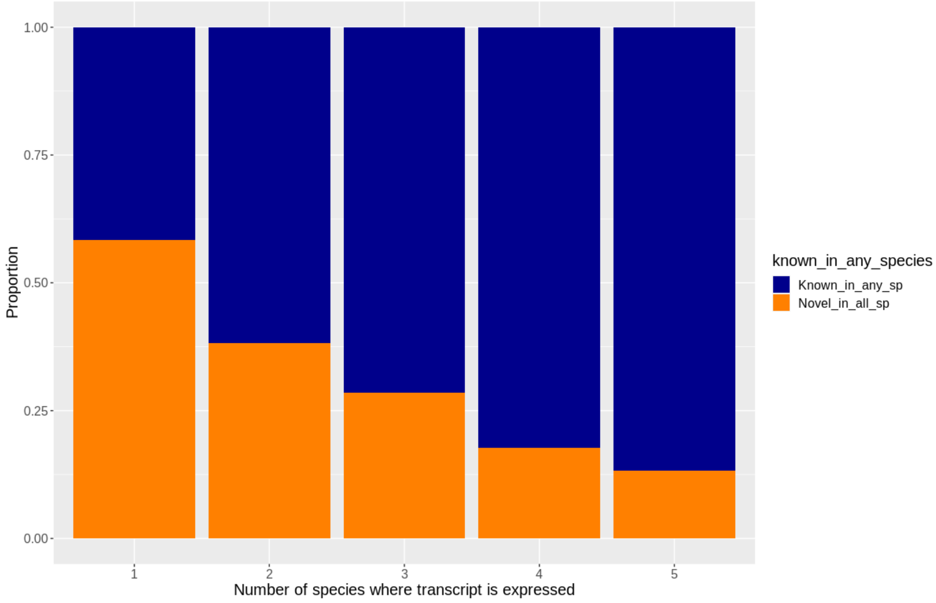

Supplemental Figure S24. Proportion of transcript models that are annotated in any species ('Known_in_any_sp') or unannotated in all five species ('Novel_in_all_sp') by expression conservation levels (number of species in which the transcript is expressed). Only transcripts with intra-species consistent patterns of expression (presence/absence) are shown.

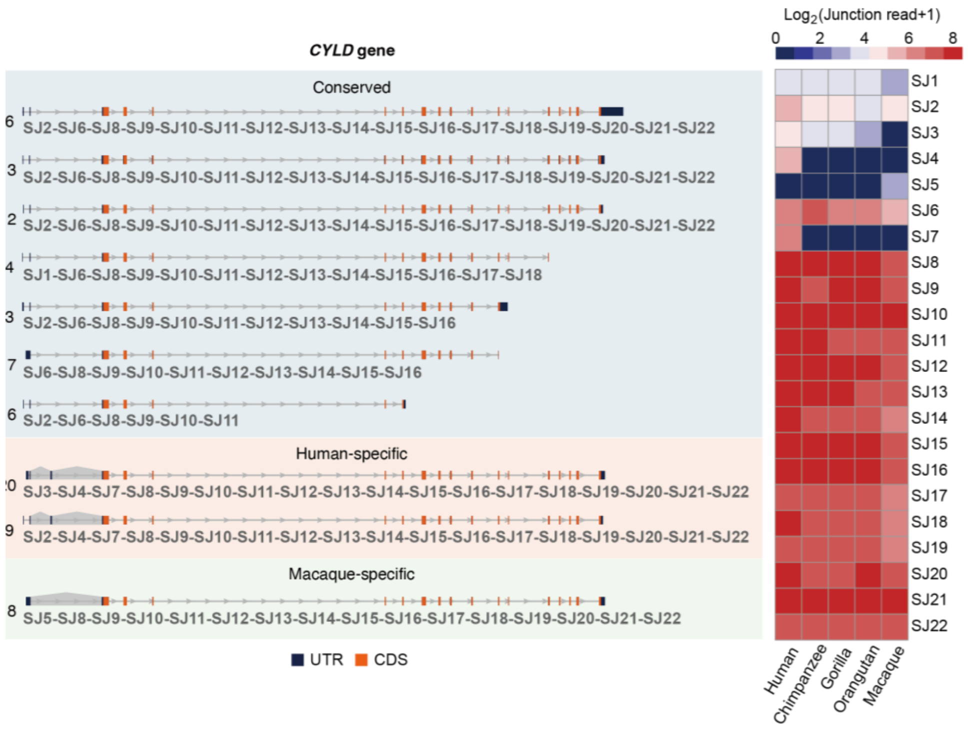

Supplemental Figure S25. *CYLD* transcript models and their splice junction (SJ) combinations are represented (left) alongside the RNA-seq junction reads found in each species (right). Splice junctions are numbered sequentially according to their location in the genome. The heatmap displays a color-gradient for $\log_2$(RNA-seq junction reads +1) in each splice junction (average number of reads per species). Grey shadows in transcript structures indicate species-specific splice junctions. Untranslated regions (UTRs) and coding sequences (CDS) are colored in blue and orange, respectively.

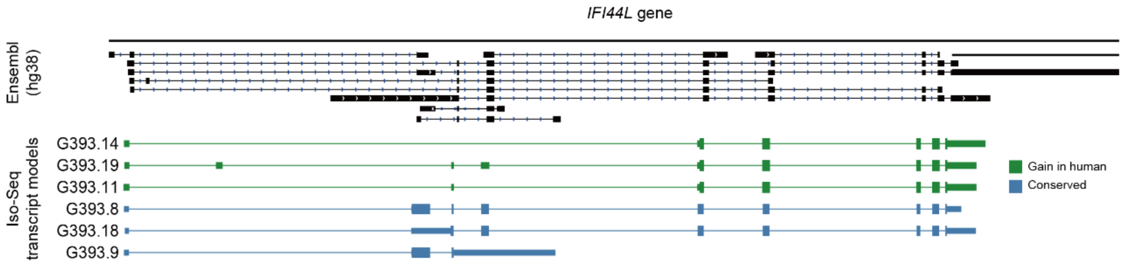

Supplemental Figure S26. Example of a gene, *IFI44L*, expressing human-specific and conserved transcripts. Ensembl annotation in hg38 assembly (black) is displayed (top) together with *IFI44L* Iso-Seq transcript models (bottom) that are human-specific (green) or expressed in all species (blue). In Iso-Seq transcript models, narrow blocks represent the untranslated region (UTR) and thick blocks indicate the coding region (CDS).

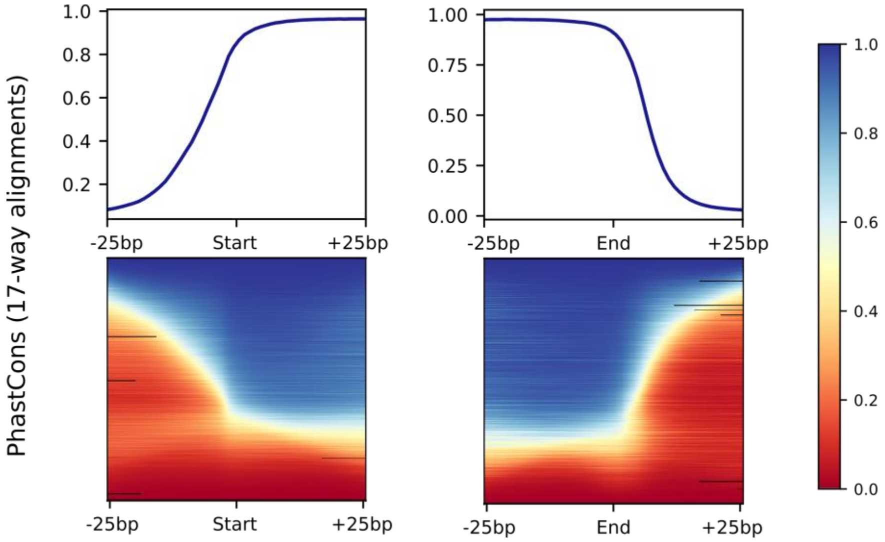

Supplemental Figure S27. PhastCons conservation scores in 3’ splice sites (‘Start’ of the exon) and 5’ splice sites (‘End’ of the exon) and their surrounding regions (± 25 base pairs from exon boundaries). For every single position (nucleotide), median conservation scores were calculated by deeptools (Ramírez et al. 2016) over a set of non-redundant exons (different in their genomic coordinates) present in the collapsed projected Iso-Seq models (hg38) (top). PhastCons conservation scores for every single position across these exons are represented as a color gradient (bottom) according to the color legend (right). Only exons that are 100-500 nucleotides long were kept for this representation (N=84,785 exons). Conservation scores were retrieved from 17-way alignments (UCSC).

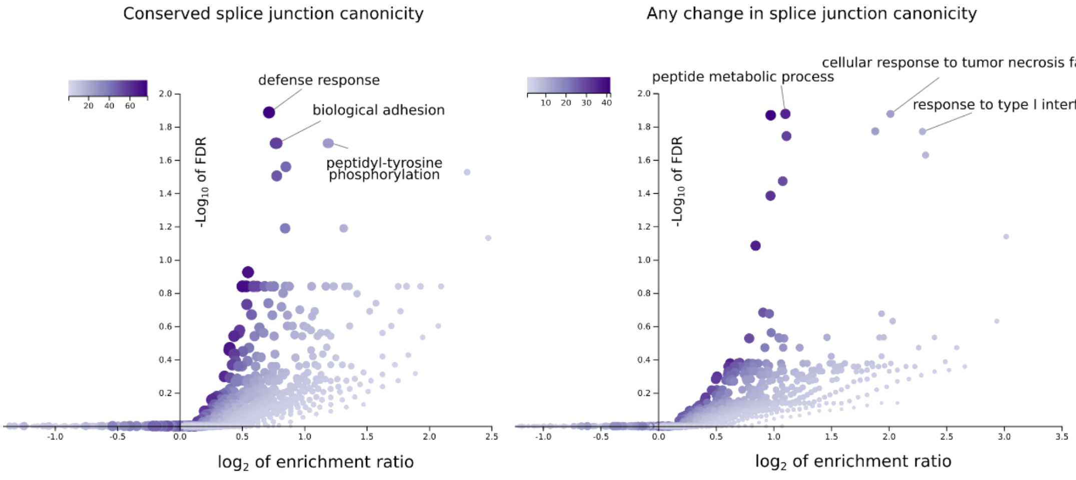

Supplemental Figure S28. Functional enrichment for genes expressing species-specific transcripts that arise from conserved splice site canonicity (left, N=627 genes) and not conserved splice site canonicity (right, N=280 genes) across primates. The enrichment was computed by over-representation analysis (ORA) using the Gene Ontology Biological Process database (affinity propagation clustering). False discovery rates (FDR) were adjusted using the Benjamini-Hochberg method (Benjamini et al. 2001). Color legend indicates the number of overlapping genes between the gene set under evaluation and the pathway gene set.

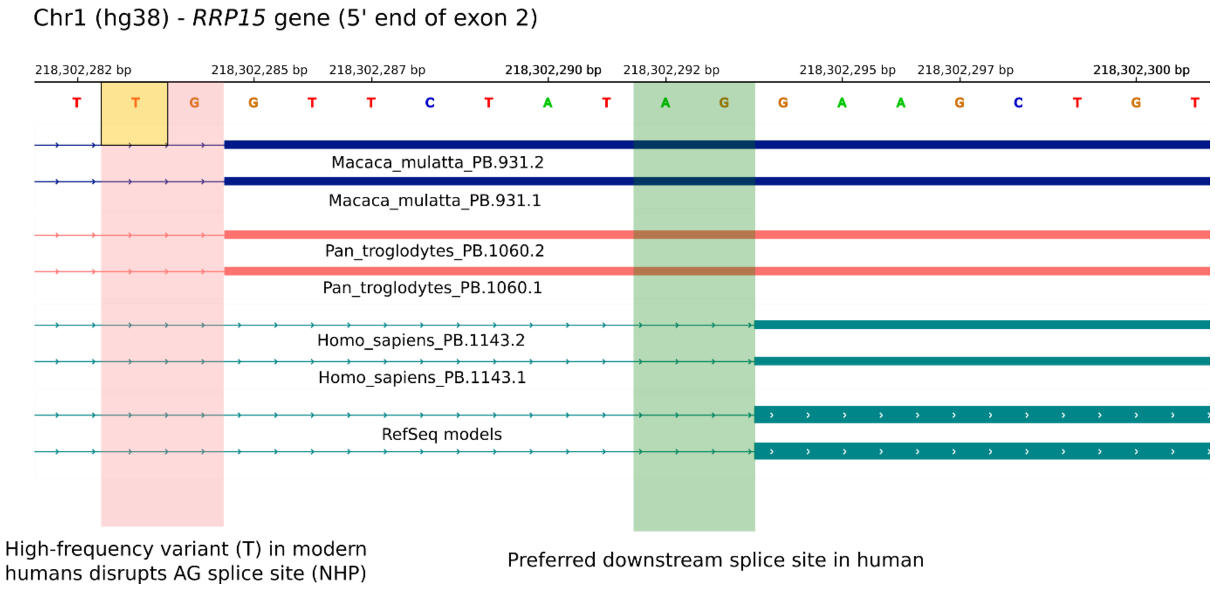

Supplemental Figure S29. Example of a gene**,** *RRP15*, with a human-specific splice site mutation altering the definition of the 5' boundary of *RRP15* exon 2. *Macaca mulatta* (blue) and *Pan troglodytes* (pink) Iso-Seq projected isoforms are shown together with *Homo sapiens* Iso-Seq isoforms and RefSeq models in hg38 assembly (green).

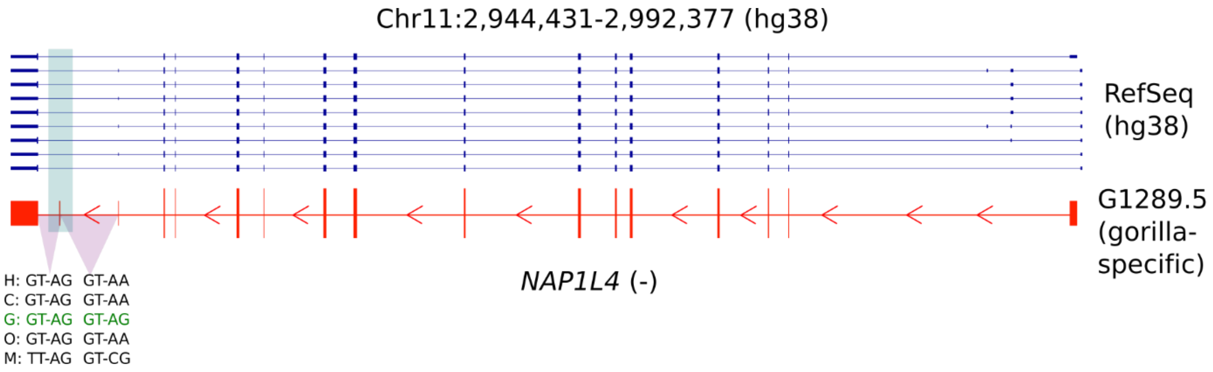

Supplemental Figure S30. Example of a gene, *NAP1L4*, with a gorilla-specific splice site mutation resulting in an exonization event. RefSeq has already annotated this gorilla-specific 3' UTR exon in gorGor6 assembly.

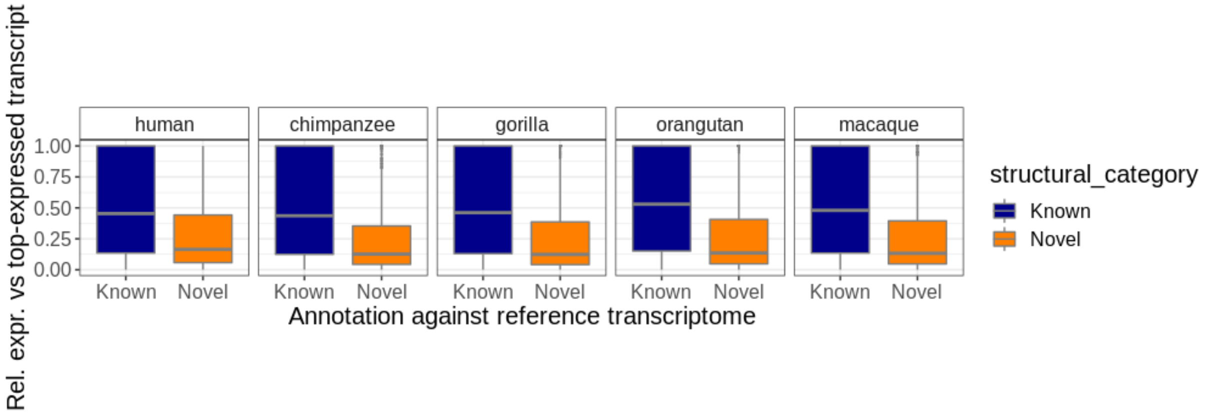

Supplemental Figure S31. Relative isoform expression *versus* expression of the top-expressed isoform for a given gene. Known transcripts (blue) show higher relative expression than novel transcripts (orange) in all species. Only expressed transcripts in a given species are shown.

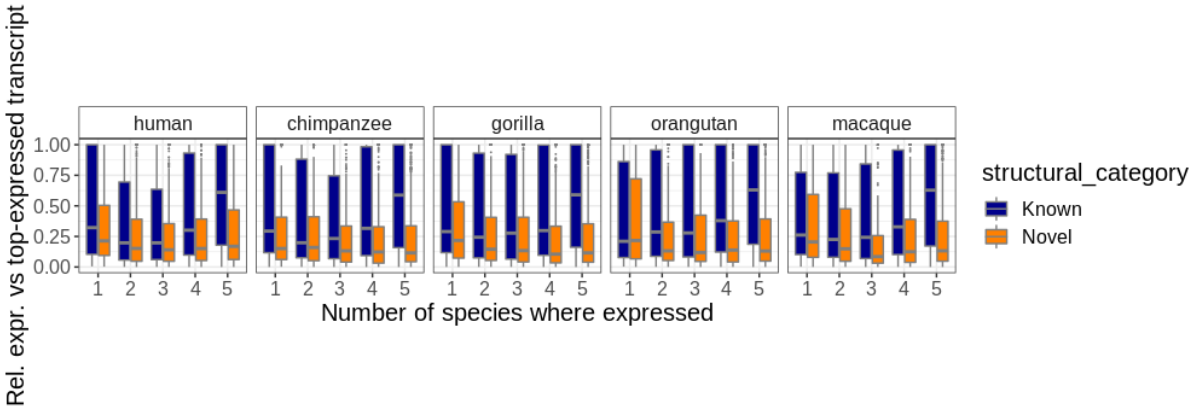

Supplemental Figure S32. Relative transcript expression *versus* expression of the top-expressed transcript for a given gene across conservation levels (number of species in which transcript is expressed). Only expressed transcripts in a given species are shown.

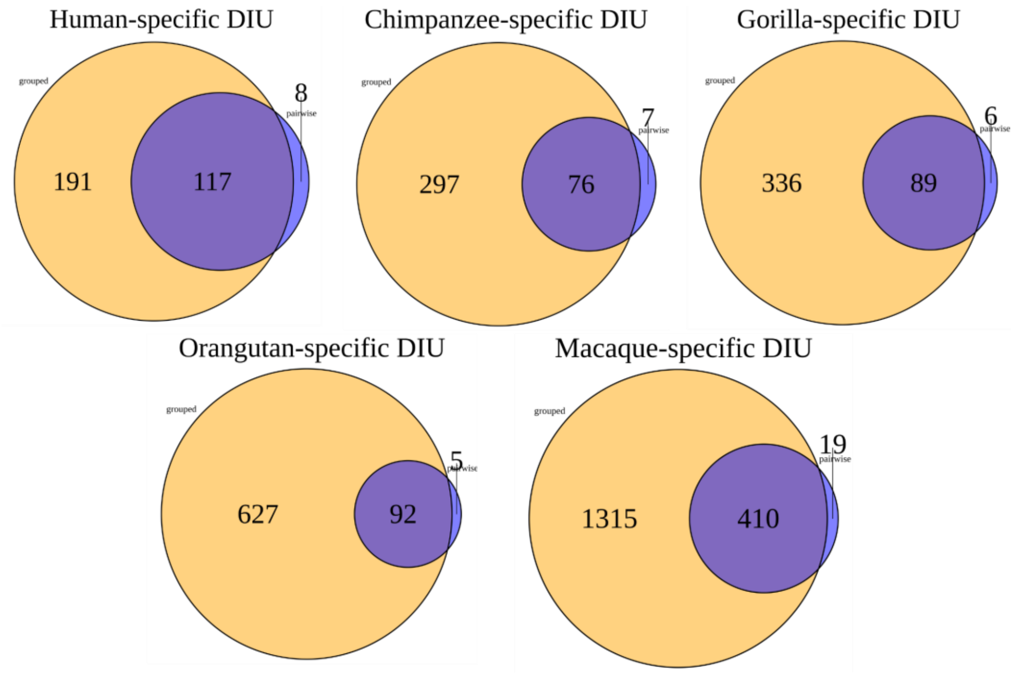

Supplemental Figure S33. Venn diagram showing the intersection of species-specific isoform usage changes detected by two different strategies. ‘Grouped’: isoform usage is compared in each species *versus* a group consisting of all the remaining species (yellow). ‘Pairwise’: isoform usage is compared in all pairwise combinations, where the isoform must display a significant usage change in a given species *versus* the others while the remaining species must show no significant usage differences between them (purple).

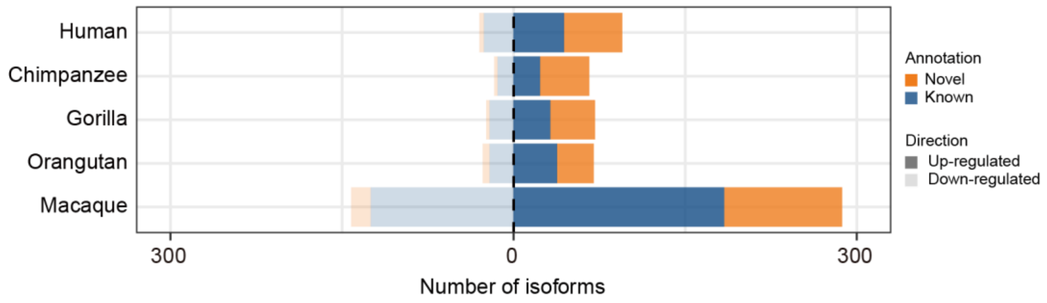

Supplemental Figure S34. Number of isoforms showing species-specific usage changes. Up-regulated (dark shadow) and down-regulated (light shadow) isoforms are classified into novel (orange) and known (blue) isoforms based on the human reference transcriptome (hg38 assembly).

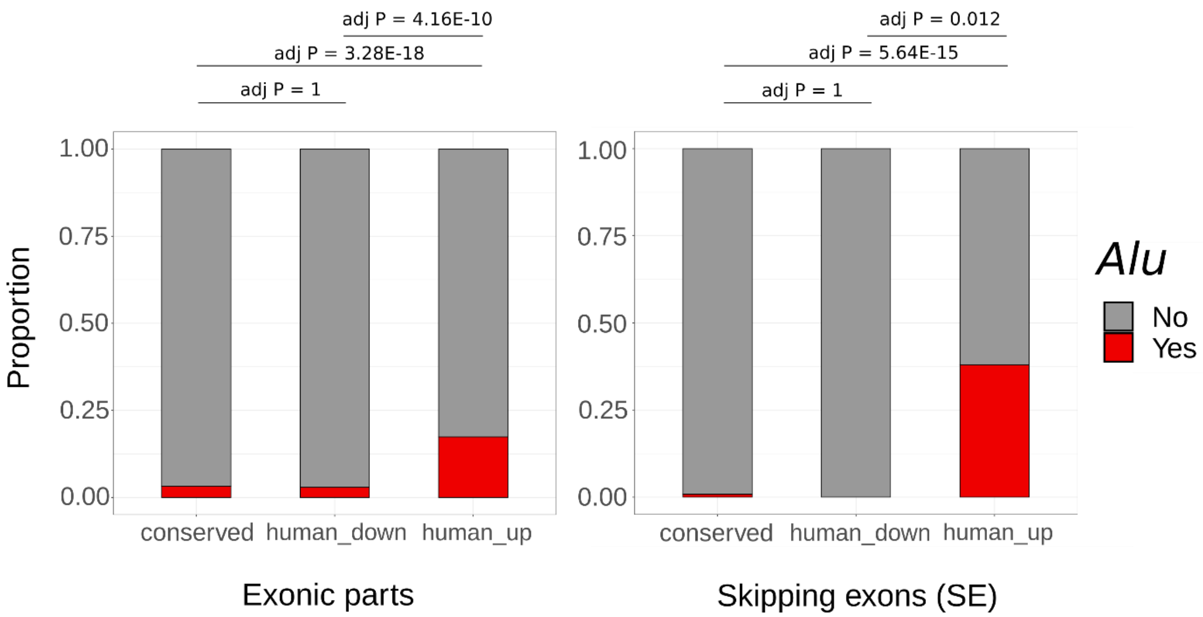

Supplemental Figure S35. Proportion of exonic parts intersecting *Alu* elements (conserved usage: 'conserved'; human-specific down-regulated: 'human_down'; human-specific up-regulated: 'human_up'). All exonic parts are shown to the left while skipping exons (SE) are displayed to the right. *Alu* elements were retrieved from hg38 repeatMasker track (UCSC). Statistical significance in the difference in proportions across groups was evaluated using Fisher's exact test. P-values were adjusted using the Bonferroni method.

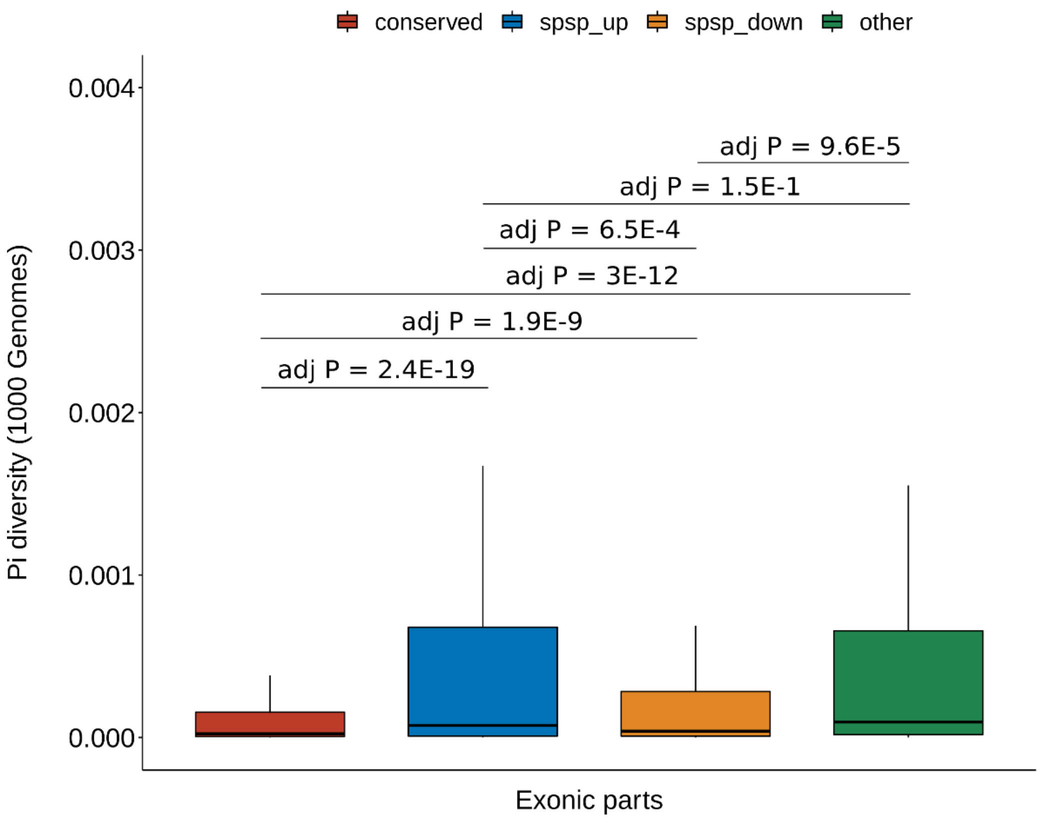

Supplemental Figure S36. Average nucleotide diversity (pi) in human populations for exonic parts with conserved usage (‘conserved’, N=95,453; red), exonic parts with species-specific up-regulation (‘spsp_up’, N=941; blue) and down-regulation (‘spsp_down’, N=1,340; orange), and exonic parts showing other usage changes (‘other’, N=277; green). ‘other’ usage changes correspond to usage differences between groups of species (*e.g.,* any 2 species show differential usage *versus* the remaining 3 species). Pi diversity estimates were calculated from the 1000 Genomes data mapped against hg38. Only exonic parts longer than 5 bases with all nucleotides included as passed bases according to 1000 Genomes strict mask are shown. Statistical significance of the difference across groups was assessed by the Wilcoxon test. P-values were adjusted using the Holm method.

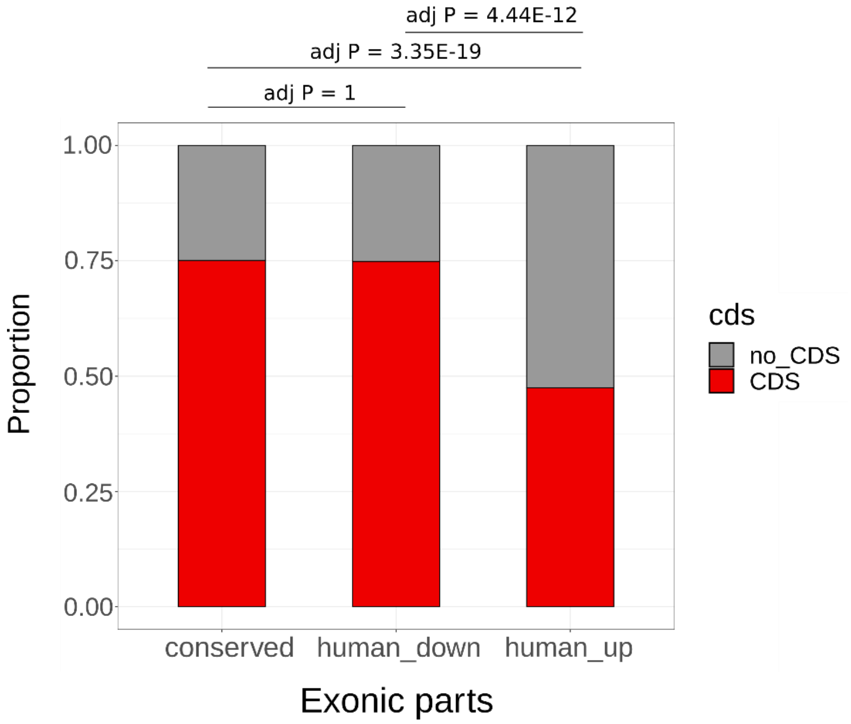

Supplemental Figure S37. Proportion of exonic parts intersecting coding regions (CDS) predicted from Iso-Seq projected models (conserved usage: ‘conserved’; human-specific down-regulated: ‘human_down’; human-specific up-regulated: ‘human_up’). Statistical significance in the difference in proportions across groups was evaluated using Fisher’s exact test. P-values were adjusted using the Bonferroni method.

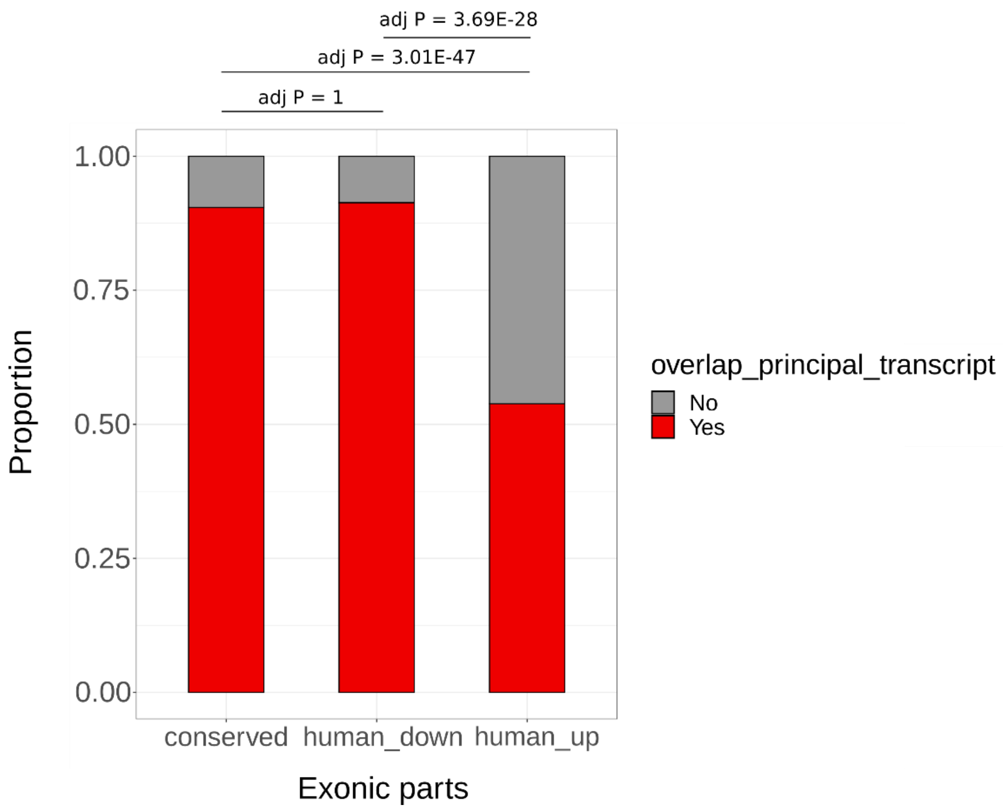

Supplemental Figure S38. Proportion of exonic parts intersecting regions included in APPRIS principal transcripts (hg38) (conserved usage: ‘conserved’; human-specific down-regulated: ‘human_down’; human-specific up-regulated: ‘human_up’). Statistical significance in the difference in proportions across groups was evaluated using Fisher’s exact test. P-values were adjusted using the Bonferroni method.

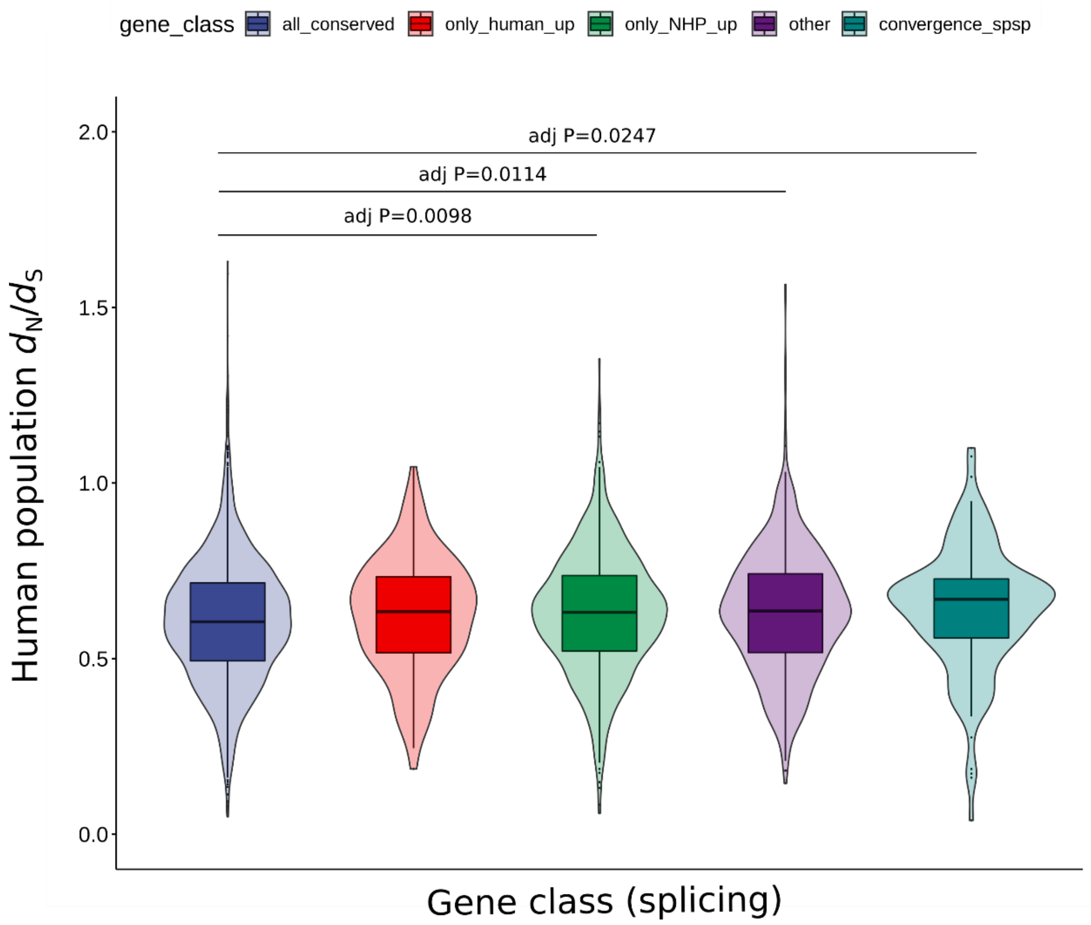

Supplemental Figure S39. Ratio of nonsynonymous to synonymous substitutions (d_N/d_S) in human populations for each of the gene classes according to their splicing and usage patterns. Statistical significance for the pairwise comparisons was obtained using the Dwass-Steel-Critchlow-Fligner all-pairs test. P-values were adjusted using the single-step method. Only adjusted p-values lower than 0.05 are shown.

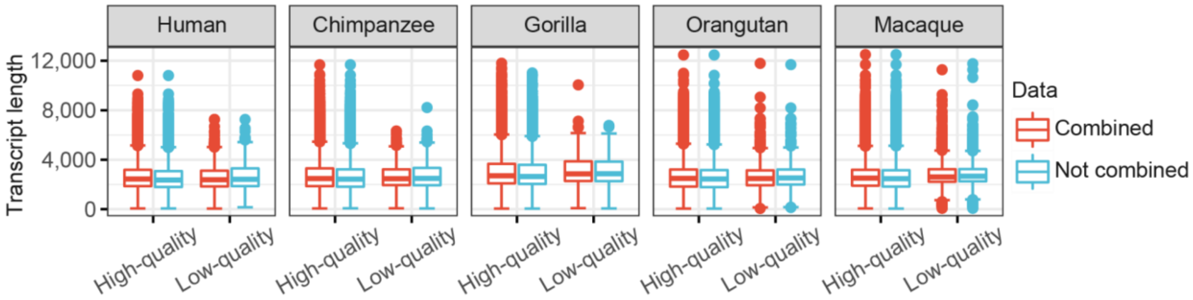

Supplemental Figure S40. Length distribution of Isoseq3 transcript sequences combining all subreads from the same species ('Combined') *versus* independent processing of subreads from different libraries and subsequent isoform merging per species ('Not combined', alternative strategy).

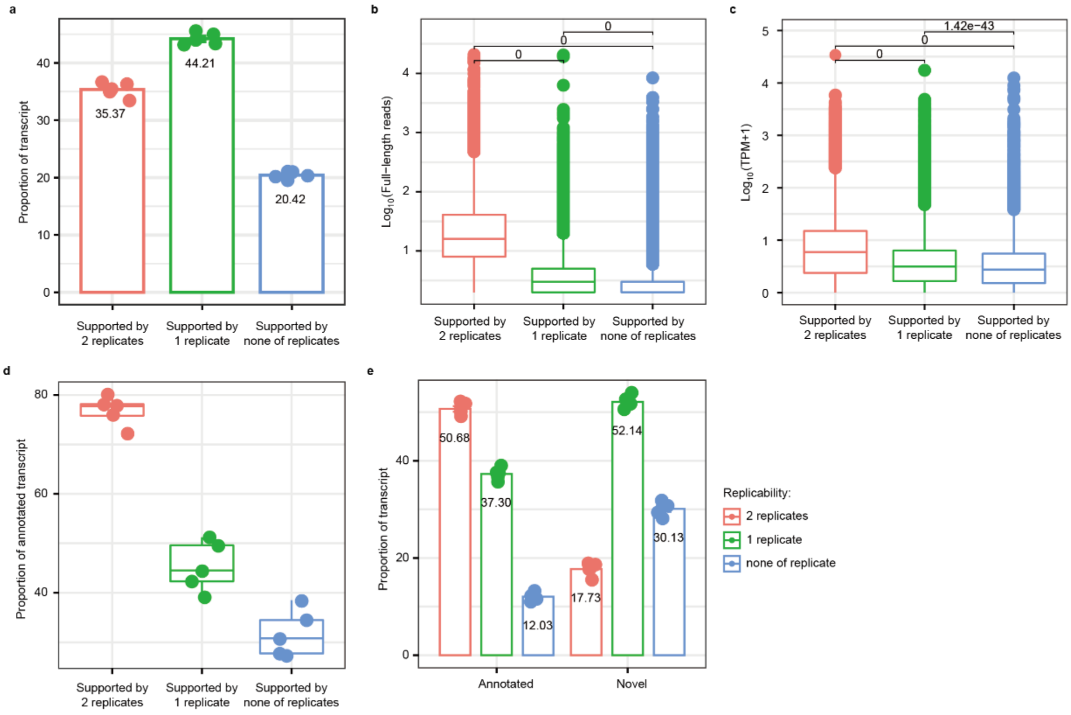

Supplemental Figure S41. a) Percentage of Iso-Seq transcripts (SQANTI-filtered) that were supported in 2 LCLs, 1 LCL or unsupported after independent Iso-Seq re-processing for each LCL. **b)** Long read-based transcript expression for each Iso-Seq replicability class. **c)** Short read-based transcript expression for each Iso-Seq replicability class. **d)** Percentage of annotated transcripts in each Iso-Seq replicability class. **e)** Percentage of each Iso-Seq replicability class in annotated and novel transcripts.

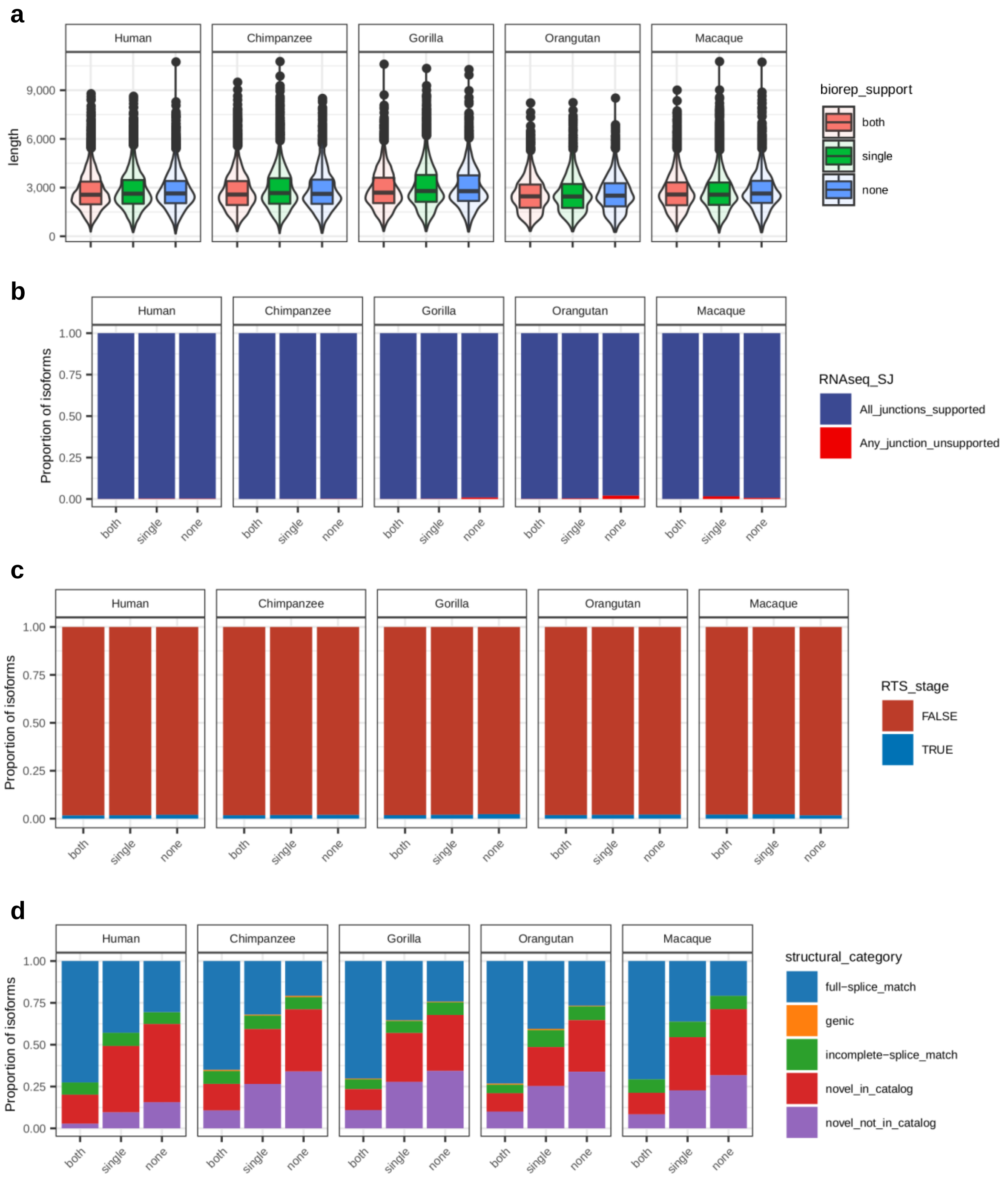

Supplemental Figure S42. Isoform quality descriptors by Iso-Seq replicability classes (‘both’: isoform was independently identified in two LCLs for that species; ‘single’: isoform was independently identified in one LCL for that species; ‘none’: isoform was identified after combining Iso-Seq subreads from the two LCLs per species, but not in independent Iso-Seq reprocessing of each LCL). Transcript length **(a)**, splice junction coverage by RNA-seq **(b)**, RTS switching **(c)** and distribution of isoform structural categories **(d)** are shown for the set of isoforms produced by 1:1 orthologous genes in the 5 primate species.

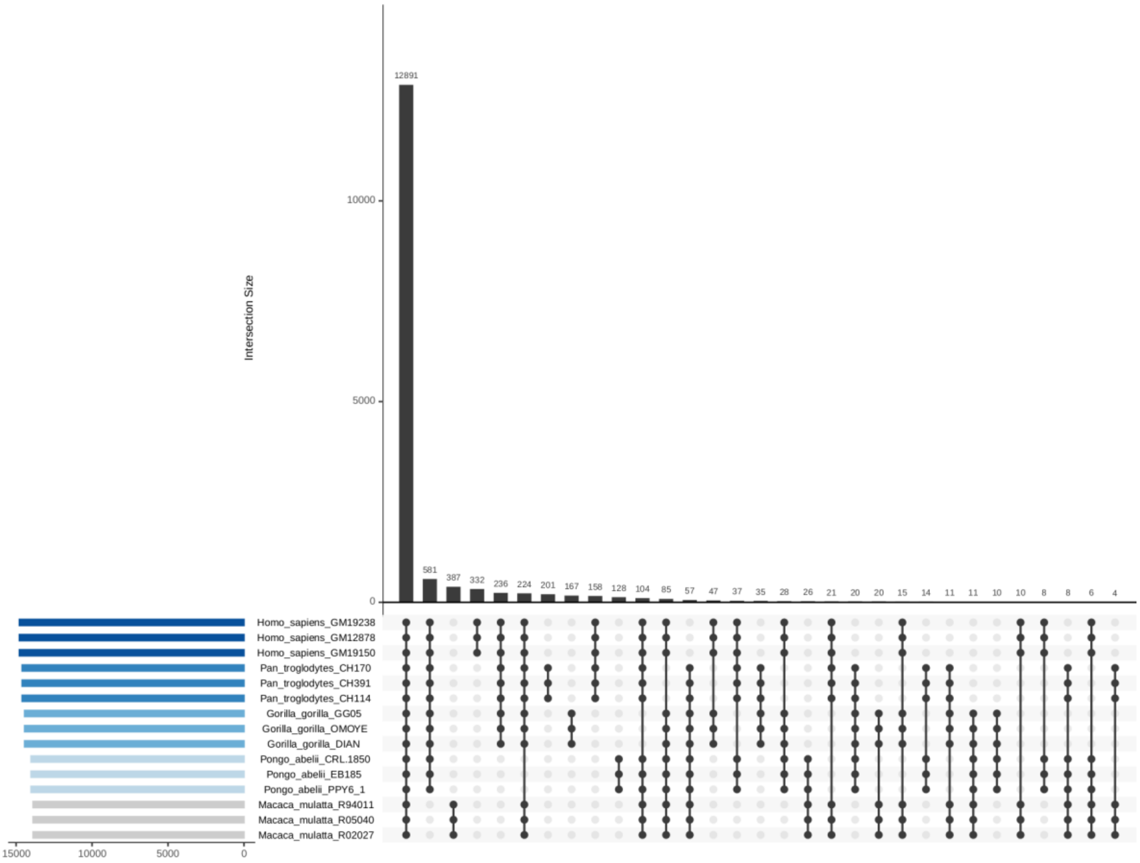
Intersection Size
12891
581 387 332 236 224 201 167 158 128 104 85 57 47 37 35 28 26 21 20 20 15 14 11 11 10 10 8 8 6 4
10000
5000
0
Homo_sapiens_GM19238
Homo_sapiens_GM12878
Homo_sapiens_GM19150
Pan_troglodytes_CH170
Pan_troglodytes_CH391
Pan_troglodytes_CH114
Gorilla_gorilla_GG05
Gorilla_gorilla_OMOYE
Gorilla_gorilla_DIAN
Pongo_abelii_CRL.1850
Pongo_abelii_EB185
Pongo_abelii_PPY6_1
Macaca_mulatta_R94011
Macaca_mulatta_R05040
Macaca_mulatta_R02027
15000
10000
5000
0

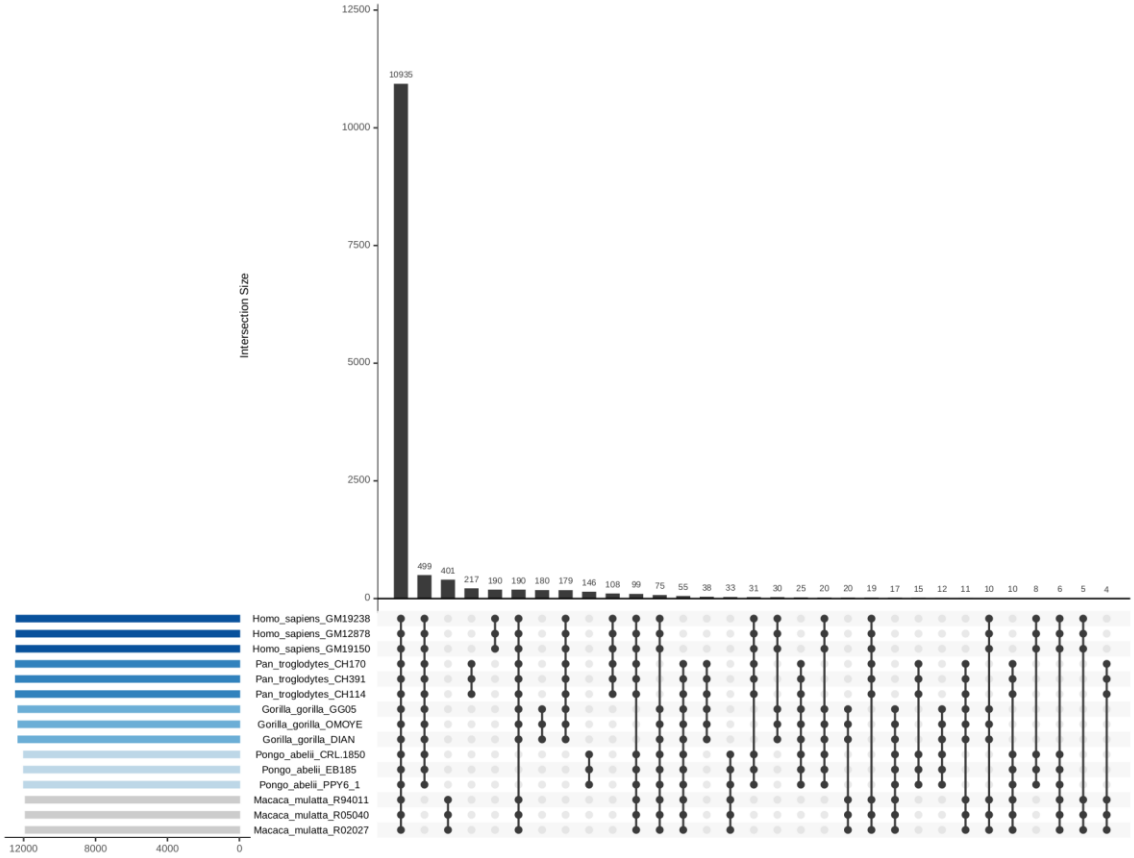
Intersection Size
12500
10935
10000
7500
5000
2500
0
499 401 217 190 190 180 179 146 108 99 75 55 38 33 31 30 25 20 20 19 17 15 12 11 10 10 8 6 5 4
Homo_sapiens_GM19238
Homo_sapiens_GM12878
Homo_sapiens_GM19150
Pan_troglodytes_CH170
Pan_troglodytes_CH391
Pan_troglodytes_CH114
Gorilla_gorilla_GG05
Gorilla_gorilla_OMOYE
Gorilla_gorilla_DIAN
Pongo_abelii_CRL.1850
Pongo_abelii_EB185
Pongo_abelii_PPY6_1
Macaca_mulatta_R94011
Macaca_mulatta_R05040
Macaca_mulatta_R02027
12000
8000
4000
0

Supplemental Figure S43. Upset plot showing transcript expression presence/absence when considering a 0 TPM cutoff (top panel) and setting all isoforms with TPM<0.1 as non-expressed (bottom panel). Only isoforms showing intra-species consistency in expression/absence of expression patterns are included in this plot.

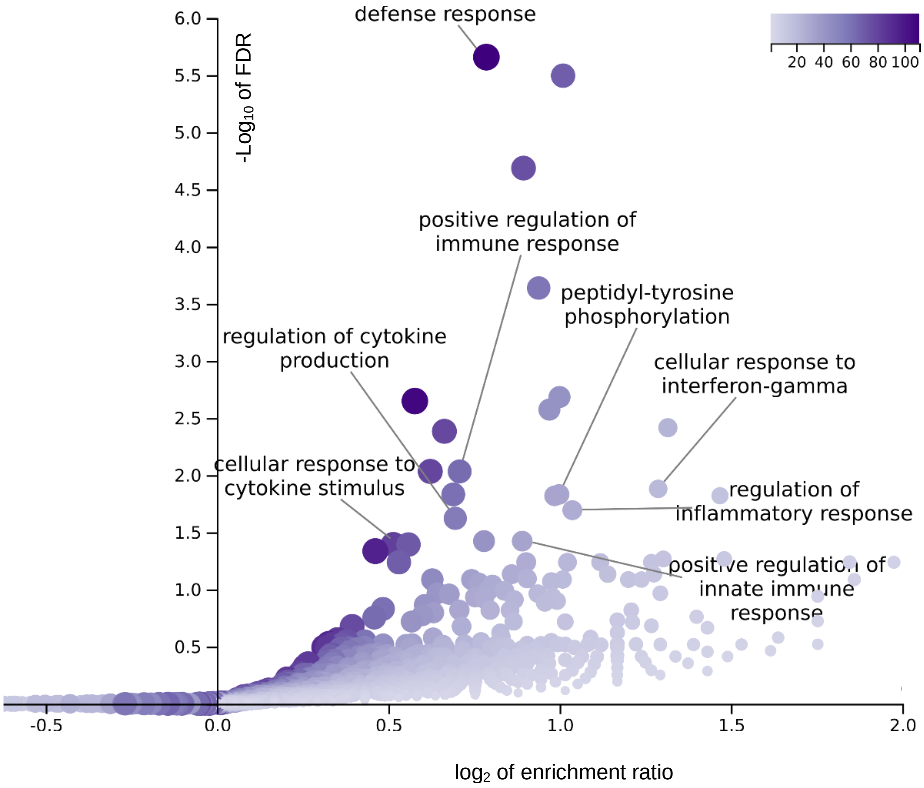

Supplemental Figure S44. Functional enrichment for genes displaying species-specific isoform innovations (N=877 genes) considering transcripts with TPM<0.1 as non-expressed. Over-representation analysis (ORA) was conducted using the Gene Ontology Biological Process database (affinity propagation clustering). False discovery rates (FDR) were adjusted using the Benjamini-Hochberg method. Color legend indicates the number of overlapping genes between the gene set under evaluation and the pathway gene set.

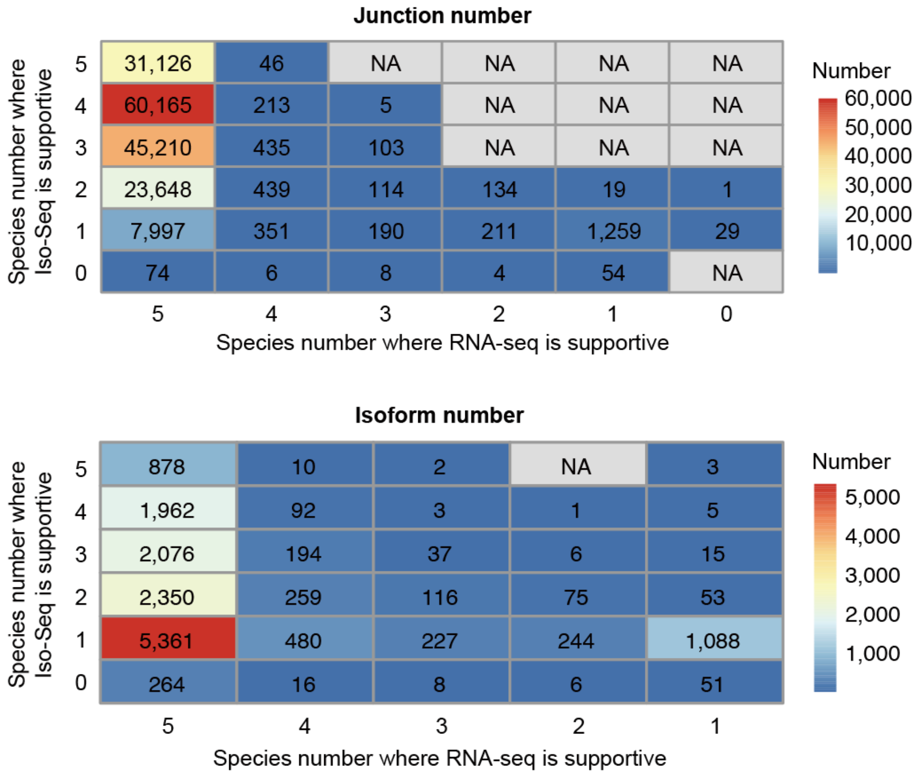

Supplemental Figure S45. Junction support (top) and isoform expression (bottom) conservation level calculated from RNA-seq and Iso-Seq across 5 primate species.

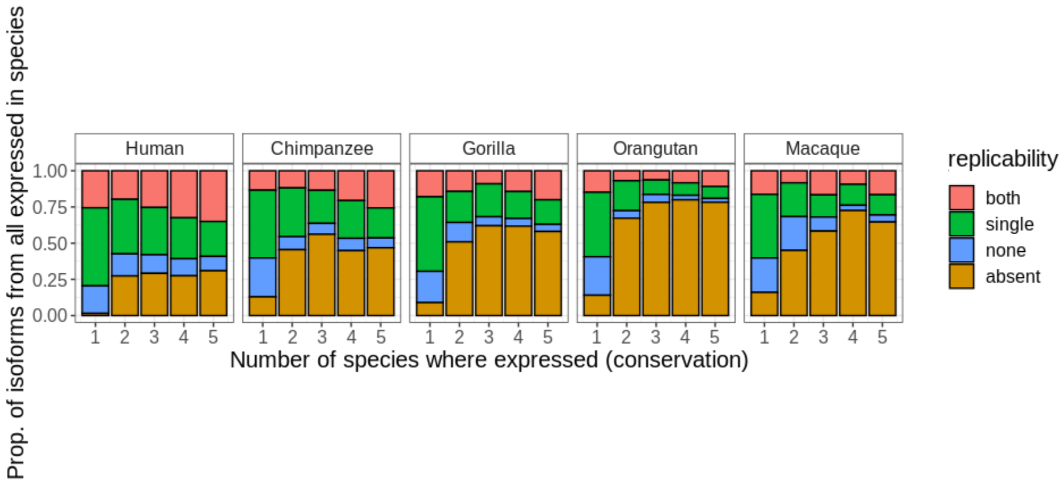

Supplemental Figure S46. Distribution of Iso-Seq replicability classes by conservation level in the expressed isoforms in each species. Only transcripts showing intra-species consistency in their patterns of expression presence/absence are shown. ‘both’: isoform was captured in 2 LCLs from a given species; ‘single’: isoform was captured in 1 LCL from a given species; ‘none’: isoform was captured combining subreads from the same species, but not in independent Iso-Seq replicate re-processing; ‘absent’: isoform was captured in any other species’ Iso-Seq.

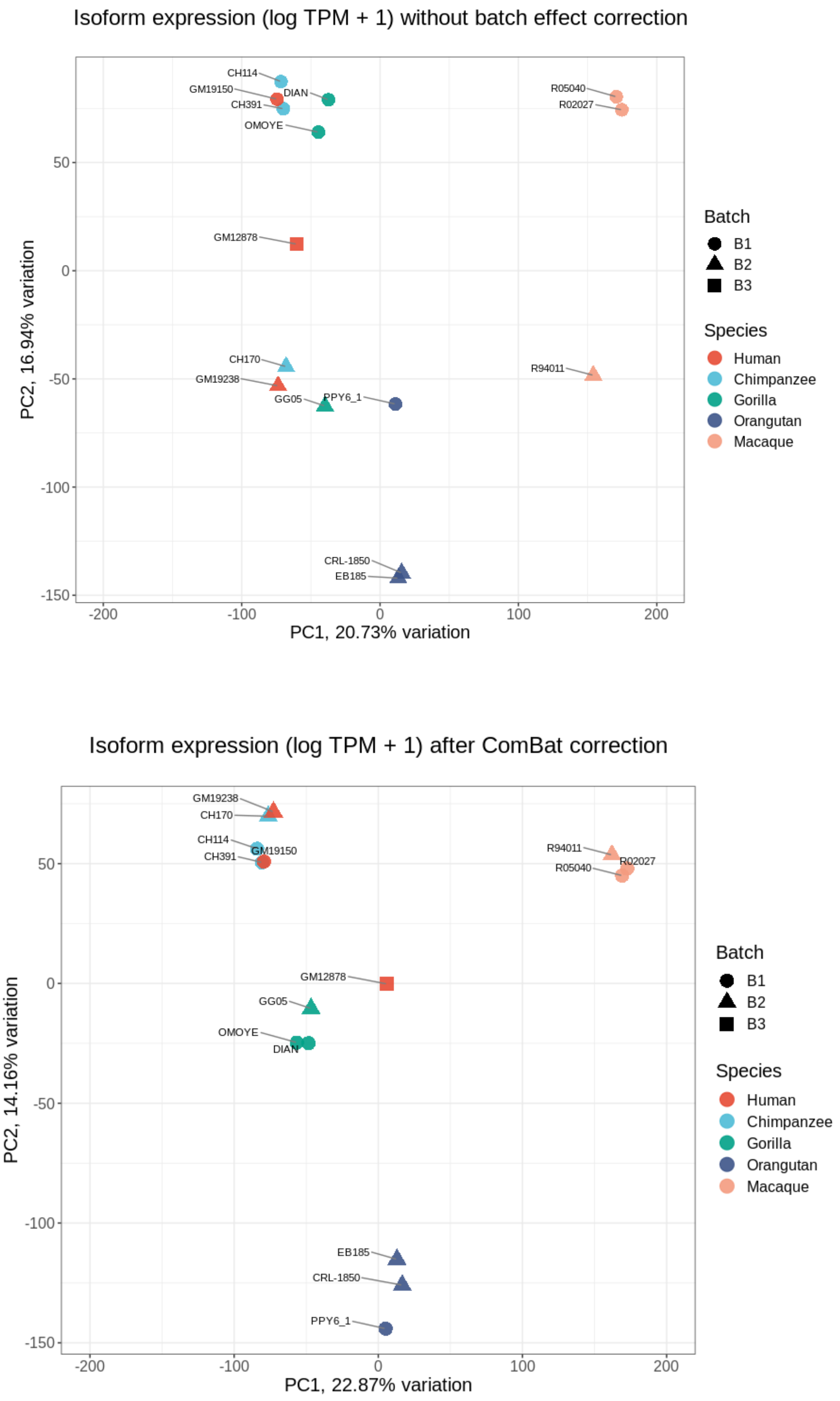

Supplemental Figure S47. Principal component analysis (PCA, PC1 vs PC2) plot for transcript TPM values (base-e logarithm) before (top) and after (bottom) batch effect correction using ComBat. RNA-seq experimental batches (B1, B2, B3) are coded by different shapes, and species are color-coded.

Supplemental Tables

Supplemental Table S1. Information for the panel of lymphoblastoid cell lines used in this study.

Sample name	ID in García-Pérez et al (2021)	Species	Iso-Seq	RNA-seq	MS/MS	Source
GM12878	H1	*Homo sapiens*	This study	SRA SRR998197 and SRR998198	This study	Coriell Institute (comercial)
GM19150	H2	*Homo sapiens*	This study	García-Pérez et al (2021)	This study	Coriell Institute (comercial)
GM19238	N/A	*Homo sapiens*	N/A	This study	This study	Coriell Institute (comercial)
CH114	C1	*Pan troglodytes*	This study	García-Pérez et al (2021)	N/A	Antoine Blancher
CH391	C2	*Pan troglodytes*	This study	García-Pérez et al (2021)	N/A	Antoine Blancher
CH322	N/A	*Pan troglodytes*	N/A	This study	This study	Antoine Blancher
CH507	N/A	*Pan troglodytes*	N/A	This study	This study	Antoine Blancher
CH170	N/A	*Pan troglodytes*	N/A	This study	This study	Antoine Blancher
DIAN	G1	*Gorilla gorilla*	This study	García-Pérez et al (2021)	This study	Antoine Blancher
OMOYE	G2	*Gorilla gorilla*	This study	García-Pérez et al (2021)	This study	Antoine Blancher
GG05	N/A	*Gorilla gorilla*	N/A	This study	This study	Aurora Ruiz
PPY6	O2	*Pongo pygmaeus*	This study (2 isogenic cultures)	García-Pérez et al (2021)	This study	Antoine Blancher
CRL-1850 (PUTI)	N/A	*Pongo pygmaeus*	N/A	This study	This study	Chris Tyler-Smith
EB185	N/A	*Pongo pygmaeus*	N/A	This study	This study	Sigma-Aldrich (comercial)
R02027	M1	*Macaca mulatta*	This study	García-Pérez et al (2021)	This study	Gaby Dioxiadis
R05040	M2	*Macaca mulatta*	This study	García-Pérez et al (2021)	This study	Gaby Dioxiadis
R94011	N/A	*Macaca mulatta*	N/A	This study	This study	Gaby Dioxiadis

Supplemental Table S2. Comparison of long-read transcriptomes from this study with currently existing primate Iso-Seq datasets. ROI: read of insert; CCS: circular consensus sequence; FLNC: full-length non-chimeric reads; HQ: high-quality.

PMID and reference	Species	Tissues	Type	Subreads	ROI/CCS	FLNC	Processed alignments	HQ transcript isoform
This study	Human, Chimpanzee, Gorilla, Orangutan and Macaque	LCLs	Maximum	41,598,084	2,901,238	2,137,334	104,486	50,769
			Minimum	34,749,474	1,959,837	1,213,522	64,693	13,766
33953399 (Mao et al. 2021)	Bonobo	Induced pluripotent stem cell derived neuronal progenitor cell lines	Total	-	-	867,690	-	-
31530812 (He et al. 2019)	Chinese rhesus macaque	Combined 10 tissues (heart, liver, spleen, lung, kidney, muscle, brain, epencephala, testicle and stomach)	Total	-	-	2,468,473	-	-
29880660 (Kronenberg et al. 2018)	Human, Chimpanzee, Gorilla and Orangutan	Induced pluripotent stem cells	Maximum	-	-	881,801	-	-
			Minimum	-	-	528,145	-	-
28957512 (Zhang et al. 2017)	Human and Macaque	Cerebellum	Maximum	20,107,093	1,893,217	-	589,704	-
			Minimum	8,534,738	765,100	-	184,712	-

Supplemental Table S3. Statistics of PacBio Iso-Seq and Illumina RNA-seq data production in 5 primate species. CCS: circular consensus sequence; FLNC: full-length non-chimeric reads; HQ: high-quality.

Data production		Human	Chimpanzee	Gorilla	Orangutan	Macaque
Iso-Seq	Subreads	41,279,111	41,598,084	34,749,474	39,453,894	38,893,358
	CCS	2,901,238	2,478,756	2,144,402	1,959,837	2,226,668
	FLNC	2,137,334	1,832,946	1,564,808	1,213,522	1,458,642
	Polished transcripts	104,486	98,602	86,768	64,693	83,262
	Non-redundant transcripts	63,577	55,417	46,191	34,124	48,247
	HQ transcripts after SQANTI filtering	50,769	34,871	26,003	13,766	23,201
	Non-redundant junctions in HQ transcripts	113,001	83,193	68,521	39,798	65,076
RNA-seq	Reads	368,075,841	590,075,508	539,000,137	288,468,437	529,554,982
	Non-redundant junctions	514,144	678,985	582,798	470,534	534,247

Supplemental Table S4. Percentage of transcripts remaining after each filtering/collapsing step during Iso-Seq data processing for 5 primate species. CCS: circular consensus sequence; FLNC: full-length non-chimeric reads.

Conversion	Human	Chimpanzee	Gorilla	Orangutan	Macaque
CCS → FLNC	73.67	73.95	72.97	61.92	65.51
FLNC → Polished transcript	4.89	5.38	5.54	5.33	5.71
Polished transcript → Non-redundant transcript	60.85	56.20	53.24	52.75	57.95
Non-redundant transcript → High-quality transcript	79.85	62.92	56.29	40.34	48.09

Supplemental Table S5. Number of Iso-Seq isoforms in each SQANTI structural category according to Ensembl models (V91). Only isoforms passing SQANTI QC (not artifacts) are shown. FSM: full splice match; ISM: incomplete splice match; NIC: novel in catalog; NNC: novel not in catalog. A detailed description of SQANTI structural categories can be found in Tardaguila et al. (2018).

Species	FSM	ISM	NIC	NNC	antisense	fusion	genic	genic intron	intergenic
Human	25,165	3,625	16,647	3,831	184	51	465	543	258
Chimpanzee	13,267	2,454	9,735	7,055	244	24	726	480	886
Gorilla	10,889	1,656	6,343	5,245	190	17	530	293	840
Orangutan	6,371	1,010	2,682	2,515	110	11	294	167	606
Macaque	9,257	1,755	6,056	4,047	168	23	535	412	948

Supplemental Table S6. Number of alternative splicing events detected in Iso-Seq transcriptomes. SE: skipping exons; RI: retained introns; A5SS: alternative 5' splice sites; A3SS: alternative 3' splice sites; MX: mutually exclusive exons.

Species	Total	SE	RI	A5SS	A3SS	MX
Human	16,337	6,401	4,062	2,845	2,658	371
Chimpanzee	10,371	4,233	2,529	1,650	1,716	243
Gorilla	7,312	2,892	1,728	1,307	1,241	144
Orangutan	3,015	1,280	622	586	462	65
Macaque	6,144	2,466	1,506	1,048	994	130

Supplemental Table S7. Number of detected peptides per species according to their presence in the simulated digestion of Iso-Seq isoforms projected to each genome (plausible in genome), UniProt and RefSeq reference proteomes. Detected peptides must satisfy FDR<5%, Mascot IonScore > 20, reporter ion intensity signal (abundance) per sample > 50 (in any sample of a given species), and species-wise median ratio of sample abundance to pool abundance > 0.6 (excluding matches to common contaminant sequences and peptides arising from more than 1 tryptic miscleavage). The numbers correspond to distinct tryptic peptides considering isoleucine and leucine (I/L) as indistinguishable by mass spectrometry.

Species	Detected peptides	Detected peptides and plausible in genome	Novel peptides in UniProt and plausible in genome	Novel peptides in RefSeq and plausible in genome
Human	24,308	24,180	22	25
Chimpanzee	25,158	24,733	320	98
Gorilla	25,577	24,409	882	94
Orangutan	25,741	25,205	1,590	225
Macaque	23,743	23,299	207	75

Supplemental Table S8. Functional enrichment for genes displaying species-specific transcript gains (over-representation analysis using Gene Ontology Biological Process database in WebGestalt, FDR ≤ 0.05).

Gene Set	Description	Size	Expect	Enrichment ratio	P-value	FDR
GO:0006952	defense response	434	64.315	1.6481	2.32E-08	0.00008551
GO:0045087	innate immune response	270	40.012	1.7495	6.90E-07	0.0010283
GO:0031347	regulation of defense response	231	34.232	1.8112	8.68E-07	0.0010283
GO:0018108	peptidyl-tyrosine phosphorylation	109	16.153	2.2287	1.1184E-06	0.0010283
GO:0018212	peptidyl-tyrosine modification	110	16.301	2.2084	1.4321E-06	0.0010535
GO:0034341	response to interferon-gamma	67	9.9288	2.4172	0.000015048	0.0092242
GO:0002252	immune effector process	456	67.575	1.465	0.000022508	0.011826
GO:0032101	regulation of response to external stimulus	203	30.083	1.7286	0.00002846	0.013085
GO:0031349	positive regulation of defense response	146	21.636	1.8488	0.000048233	0.016607

GO:0050776	regulation of immune response	325	48.162	1.5365	0.000052747	0.016607
GO:0045088	regulation of innate immune response	142	21.043	1.8533	0.000056417	0.016607
GO:0071346	cellular response to interferon-gamma	59	8.7433	2.4018	0.000057105	0.016607
GO:0002684	positive regulation of immune system process	337	49.94	1.5218	0.000058697	0.016607
GO:0050778	positive regulation of immune response	254	37.641	1.594	0.000093189	0.024482
GO:0050707	regulation of cytokine secretion	55	8.1505	2.3311	0.00020184	0.046466
GO:0019221	cytokine-mediated signaling pathway	235	34.825	1.5793	0.00023632	0.046466
GO:0002682	regulation of immune system process	477	70.687	1.3864	0.0002377	0.046466
GO:0034340	response to type I interferon	43	6.3722	2.5109	0.00024081	0.046466
GO:0060333	interferon-gamma-mediated signaling pathway	39	5.7795	2.5954	0.00024577	0.046466
GO:0001816	cytokine production	252	37.344	1.5531	0.00025768	0.046466
GO:0001817	regulation of cytokine production	226	33.491	1.5825	0.00029057	0.046466
GO:0022610	biological adhesion	313	46.384	1.4876	0.00026845	0.046466
GO:0071345	cellular response to cytokine stimulus	353	52.312	1.4528	0.00028704	0.046466

Supplemental Table S9. Functional enrichment for genes expressing transcripts which show a change in junction canonicity in any species (over-representation analysis using Panther pathways database in WebGestalt, FDR ≤ 0.05).

Gene Set	Description	Size	Expect	Enrichment ratio	P-value	FDR
P00010	B cell activation	47	6.0893	2.4633	0.00038484	0.01917
P00053	T cell activation	44	5.7006	2.4559	0.00063901	0.01917
P00038	JAK/STAT signaling pathway	11	1.4252	4.2101	0.0011021	0.022041

Supplemental Table S10. Functional enrichment for genes displaying species-specific exon usage changes (over-representation analysis using Gene Ontology Biological Process database in WebGestalt, FDR ≤ 0.05).

Gene Set	Description	Size	Expect	Enrichment Ratio	P-value	FDR
GO:0033365	protein localization to organelle	486	99.542	1.5471	1.07E-09	4.6988E-06
GO:0022613	ribonucleoprotein complex biogenesis	268	54.892	1.6942	2.37E-08	0.000045672
GO:0034660	ncRNA metabolic process	330	67.59	1.6127	3.11E-08	0.000045672
GO:0042254	ribosome biogenesis	162	33.181	1.8686	1.08E-07	0.00011924
GO:0006281	DNA repair	329	67.386	1.573	2.03E-07	0.0001792
GO:0140053	mitochondrial gene expression	101	20.687	2.0303	9.71E-07	0.00071234
GO:0032543	mitochondrial translation	85	17.41	2.1253	1.1317E-06	0.00071234
GO:0034470	ncRNA processing	224	45.88	1.6347	2.6492E-06	0.00111
GO:0060337	type I interferon signaling pathway	44	9.012	2.5521	2.7976E-06	0.00111
GO:0071357	cellular response to type I interferon	44	9.012	2.5521	2.7976E-06	0.00111
GO:0016072	rRNA metabolic process	143	29.289	1.8095	0.000002852	0.00111
GO:0006364	rRNA processing	122	24.988	1.8809	3.0231E-06	0.00111
GO:0006403	RNA localization	134	27.446	1.8218	4.3445E-06	0.0014724
GO:0016032	viral process	406	83.157	1.4431	4.8853E-06	0.0015375
GO:0045087	innate immune response	352	72.096	1.4703	7.2226E-06	0.0019632
GO:0034340	response to type I interferon	49	10.036	2.3914	0.000007493	0.0019632
GO:0072594	establishment of protein localization to organelle	265	54.277	1.5476	7.9216E-06	0.0019632
GO:0007005	mitochondrion organization	269	55.096	1.5428	8.0204E-06	0.0019632
GO:0043604	amide biosynthetic process	405	82.952	1.4225	0.000012415	0.0028791
GO:0044403	symbiont process	431	88.277	1.4047	0.000014448	0.0031829
GO:0043043	peptide biosynthetic process	339	69.434	1.4114	0.000095917	0.017321
GO:0022618	ribonucleoprotein complex assembly	142	29.084	1.6504	0.00012726	0.019658
GO:0050776	regulation of immune response	442	90.53	1.3476	0.00012939	0.019658
GO:0070925	organelle assembly	433	88.687	1.3418	0.00019047	0.025431

GO:0050658	RNA transport	115	23.554	1.6982	0.00023056	0.029024
GO:0006397	mRNA processing	307	62.88	1.3995	0.00029714	0.035384
GO:0055086	nucleobase-containing small molecule metabolic process	362	74.145	1.3622	0.00032404	0.037571
GO:0016071	mRNA metabolic process	432	88.482	1.3223	0.00040547	0.043573
GO:0009117	nucleotide metabolic process	316	64.723	1.3751	0.0005213	0.049971
GO:0000723	telomere maintenance	93	19.048	1.7324	0.00052696	0.049971
GO:0032200	telomere organization	93	19.048	1.7324	0.00052696	0.049971
GO:0051640	organelle localization	354	72.506	1.3516	0.00053319	0.049971
GO:0051642	centrosome localization	17	3.4819	2.872	0.00059298	0.049971

Supplemental Table S11. Tandem mass tag (TMT) labeling reagents used in each of the samples.

Sample name	TMT channel	Species	Code
Mix-1	126	Human	GM19238
Mix-1	127	Chimpanzee	CH507
Mix-1	128	Gorilla	DIAN
Mix-1	129	Macaque	R02027
Mix-1	130	Orangutan	PPY6
Mix-1	131	Pool	-
Mix-2	126	Pool	-
MIx-2	127	Human	GM12878
Mix-2	128	Chimpanzee	CH170
Mix-2	129	Gorilla	OMOYE
Mix-2	130	Macaque	R05040
Mix-2	131	Orangutan	EB185(JC)
Mix-3	126	Orangutan	CRL-1850 (PUTI)
Mix-3	127	Pool	-
Mix-3	128	Human	GM19150
Mix-3	129	Chimpanzee	CH322
Mix-3	130	Gorilla	GG05
Mix-3	131	Macaque	R94011

Supplemental Data

Supplemental Data S1. SQANTI classification files for Iso-Seq transcripts passing SQANTI quality filtering. Genomic coordinates are based on hg38, panTro5, gorGor4, ponAbe2 and rheMac8.

Supplemental Data S2. All detected peptides by mass spectrometry experiments.

Supplemental Data S3. Detected novel peptides according to RefSeq annotations. Isoleucine and leucine amino acids are designed as 'Z' since they are indistinguishable by mass spectrometry experiments.

Supplemental Data S4. BED files containing the genomic coordinates of detected novel peptides according to RefSeq annotations (hg38, panTro6, gorGor6, ponAbe3 and rheMac10). Isoleucine and leucine amino acids are designed as 'Z' since they are indistinguishable by mass spectrometry experiments.

Supplemental Data S5. GFF file for the projected isoform models in hg38 coordinates.

Supplemental Data S6. SQANTI classification file for the projected isoform models in hg38.

Supplemental Data S7. Species-specific exon gains. Genomic coordinates are based on hg38, panTro6, gorGor6, ponAbe3 and rheMac10.

Supplemental Data S8. Expression matrix for projected isoform models across samples (TPM, including batch effect correction and TMM normalization).

Supplemental Data S9. Binary expression matrix (1=presence, 0=absence) for projected isoform models across species. Only transcripts showing consistent expression in all samples from the same species are included (based on batch effect-corrected TPM values).

Supplemental Data S10. Classification of projected isoform models according to their isoform usage patterns.

Supplemental Data S11. Classification of exonic parts according to differential exon usage (DEU) results in hg38 coordinates.

Supplemental References

Alamancos GP, Pagès A, Trincado JL, Bellora N, Eyras E. 2015. Leveraging transcript quantification for fast computation of alternative splicing profiles. *RNA* **21**: 1521–1531.

Almasy L, Blangero J. 1998. Multipoint quantitative-trait linkage analysis in general pedigrees. *Am J Hum Genet* **62**: 1198–1211.

Altschul SF, Gish W, Miller W, Myers EW, Lipman DJ. 1990. Basic local alignment search tool. *Journal of Molecular Biology* **215**: 403–410.

Anders S, Pyl PT, Huber W. 2015. HTSeq--a Python framework to work with high-throughput sequencing data. *Bioinformatics* **31**: 166–169.

Anders S, Reyes A, Huber W. 2012. Detecting differential usage of exons from RNA-seq data. *Genome Res* **22**: 2008–2017.

Askenazi M, Ruggles KV, Fenyö D. 2016. PGx: Putting Peptides to BED. *J Proteome Res* **15**: 795–799.

Beer LA, Liu P, Ky B, Barnhart KT, Speicher DW. 2017. Efficient Quantitative Comparisons of Plasma Proteomes Using Label-Free Analysis with MaxQuant. *Methods Mol Biol* **1619**: 339–352.

Benjamini Y, Drai D, Elmer G, Kafkafi N, Golani I. 2001. Controlling the false discovery rate in behavior genetics research. *Behav Brain Res* **125**: 279–284.

Blum M, Chang H-Y, Chuguransky S, Grego T, Kandasaamy S, Mitchell A, Nuka G, Paysan-Lafosse T, Qureshi M, Raj S, et al. 2021. The InterPro protein families and domains database: 20 years on. *Nucleic Acids Res* **49**: D344–D354.

Bray NL, Pimentel H, Melsted P, Pachter L. 2016. Near-optimal probabilistic RNA-seq quantification. *Nat Biotechnol* **34**: 525–527.

Chiva C, Olivella R, Borràs E, Espadas G, Pastor O, Solé A, Sabidó E. 2018. QCloud: A cloud-based quality control system for mass spectrometry-based proteomics laboratories. *PLoS One* **13**: e0189209.

Csűös M. 2010. Count: evolutionary analysis of phylogenetic profiles with parsimony and likelihood. *Bioinformatics* **26**: 1910–1912.

Danecek P, Auton A, Abecasis G, Albers CA, Banks E, DePristo MA, Handsaker RE, Lunter G, Marth GT, Sherry ST, et al. 2011. The variant call format and VCFtools. *Bioinformatics* **27**: 2156–2158.

He Y, Luo X, Zhou B, Hu T, Meng X, Audano PA, Kronenberg ZN, Eichler EE, Jin J, Guo Y, et al. 2019. Long-read assembly of the Chinese rhesus macaque genome and identification of ape-specific structural variants. *Nat Commun* **10**: 4233.

Hinrichs AS, Karolchik D, Baertsch R, Barber GP, Bejerano G, Clawson H, Diekhans M, Furey TS, Harte RA, Hsu F, et al. 2006. The UCSC Genome Browser Database: update 2006. *Nucleic Acids Res* **34**: D590-598.

Kronenberg ZN, Fiddes IT, Gordon D, Murali S, Cantsilieris S, Meyerson OS, Underwood JG, Nelson BJ, Chaisson MJP, Dougherty ML, et al. 2018. High-resolution

comparative analysis of great ape genomes. *Science* **360**. http://dx.doi.org/10.1126/science.aar6343.

Kuo RI, Cheng Y, Zhang R, Brown JWS, Smith J, Archibald AL, Burt DW. 2020. Illuminating the dark side of the human transcriptome with long read transcript sequencing. *BMC Genomics* **21**: 751.

Li H, Handsaker B, Wysoker A, Fennell T, Ruan J, Homer N, Marth G, Abecasis G, Durbin R, 1000 Genome Project Data Processing Subgroup. 2009. The Sequence Alignment/Map format and SAMtools. *Bioinformatics* **25**: 2078–2079.

Love MI, Huber W, Anders S. 2014. Moderated estimation of fold change and dispersion for RNA-seq data with DESeq2. *Genome Biol* **15**: 550.

Mao Y, Catacchio CR, Hillier LW, Porubsky D, Li R, Sulovari A, Fernandes JD, Montinaro F, Gordon DS, Storer JM, et al. 2021. A high-quality bonobo genome refines the analysis of hominid evolution. *Nature* **594**: 77–81.

McAlister GC, Nusinow DP, Jedrychowski MP, Wühr M, Huttlin EL, Erickson BK, Rad R, Haas W, Gygi SP. 2014. MultiNotch MS3 enables accurate, sensitive, and multiplexed detection of differential expression across cancer cell line proteomes. *Anal Chem* **86**: 7150–7158.

Perkins DN, Pappin DJ, Creasy DM, Cottrell JS. 1999. Probability-based protein identification by searching sequence databases using mass spectrometry data. *Electrophoresis* **20**: 3551–3567.

Quinlan AR, Hall IM. 2010. BEDTools: a flexible suite of utilities for comparing genomic features. *Bioinformatics* **26**: 841–842.

Ramírez F, Ryan DP, Grüning B, Bhardwaj V, Kilpert F, Richter AS, Heyne S, Dündar F, Manke T. 2016. deepTools2: a next generation web server for deep-sequencing data analysis. *Nucleic Acids Res* **44**: W160–W165.

Reyes A, Anders S, Weatheritt RJ, Gibson TJ, Steinmetz LM, Huber W. 2013. Drift and conservation of differential exon usage across tissues in primate species. *Proceedings of the National Academy of Sciences* **110**: 15377–15382.

Rice P, Longden I, Bleasby A. 2000. EMBOSS: The European Molecular Biology Open Software Suite. *Trends in Genetics* **16**: 276–277.

Ritchie ME, Phipson B, Wu D, Hu Y, Law CW, Shi W, Smyth GK. 2015. limma powers differential expression analyses for RNA-sequencing and microarray studies. *Nucleic Acids Res* **43**: e47.

Salmela L, Rivals E. 2014. LoRDEC: accurate and efficient long read error correction. *Bioinformatics* **30**: 3506–3514.

Schafer S, Miao K, Benson CC, Heinig M, Cook SA, Hubner N. 2015. Alternative Splicing Signatures in RNA-seq Data: Percent Spliced in (PSI). *Curr Protoc Hum Genet* **87**: 11.16.1-11.16.14.

Shen W, Le S, Li Y, Hu F. 2016. SeqKit: A Cross-Platform and Ultrafast Toolkit for FASTA/Q File Manipulation. *PLOS ONE* **11**: e0163962.

Shumate A, Salzberg SL. 2020. Liftoff: accurate mapping of gene annotations. *Bioinformatics* **37**: 1639–1643.

Soneson C, Love MI, Robinson MD. 2015. Differential analyses for RNA-seq: transcript-level estimates improve gene-level inferences. *F1000Res* **4**: 1521.

Tang S, Lomsadze A, Borodovsky M. 2014. Identification of protein coding regions in RNA transcripts. *Nucleic Acids Research* **43**: e78.

Tardaguila M, de la Fuente L, Marti C, Pereira C, Pardo-Palacios FJ, Del Risco H, Ferrell M, Mellado M, Macchietto M, Verheggen K, et al. 2018. SQANTI: extensive characterization of long-read transcript sequences for quality control in full-length transcriptome identification and quantification. *Genome Res* **28**: 396–411.

The 1000 Genomes Project Consortium. 2015. A global reference for human genetic variation. *Nature* **526**: 68–74.

Vitting-Seerup K, Sandelin A. 2019. IsoformSwitchAnalyzeR: analysis of changes in genome-wide patterns of alternative splicing and its functional consequences. *Bioinformatics* **35**: 4469–4471.

Vitting-Seerup K, Sandelin A. 2017. The Landscape of Isoform Switches in Human Cancers. *Mol Cancer Res* **15**: 1206–1220.

Wilks C, Zheng SC, Chen FY, Charles R, Solomon B, Ling JP, Imada EL, Zhang D, Joseph L, Leek JT, et al. 2021. recount3: summaries and queries for large-scale RNA-seq expression and splicing. *Genome Biol* **22**: 323.

Wu TD, Watanabe CK. 2005. GMAP: a genomic mapping and alignment program for mRNA and EST sequences. *Bioinformatics* **21**: 1859–1875.

Wyman D, Mortazavi A. 2019. TranscriptClean: variant-aware correction of indels, mismatches and splice junctions in long-read transcripts. *Bioinformatics* **35**: 340–342.

Yates AD, Achuthan P, Akanni W, Allen J, Allen J, Alvarez-Jarreta J, Amode MR, Armean IM, Azov AG, Bennett R, et al. 2020. Ensembl 2020. *Nucleic Acids Res* **48**: D682–D688.

Zhang S-J, Wang C, Yan S, Fu A, Luan X, Li Y, Sunny Shen Q, Zhong X, Chen J-Y, Wang X, et al. 2017. Isoform Evolution in Primates through Independent Combination of Alternative RNA Processing Events. *Mol Biol Evol* **34**: 2453–2468.

Zhang Y, Parmigiani G, Evan Johnson W. 2020. ComBat-Seq: batch effect adjustment for RNA-Seq count data. *NAR Genomics and Bioinformatics* **2**: lqaa078.
